# Analysis of the *TaNAS* gene family in bread wheat identifies additional members and candidates for intragenic biofortification

**DOI:** 10.1093/jxb/erag242

**Published:** 2026-05-22

**Authors:** Oscar Carey-Fung, Jesse T Beasley, Lily Tarry-Smith, Edbert Josua Felix, Nathan Leckie, Damien L Callahan, Rudi Appels, Alexander A T Johnson

**Affiliations:** School of BioSciences, The University of Melbourne, Parkville, VIC 3010, Australia; School of BioSciences, The University of Melbourne, Parkville, VIC 3010, Australia; School of BioSciences, The University of Melbourne, Parkville, VIC 3010, Australia; School of Agriculture, Food and Ecosystems Sciences, The University of Melbourne, Parkville, VIC 3010, Australia; School of BioSciences, The University of Melbourne, Parkville, VIC 3010, Australia; School of Life and Environmental Science, Deakin University, Burwood, VIC 3125, Australia; School of Agriculture, Food and Ecosystems Sciences, The University of Melbourne, Parkville, VIC 3010, Australia; School of BioSciences, The University of Melbourne, Parkville, VIC 3010, Australia; CIMMYT, Mexico

**Keywords:** Biofortification, Fe deficiency, intragenesis, iron, nicotianamine synthase, zinc

## Abstract

Higher plants utilize nicotianamine synthase (NAS) enzymes to produce nicotianamine (NA), a non-protein amino acid that chelates metals such as Fe and Zn, and plays key roles in metal transport and homeostasis. We identified 34 *TaNAS* genes in the recently annotated cv. Chinese Spring bread wheat genome (IWGSC Refseq 1.1 and 2.1), and four cultivar-specific *TaNAS* genes in the 10+ Wheat Genome Project, representing a significant expansion on previous analyses of the *TaNAS* gene family. The majority of *TaNAS* genes were highly expressed in roots and up-regulated in response to hydroponic Fe deficiency. The TaNAS proteins ranged from 180 to 384 amino acids and showed variable N- and C-termini. To investigate TaNAS function, we transformed bread wheat cv. Fielder with constitutive overexpression constructs of *TaNAS1*, *TaNAS3*, *TaNAS4*, *TaNAS6*, or *TaNAS7*, and isolated single-locus homozygous events and null segregant (NS) controls for glasshouse and field evaluation. Under field conditions, grain Fe, Zn, and NA concentrations were up to 1.6-, 1.8-, and 3.7-fold higher, respectively, in *TaNAS6* events relative to NS with no detrimental impact on agronomic traits. Transient expression analyses in *Nicotiana benthamiana* demonstrated that TaNAS6 produced significantly more NA in leaf tissue relative to other NAS enzymes. These results broaden our understanding of the *TaNAS* gene family in bread wheat and highlight novel biofortification strategies to improve grain nutrition.

## Teaser

This study describes the identification of 38 *TaNAS* genes in bread wheat, and highlights the *TaNAS6-D1* homoeolog as a promising candidate for intragenic biofortification of bread wheat with iron and zinc.

## Introduction

Nicotianamine (NA) is a non-protein amino acid that was originally purified from tobacco (*Nicotiana tabacum* L.), where it was shown to be critical for maintaining iron (Fe) homeostasis and in turn fundamental biological processes such as chlorophyll biosynthesis (Noma *et al.*, 1971; Ripperger, 1986). Nicotianamine synthase (NAS), the enzyme responsible for catalyzing the trimerization of *S*-adenosylmethionine (SAM) into NA, was first identified in the *chloronerva* tomato mutant, which exhibits poor growth and leaf chlorosis due to a complete loss of the ability to synthesize NA (Higuchi *et al.*, 1996; Ling *et al.*, 1999). It is now widely understood that NA is ubiquitous to all higher plants and chelates a range of divalent metal cations including Fe^2+^, zinc (Zn^2+^), manganese (Mn^2+^), and nickel (Ni^2+^) for their long-distance transport between plant tissues (Von Wirén *et al.*, 1999; Vacchina *et al.*, 2003; Koike *et al.*, 2004).

In graminaceous species, the *NAS* genes are classified into two clades. Clade I *NAS* genes are predominantly expressed in root tissues where NA facilitates long-distance transport of metals from source (i.e. roots and leaves) to sink (i.e. grain) tissues through the phloem, and uptake of Fe from the rhizosphere by serving as the biosynthetic precursor to 2′-deoxymugineic acid (DMA) via nicotianamine aminotransferase (NAAT) and 2′-deoxymugineic acid synthase (DMAS). In bread wheat, DMA is secreted into the rhizosphere by the transporter of mugineic acid 1 (TOM1), and Fe–DMA complexes are taken up into the roots by members of the yellow stripe-like (YSL) transporter family (Inoue *et al.*, 2009; Nozoye *et al.*, 2011). By contrast, Zn–DMA complexes are crucial for Zn translocation within the plant rather than Zn uptake from the soil (Suzuki *et al.*, 2008). Instead, soil Zn uptake is mediated mainly by Zn/Fe-regulated transporter-like protein (ZIP) transporters such as the OsZIP9 transporter in rice (*Oryza sativa* L.) (Huang *et al.*, 2020; Yang *et al.*, 2020). Other OsZIP proteins are crucial for transporting Zn between rice tissues, and it is expected that the ZIP proteins in bread wheat play similar roles (Lee *et al.*, 2010a, b; Sasaki *et al.*, 2015; Li *et al.*, 2021). In rice and *Arabidopsis halleri*, NA is proposed to form stable Zn–NA complexes in root cells where symplastic movement to the xylem allows for Zn–NA accumulation in the shoots and seeds (Lee *et al.*, 2011; Deinlein *et al.*, 2012). Clade II *NAS* genes are primarily expressed in shoot tissues and under conditions of Fe excess, where they maintain cellular Fe homeostasis in part by regulating short-distance cytoplasmic distribution (Aung *et al.*, 2019; Seregin and Kozhevnikova, 2023). In the metal hyperaccumulator *A. halleri*, NA accumulation and secretion confer tolerance to conditions of Zn excess (Tsednee *et al.*, 2014). Within the cell, NA plays a critical role in the sequestration of excess metal ions into vacuoles, thereby preventing the formation of reactive oxygen species (ROS) (Pich *et al.*, 2001). During the digestion of plant-based foods, NA plays a role in enhancing dietary Fe bioavailability, and Caco-2 (human intestinal epithelial) cells show greater absorption of Fe when in solution with NA relative to known enhancers of Fe bioavailability (e.g. ascorbic acid or epicatechin) (Zheng *et al.*, 2010; Lee *et al.*, 2012; Eagling *et al.*, 2014; Beasley *et al.*, 2019b). Furthermore, mice that were fed rice containing high concentrations of NA recovered more quickly from Fe-deficiency anaemia relative to those fed conventional rice (Lee *et al.*, 2012).

Increased expression of *NAS* genes is a widely utilized and successful strategy for Fe and Zn biofortification of a variety of plant species. In rice, heterologous expression of *HvNAS1* from barley (*Hordeum vulgare* L.) increased grain Fe, Zn, and NA concentrations by up to 3-, 2-, and 10.6-fold, respectively (Masuda *et al.*, 2009). Also in rice, constitutive overexpression of the endogenous *OsNAS2* increased grain Fe, Zn, and NA concentrations by up to 4-, 2-, and 9.3-fold, respectively (Johnson *et al.*, 2011), constitutive overexpression of *OsNAS3* increased grain Fe, Zn, and NA concentrations by up to 2.9-, 2.2-, and 9.6-fold, respectively (Lee *et al.*, 2009), and constitutive overexpression of both *OsNAS2* and *OsNAS3* increased grain Fe, Zn, and NA concentrations by up to 4-, 3.5-, and 50.6-fold, respectively (Lee *et al.*, 2023). In bread wheat (*Triticum aestivum* L.), heterologous expression of *OsNAS2* increased grain Fe, Zn, and NA concentrations up to 1.5-, 1.6- and 15-fold, respectively, under glasshouse conditions and 1.3-, 1.4-, and 2.9-fold, respectively, under field conditions (Beasley *et al.*, 2019a). In tobacco, heterologous expression of *HvNAS1* increased seed Fe and Zn concentrations by 2.3- and 1.8-fold, respectively, and heterologous expression of the apple (*Malus xiaojinensis*) *MxNAS1* and *MxNAS2* genes increased leaf NA content ∼3.5-fold and improved plant tolerance to Fe deficiency (Takahashi *et al*., 2003; Han *et al*., 2013; Yang *et al*., 2015). Heterologous expression of the Arabidopsis (*Arabidopsis thaliana*) *AtNAS1* gene in potato (*Solanum tuberosum* L.) increased tuber Fe concentration by 2.4-fold (Zha *et al.*, 2022).

The number of *NAS* genes varies widely among plant species, with rice containing three *OsNAS* genes, maize (*Zea mays* L.) nine *ZmNAS* genes, barley seven *HvNAS* genes, and bread wheat originally reported to contain 21 *TaNAS* genes (Higuchi *et al.*, 1999; Inoue *et al.*, 2003; Zhou *et al.*, 2013; Bonneau *et al.*, 2016). Here we describe the identification of 38 *TaNAS* genes in the recently annotated cv. Chinese Spring bread wheat genome (IWGSC Refseq 1.1 and 2.1) and the 10+ Wheat Genome Project, representing a significant increase on previous studies of the *TaNAS* gene family (Walkowiak *et al.*, 2020; Zhu *et al.*, 2021). Furthermore, we demonstrate that constitutive overexpression of individual *TaNAS* genes, particularly the *TaNAS6-D1* homoeologue, holds potential as an intragenic means of biofortifying bread wheat with Fe and Zn.

## Materials and methods

### Identification and annotation of *TaNAS* genes

Previously annotated *NAS* coding sequences (CDSs) from *A. thaliana*, *H. vulgare* L., *O. sativa* L., *T. aestivum* L., and *Z. mays* L. were used as queries to blast the IWGSC Refseq1.1 and IWGSC Refseq 2.1 in EnsemblPlants (https://plants.ensembl.org/index.html), leading to the identification of 43 potential *TaNAS* genes in cv. Chinese Spring (Supplementary Tables S1, S2, S3) (Bolser *et al.*, 2015; Howe *et al.*, 2020). Additional *TaNAS* genes in the 10+ Wheat Genome Project were identified in cultivars ArinaLrFor, Julius, Lancer, Norin61, SY Mattis, Mace, Jagger, Landmark, and Stanley, and in *Triticum spelta* L. by blasting the *TaNAS* CDSs from cv. Chinese Spring against each of these genomes on EnsemblPlants (Appels *et al.*, 2018; Zhu *et al.*, 2021). All *TaNAS* genes in cv. Fielder were identified using Earlham Institute’s Grassroots Infrastructure BLAST tool (https://grassroots.tools/service/blast-blastn) (Bian *et al.*, 2017). The *TaNAS4-D1* and *TaNAS4-D2* homoeologues in cultivars ArinaLrFor, Julius, Lancer, Norin61, SY Mattis, Mace, Stanley, and Fielder, and the *TaNAS7-A1* homoeologue in cv. Jagger could not be identified on a chromosome. The cv. Chinese Spring *TaNAS* CDSs were annotated following visualization of the *TaNAS* genes in the Apollo database (https://apollo-portal.genome.edu.au/), which integrates the Refseq2.1 reference genome sequence, models from NCBI, and RNA-seq data (Dunn *et al.*, 2019; Walkowiak *et al.*, 2020; Zhu *et al.*, 2021). Only the CDS of each *TaNAS* gene was annotated, as the 5′- and 3′-untranslated region (UTR) annotations were often inconsistent between the Refseq1.1, Refseq2.1, and NCBI models, and did not necessarily match the RNA-seq read expression profiles. Any discrepancies between Refseq1.1, Refseq2.1, and NCBI annotations in the CDSs of *TaNAS* genes were resolved using the RNA-seq datasets integrated into Apollo and the RNA-seq dataset generated in this study (Supplementary Table S2; Supplementary Fig. S1B). Additional Ribo-seq data to resolve the presence of a putative N-terminal extension in TaNAS6-D1 was sourced from Guo *et al*. (2023) (Supplementary Fig. S1A). The Phyre2 (v2.0) protein fold recognition software was used to predict the alignment of TaNAS proteins with the c3fpjA_ template, corresponding to alignment with the 3D structure of an archaeal NAS protein from *Methanothermobacter thermautotrophicus* in the Protein Data Bank (PDB) (Dreyfus *et al.*, 2009; Kelley *et al.*, 2015). Nine potential *TaNAS* genes in cv. Chinese Spring were disqualified as they either did not contain the c3pjA_ protein structure and/or did not respond to Fe deficiency (Supplementary Table S2). Bread wheat, einkorn wheat, barley, maize, and rice *NAS* CDSs were aligned using the Geneious alignment tool (v2024.0.7), and phylogenetic construction was performed using the PhyML Geneious plugin with the K80: Kimura substitution model and 1000 bootstrap replications (Guindon *et al.*, 2010). Protein sequences were aligned using the Geneious alignment tool (v2024.0.7).

### Bread wheat transformation

Individual *TaNAS* CDSs were PCR amplified from bread wheat genomic DNA (cv. Chinese Spring) using Phusion™ high-fidelity Taq polymerase (Thermo Fisher Scientific, Waltham, MA, USA) and cloned into the pENTR vector (Karimi *et al.*, 2007). Each pENTR vector containing a *TaNAS* CDS was digested with *Ase*I (New England Biolabs, Ipswich, MA, USA) and recombined (LR reaction) into a modified pMDC32 vector containing a maize ubiquitin 1 promoter (Ubi-1) to drive expression of the *TaNAS* CDS as well as the hygromycin resistance (*hptII*) gene for transformation selection (Curtis and Grossniklaus, 2003). Bread wheat transformation was performed at the University of Adelaide using the *Agrobacterium tumefaciens* strain AGL1 as previously described (Okada *et al.*, 2019). Briefly, immature bread wheat embryos were harvested from cv. Fielder plants 14 d post-anthesis and co-cultured with *Agrobacterium* containing the pMDC32 vector. Embryonic axes from the embryos were removed following co-culture. Resting, callus induction, selection, and regeneration were performed over 10 weeks prior to soil transfer in a glasshouse. Each plantlet derived from callus tissue was considered an individual event. Harvested T_1_ seeds from mature plants of each event were sent to the University of Melbourne.

### Isolation of single-locus homozygous lines

Sixteen T_1_ grains from 10–15 events per *TaNAS* construct were stratified (4 °C for 2 d) and germinated in 56-well trays containing sifted potting mix (pine bark, peat, and sand mixture) with Osmocote® fertilizer (4.5 g l^–1^). Approximately 1 cm of leaf was harvested and placed in a 96-well plate. Genomic DNA was extracted by adding 50 µl of Extract-N-Amp extraction solution (Merck, Darmstadt, Germany), incubating the samples at 95 °C for 10 min and adding 50 µl of Extract-N-Amp dilution solution. Melt curve analysis was performed using 1 µl of DNA template, 5 µl of KAPA SYBR® FAST (Merck), 0.2 µl of primer (10 µM), and 10 µl of nuclease-free H_2_O. The primers used to amplify *hptII* in the T-DNA and *TaCyclophilin* (housekeeping gene) as a reaction control are given in Supplementary Table S4. PCR conditions included initial denaturation at 95 °C for 3 min, and 35 cycles of: (i) denaturation at 95 °C for 20 s; (ii) primer annealing at 63 °C for 20 s; and (iii) fluorescence measurement by plate reading. A melt curve was generated by incubating the samples from 78 °C to 90 °C in 0.1 °C increments for 5 s at each increment. For T_1_ populations with a transgene negative:positive ratio of 1:3, the event was determined to be single locus, and two null segregants (NSs) and six transgenics were grown to maturity. Sixteen T_2_ progeny grain were placed on moist filter paper for 2 d and transferred into wells of a 96-well plate where the grain were genotyped by the method described above. A T_1_ parent was determined to be homozygous if all T_2_ progeny were transgenic positive. A T_1_ NS sibling from each single-locus event was also advanced for glasshouse and field experiments to control for the effects of tissue culture (Miyao *et al.*, 2012).

### Glasshouse, speed breeding, and field growth conditions

Glasshouse growth of T_1_, T_3_, and T_4_ plants and speed breeding of T_2_ plants was performed on the Parkville Campus at The University of Melbourne, Victoria, Australia. Either 1 litre (glasshouse) or 8.1 litre (speed breeding) pots containing general potting mix with Osmocote® fertilizer (4.5 g l^–1^) were used for plant growth. Plants were watered using drip irrigation (∼50 ml) once per day. Approximately 10 ml of ammonium nitrate (NH_4_NO_3_ solution, 28.57 g l^–1^) fertilizer was added to the glasshouse plants 6 weeks and 9 weeks after sowing. In the glasshouse, plants were sown in May/June and harvested in December/January. Speed-bred plants were grown under a 22 h day (22 °C) and 2 h night (17 °C) cycle with 65% humidity and Heliospectra LED Grow Lights programmed to 400–600 µmol m^–2^ s^–1^ photosynthetic photon flux densities at pot height (Heliospectra, Gothenburg, Sweden) (Watson *et al.*, 2018). Field experiments were performed at Dookie Campus at The University of Melbourne, Victoria, Australia. Each plot was comprised of six rows (75 cm long) spaced 25 cm apart and grain was sown at a density of 6 g (2022) or 10 g (2023) per m^2^. At sowing, plots were fertilized with di-ammonium phosphate (DAP) at a rate of 100 kg ha^–1^, and urea was applied at a rate of 150 kg ha^–1^ during tillering and stem elongation stages. Plants relied on rainfall throughout the growing seasons. Agronomic traits of field-grown plants were determined by harvesting and phenotyping one randomly selected representative row of the four internal rows per plot and multiplying by 6 to calculate biomass, grain weight, or tiller number per m^2^.

### RNA-sequencing of hydroponically grown plants

Grain harvested from cv. Fielder plants were stratified at 4 °C for 2 d and then surface-sterilized with VitaVax 200FF (Hewitt & Whitty, Geelong, Victoria, Australia) and placed on sterile moist filter paper at 20 °C for 2 d. Germinated seedlings were transferred to hydroponic boxes containing a nutrient-replete solution: 2 mM NH_4_NO_3_, 5 mM KNO_3_, 2 mM Ca(NO_3_)_2_·4H_2_O, 2 mM MgSO_4_·7H_2_O, 0.1 mM KH_2_PO_4_, 10 µM H_3_BO_3_, 5 µM MnCl_2_·4H_2_O, 5 µM ZnSO_4_·7H_2_O, 0.5 µM CuSO_4_·5H_2_O, 0.1 µM Na_2_MoO_4_·2H_2_O, and 50 µM NaFe^3+^EDTA. Following 12 d of nutrient-replete growth, the solution was replaced with an Fe-deficient solution (0 µM NaFe^3+^EDTA) and grown for a further 11 d. The nutrient solution was replaced every 4 d. Leaf tissues were harvested at days 0, 1, 7, and 11 of Fe deficiency, and root tissues were harvested at day 11 of Fe deficiency. All tissues were immediately snap-frozen in liquid nitrogen following sampling. Approximately 50 mg of tissue in TRI Reagent® (Merck) was homogenized with stainless steel beads and a TissueLyser II (Qiagen, Hilden, Germany). The Direct-zol RNA Miniprep Kit (Zymo Research, Irvine, CA, USA) was used to extract RNA according to the manufacturer’s instructions. Quality control and RNA-seq were performed by the Australian Genome Research Facility (AGRF, Melbourne, Victoria, Australia). Briefly, sample quality was assessed using the Agilent 4200 TapeStation and Agilent RNA ScreenTape (Agilent Technologies, Santa Clara, CA, USA) and reagents according to the manufacturer’s instructions. Sample concentrations were measured using the QuantiFluor RNA Kit (Promega, Madison, WI, USA). Library preparation was performed using the Illumina Stranded mRNA library preparation workflow and sequencing was completed with the Illumina NovaSeq X Plus with 150 paired-end reads and one lane of a 10B-300 flow cell according to the manufacturer’s instructions (Illumina, San Diego, CA, USA).

### Gene expression analysis

Quantitative reverse transcription–PCR analysis of each *TaNAS* transgene was performed on 5-day-old seedlings. Shoot tissue above the crown from three plants per *TaNAS* event was individually harvested and snap-frozen in liquid nitrogen and homogenized in TRIzol (Thermo Fisher, Carlsbad, CA, USA). Total RNA was extracted using the Direct-zol RNA Miniprep Kit (Zymo Research), according to the manufacturer’s instructions. All RNA samples were normalized to 83 ng µl^–1^ and genomic DNA was removed using the RQ1 RNase-Free DNAse kit (Promega), according to the manufacturer’s instructions. Reverse transcription was performed using the Tetro cDNA Synthesis kit (Bioline, Meridian Bioscience, Memphis, TN, USA), according to the manufacturer’s instructions. Gene expression was quantified using KAPA SYBR® FAST qPCR Master Mix (2X) Kit (Sigma Aldrich, St. Louis, MO, USA) with three technical replicates per biological replicate. Expression of the *TaNAS* transgene was determined using primers within the *attB2* cloning site and 5′ end of the *nosT* (Supplementary Table S4). Expression of the *TaNAS* transgene was normalized against the geometric mean of three housekeeping genes: *TaACT*, *TaGAPDH*, and *TaSAR* (Supplementary Table S4). Analysis of Ct values was performed using the 2^−ΔΔCt^ method and normalized against *TaNAS1-1* (Livak and Schmittgen, 2001).

Analysis of RNA-seq data was performed in Galaxy Australia (v1.0.0). Paired-end files from each sample and lane were concatenated (Galaxy v1.0.0) to generate single files for each read pair (R1 and R2). Quality checks were performed using FastQC (v0.74+galaxy1) and summarized with MultiQC (v1.11+galaxy1). Adapter trimming and low-quality base removal were done with Trim Galore (v0.6.7+galaxy0), applying a Phred score threshold of 10 and discarding reads <30 bp. Post-trimming quality was reassessed with FastQC. Reads were aligned to the ‘161010_Chinese_Spring_v1.0_pseudomolecules.fasta’ reference genome (https://urgi.versailles.inra.fr/) using HISAT2 (v2.2.1+galaxy1), with paired-end and reverse strand (RF) options. Transcripts were assembled with StringTie (v2.2.3+galaxy0) using the HISAT2 BAM output, the reference high-confidence GTF file (IWGSC_v1.1_HC_20170706.gtf), and the low-confidence GTF file (IWGSC_v1.1_LC_20170706.gtf) to capture all low-confidence *TaNAS* genes (https://urgi.versailles.inra.fr/). Gene counts were quantified with FeatureCounts (v2.0.3+galaxy2), creating a count and gene length matrix for downstream expression analysis. Count and length matrices were loaded into Rstudio (v4.4.1) to calculate transcripts per million (TPM) for each sample. Calculations followed standard protocols using the dplyr package (v1.1.4), where raw counts were divided by gene lengths in kilobases to generate reads per kilobase (RPK). The total RPK were summed to create a scaling factor for each sample. TPM values were then obtained by dividing RPK values by this scaling factor and multiplying by 10^6^, ensuring comparability across samples. This approach was applied to three biological replicates across five different conditions for both high-confidence and low-confidence annotations.

### Elemental analysis

Harvested spikes from the glasshouse and field experiments were cleaned and dried in an oven (30 °C) for at least 2 d. Grain samples (∼3 g) from each plant were ground (60 s, 25 000 rpm) into wholegrain flour using a tube-mill (Tube Mill Control, IKA, Staufen, Germany). Wholegrain flour mineral concentrations were analyzed by inductively coupled plasma optical emission spectroscopy (ICP-OES) at The University of Melbourne’s Trace Analysis for Chemical, Earth and Environmental Sciences (TrACEES) platform as previously described (Wheal *et al.*, 2011). Flour samples (0.225–0.250 g) were digested in batches (*n*=40) using either nitric acid (2 ml, 69%)/hydrogen peroxide (0.5 ml, 30%) or nitric acid (7 ml, 69%)/hydrogen peroxide (1 ml, 30%) solutions in a heat block for 2 h at 125 °C or a microwave digester (ETHOS™ EASY, Metrohm Australia, Mitcham, VIC, Australia) for 30 min at 200 °C, respectively. Samples had a final nitric acid concentration of 9%. Analysis was performed on a Perkin Elmer 8300 DV ICP-OES (Syngystix v3.0) relative to a 9% nitric acid sample matrix and internal standards for each element.

### Nicotianamine and 2′-deoxymugineic acid analysis

Grain NA and DMA quantification was performed using reverse phase LC (Selby-Pham *et al.*, 2017; Beasley *et al.*, 2019a, 2022). Briefly, 25 mg of ground wholegrain flour was combined with 500 µl of methanol (Sigma Aldrich) in a 2 ml tube and incubated in a thermoshaker (TS-100 Thermoshaker, Biosan, Riga, Latvia) at 30 °C and 1400 rpm for 15 min. The tubes were centrifuged (16 300 *g*, 10 min), and the supernatant was transferred into a new 2 ml tube. The original pellet was resuspended in 500 µl of MilliQ H_2_O via vortexing, and centrifuged (16 300 *g*, 10 min). The resulting supernatant was combined with the methanol supernatant from the previous step. The combined extract was centrifuged prior to storage at −20 °C. After thawing, the extract was centrifuged and an aliquot (5 µl) was combined with 10 µl of sodium borate buffer (1 M; Merck), 10 µl of EDTA (50 mM; Merck), and 40 µl of fluorenylmethyloxycarbonyl-chloride (FMOC-Cl, 50 mM; Merck), and incubated in a thermoshaker at 60 °C and 700 rpm for 15 min. The derivatized product was centrifuged and combined with 8.9 µl of formic acid (5%, Tokyo Chemical Industry, Chuo City, Tokyo, Japan) and vortexed to quench the reaction. Derivatized samples were analyzed by LC-MS using a Zorbax Eclipse XDB-C18 Rapid Resolution HS 2.1×100 mm, 1.8 µM particle size column (Agilent Technologies Inc.) and a triple quadrupole LC-MS system (Shimadzu-8050) with electrospray ionization in positive ion MRM (multiple reaction monitoring) mode. A binary mobile phase system was used for separation with 0.1% formic acid in MilliQ H_2_O (aqueous) and 0.1% formic acid in 100% acetonitrile (organic) mobile phases (Merck). Quantification was carried out using an external calibration curve with standards from 0.075 µM to 75 µM prepared from 750 µM aqueous NA and DMA stocks (Toronto Research Chemicals, Toronto, ON, Canada). Peak area values were calculated using LabsSolutions Insight software (https://www.shimadzu.com/) and Microsoft Excel 170 (https://www.microsoft.com/en-us/microsoft-365/excel). Pooled biological quality control samples and blanks were run, and the system was re-calibrated every 100 samples. Samples were randomized prior to running.

### Transient *NAS* expression in *Nicotiana benthamiana*

Modified pMDC32 vectors containing the *TaNAS1*, *TaNAS3*, *TaNAS6*, *TaNAS6S*, or *OsNAS2* CDS downstream of the maize Ubi-1 promoter were transformed into *A. tumefaciens* (GV3101) via electroporation. The pGD-p19 vector (Addgene plasmid #196326; http://n2t.net/addgene:196326; RRID: Addgene_196326) was also transformed into *A. tumefaciens* (GV3101) via electroporation to reduce post-transcriptional silencing during transient expression experiments in *N. benthamiana* leaves (Ganesan *et al.*, 2013). Individual *A. tumefaciens* colonies were grown in liquid medium and resuspended in agroinfiltration solution containing MgCl_2_ (10 mM), MES buffer (10 mM), and acetosyringone (200 μM) (Sigma-Aldrich). Following incubation for 2 h at 100 rpm (room temperature, no light), each pMDC32-*NAS* culture was normalized to 0.2 OD_600_ and combined with the pGD-p19 culture at a 1:1 ratio (10 ml total).

*Nicotiana benthamiana* plants were grown for 4 weeks at 25 °C with a 16 h light period (70–80% humidity). Agroinfiltration solutions containing pGD-p19 and individual pMDC32-*NAS* vectors were infiltrated into the underside of six leaves from three *N. benthamiana* plants. Each leaf was infiltrated in at least three sites. A pGD-p19 agroinfiltration solution with no pMDC32-*NAS* vector was also infiltrated into *N. benthamiana* to enable quantification of endogenous NA concentration in *N. benthamiana* leaves. Following 6 d of growth, three 10 mm diameter leaf discs with a combined weight of 30–50 mg were cut from individual infiltration areas, with each one representing a single biological replicate. The three leaf discs were homogenized in 500 µl of methanol using a Lysing Matrix containing 1.4 mm zirconium-silicate beads (MP Biomedicals, Santa Ana, CA, USA) and a TissueLyser II (Qiagen). NA extraction and quantification followed the protocol described in the previous NA and DMA analysis method.

## Results

### Identification of 34 *TaNAS* genes in bread wheat across different chromosomes and cultivars

**Fig. 1.**
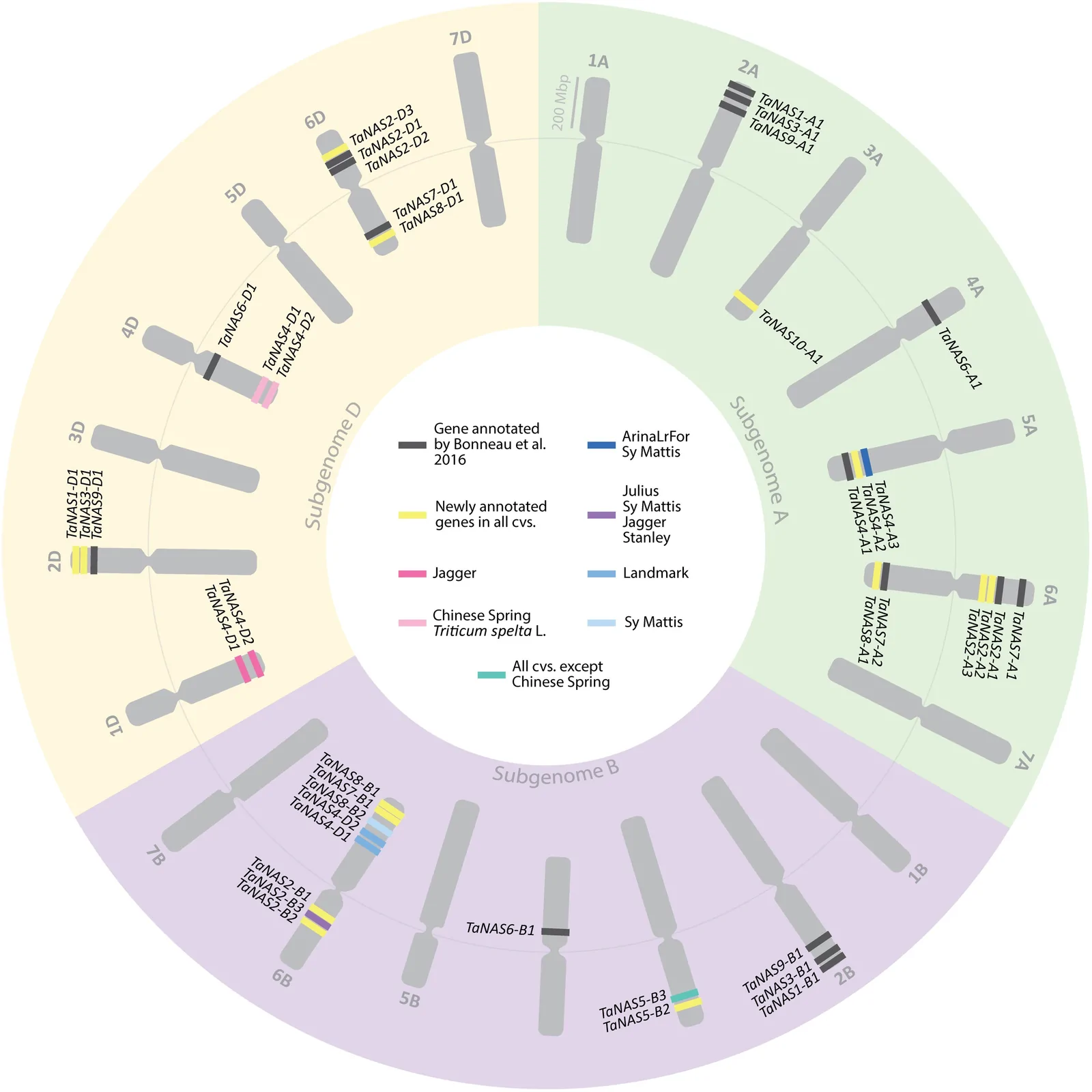
Location of *TaNAS* genes across 11 bread wheat (*Triticum aestivum* L.) cultivars and *Triticum spelta* L. Chromosomes within the A (green), B (purple), and D (orange) subgenomes of bread wheat are depicted clockwise. Bread wheat *TaNAS* genes that were previously annotated in Bonneau *et al*. (2016) (black), newly annotated in all cultivars (yellow), or exclusively present in cv. Jagger (dark pink); cv. Chinese Spring and cv. *Triticum spelta* L. (light pink); cv. ArinaLrFor and cv. SY Mattis (dark blue); cv. Julius, cv. SY Mattis, cv. Jagger, and cv. Stanley (purple); cv. Landmark (blue); cv. SY Mattis (light blue); or all cultivars except for cv. Chinese Spring (teal) are indicated in approximate genomic regions of each chromosome.

Thirty-four *TaNAS* genes were identified in the reference bread wheat genome (cv. Chinese Spring), including 14 that were previously uncharacterized (Fig. 1; Supplementary Table S1). Average CDS identity for the 34 *TaNAS* genes was 78.8% and ranged from 61.8% to 100%. The *TaNAS4-D1* and *TaNAS4-D2* homoeologues had the same CDS, possibly due to a recent intrachromosomal gene duplication event. All 34 *TaNAS* genes were present in the genomes of cultivars ArinaLrFor, Julius, Lancer, Norin61, SY Mattis, Mace, Jagger, Landmark, Stanley, and Fielder, and 33 *TaNAS* genes (all apart from *TaNAS7-A1*) were present in the genome of *Triticum spelta* L. Four additional *TaNAS* genes (*TaNAS2-B3*, *TaNAS4-A3*, *TaNAS5-B3*, and *TaNAS8-B2*) were identified in the cultivars analyzed but not in cv. Chinese Spring (Supplementary Table S5). The genomic location and presence of the *TaNAS* genes varied across cultivars (Fig. 1). Cultivars ArinaLrFor and SY Mattis contained an additional *TaNAS* gene on chromosome 5A (*TaNAS4-A3*), which shared >95% CDS identity with the *TaNAS4-A1*, *TaNAS4-A2*, *TaNAS4-D1*, and *TaNAS4-D2* homoeologues (Fig. 1; Supplementary Table S5). Cultivars Julius, SY Mattis, Jagger, and Stanley contained an additional *TaNAS* gene on chromosome 6B (*TaNAS2-B3*), which shared >95% CDS identity with the *TaNAS2-A1*, *TaNAS2-A2*, *TaNAS2-A3*, *TaNAS2-B1*, *TaNAS2-B2*, *TaNAS2-D1*, *TaNAS2-D2*, and *TaNAS2-D3* homoeologues. Cultivar SY Mattis contained an additional *TaNAS* gene on chromosome 6B (*TaNAS8-B2*), which shared >95.4% CDS identity with the *TaNAS8-A1*, *TaNAS8-B1*, and *TaNAS8-D1* homoeologues. All cultivars apart from cv. Chinese Spring contained an additional *TaNAS* gene on chromosome 3B (*TaNAS5-B3*), which shared >90.6% CDS identity with the *TaNAS5-B1* and *TaNAS5-B2* homoeologues. All 425 *TaNAS* CDSs from cvs Chinese Spring, ArinaLrFor, Julius, Lancer, Norin61, SY Mattis, Mace, Jagger, Landmark, Stanley, and Fielder, and *T. spelta* L. were aligned (Supplementary Fig. S2), to assess gene and protein variation between cultivars (Supplementary Table S5). Identity analysis using PhyML in Geneious clustered each *TaNAS1*, *TaNAS2*, *TaNAS3*, *TaNAS4*, *TaNAS5*, *TaNAS6*, *TaNAS7*, *TaNAS8*, *TaNAS9*, and *TaNAS10* group together (Supplementary Fig. S2). Slight sequence variation between cultivars was observed, with up to seven haplotypes present in some homoeologues (Supplementary Table S5). The *TaNAS2-A1*, *TaNAS2-A3*, *TaNAS2-D1*, *TaNAS2-D2*, *TaNAS2-D3*, *TaNAS3-B1*, and *TaNAS6-B1* genes shared 100% CDS identity between all cultivars.

### The *TaNAS* genes encode proteins that are conserved but vary in length

**Fig. 2.**
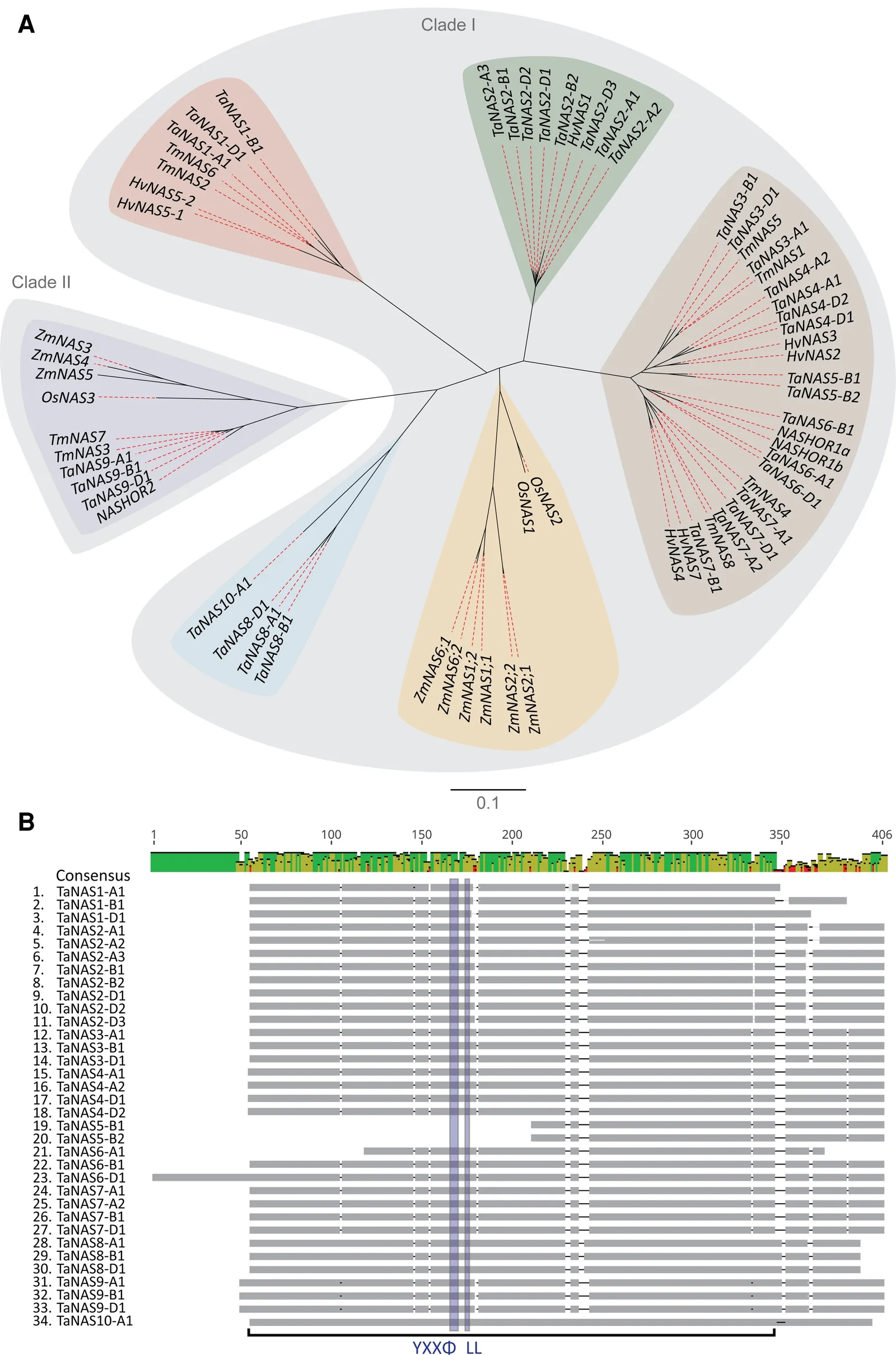
Phylogenetic analysis of *TaNAS* genes and TaNAS protein alignment. (A) Unrooted phylogenetic tree of *NAS* coding sequences in bread wheat (*Triticum aestivum* L., *Ta*), einkorn wheat (*Triticum monococcum* L., *Tm*), barley (*Hordeum vulgare* L., *Hv*), maize (*Zea mays* L., *Zm*), and rice (*Oryza sativa* L., *Os*). The scale bar represents evolutionary distance in substitutions per site. (B) Protein alignment of the 34 TaNAS proteins in cv. Chinese Spring. The top identity bar indicates 100% consensus amongst aligned sequences (green bars), medium consensus (yellow bars), and low consensus (red bars). Deletions in the majority (>50%) or minority (<50%) of sequence variations are indicated (black and grey lines, respectively). The blue transparent rectangles highlight the location of the conserved YXXФ and LL motifs and the black bracket indicates the conserved ‘core region’ within the TaNAS proteins.

Phylogenetic analysis showed that the cv. Chinese Spring *TaNAS* genes are highly conserved amongst monocots, and cluster into two clades (Fig. 2A). Clade II is comprised of *OsNAS3*, *ZmNAS3*, *ZmNAS4*, *ZmNAS5*, *TmNAS7*, *TmNAS3*, *NASHOR2*, *TaNAS9-A1*, *TaNAS9-B1*, and *TaNAS9-D1*. Clade I is comprised of the remaining *NAS* genes and clusters into five subgroups. All TaNAS proteins were predicted to align with the c3fpjA_ template of an archaeal NAS protein from *M. thermautotrophicus* with 100% confidence (Supplementary Table S2). The TaNAS proteins share a conserved region that is between 126 and 284 amino acids long (the ‘core region’), and for 19 TaNAS proteins this core region is 278 amino acids long. The average identity of the core region is 77.3% (52.5–100%). Outside the core region, the C-terminal region ranges from 3 to 57 amino acids and has an average identity of 31.4% (0–100%) (Fig. 2B). The TaNAS1 proteins have the shortest C-termini of all bread wheat TaNAS proteins (18 amino acids in length on average) (Fig. 2B). Some cultivars are predicted to have an extended N-terminus in TaNAS6-A1 (cvs SY Mattis, Mace, Norin61, Jagger, Stanley, ArinaLrFor, Landmark, Julius, and Lancer) and TaNAS6-D1 (cv. Chinese Spring and *T. spelta* L.). To determine whether a similar extended N-terminus is present in TaNAS6-D1 of cv. Fielder, we conducted RNA-seq under Fe-sufficient and -deficient conditions (Supplementary Fig. S1B). Coverage of RNA-seq data was only observed downstream of the predicted extended N-terminus translation start site and was only expressed under Fe deficiency, suggesting that the extended TaNAS6-D1 N-terminus is not translated in Fielder. Given that the IWGSC Refseq1.0 and Refseq2.1 annotations indicate that an extended N-terminus is present in TaNAS6-D1 of cv. Chinese Spring, we sourced Ribo-seq data from a separate study of developing cv. Chinese Spring wheat grain and further observed RNA-seq and ribosomal coverage only downstream of the predicted N-terminus (Supplementary Fig. S1A) (Guo *et al.*, 2023). All TaNAS proteins contain the conserved LL motif and tyrosine-based sorting motif (YXXΦ), where X is any amino acid and Φ is an amino acid with a bulky hydrophobic side chain, apart from TaNAS5-B2 and TaNAS5-B3 (Fig. 2B). Additionally, in cv. Chinese Spring, a single nucleotide polymorphism causes truncation of the TaNAS5-B1 protein, resulting in the loss of both the YXXΦ and LL motifs. The full-length TaNAS5-B1 is present in all other cultivars (Supplementary Table S5).

### The 34 *TaNAS* genes were predominately expressed in roots and regulated by environmental Fe conditions

**Fig. 3.**
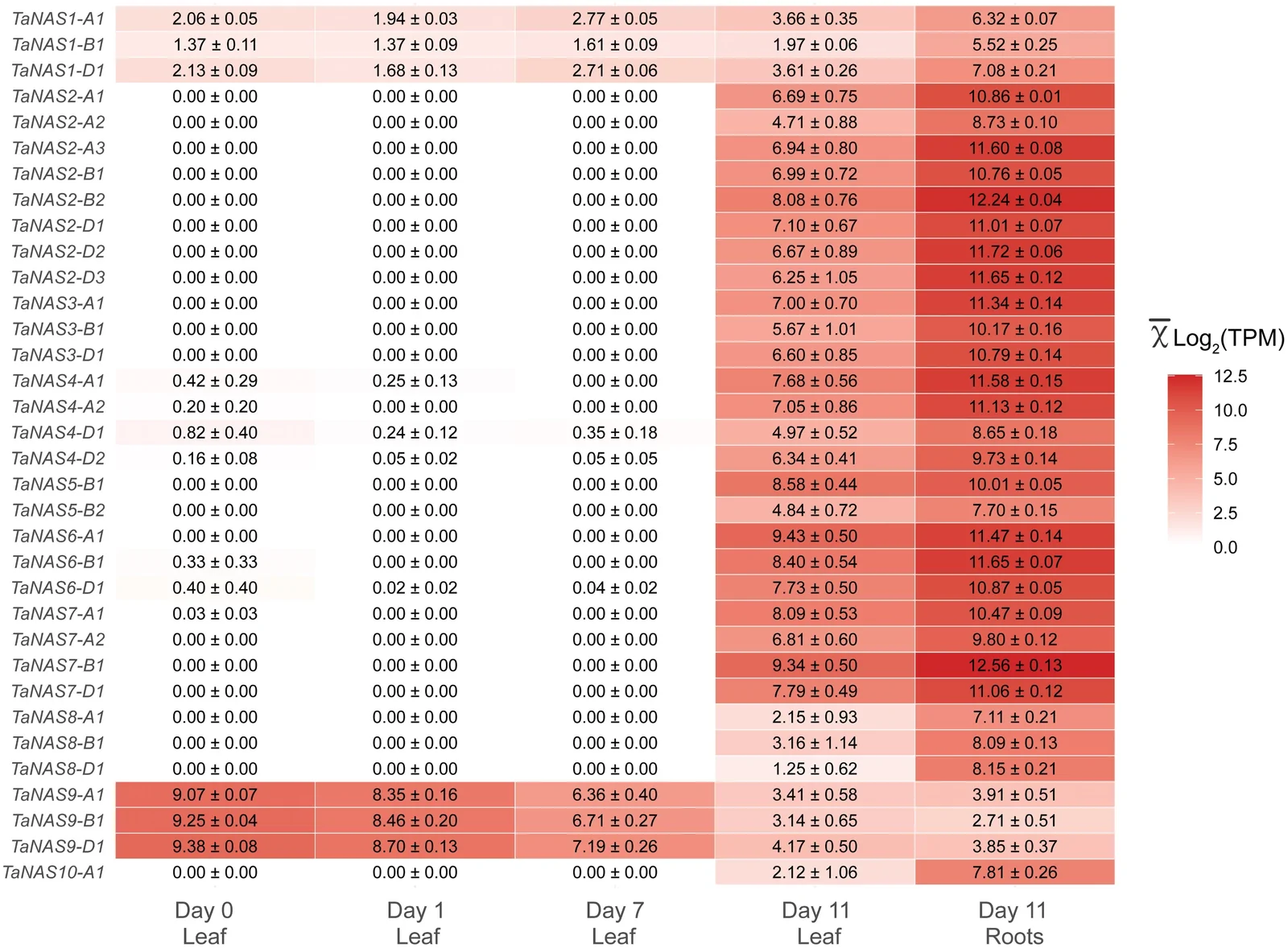
Expression of 34 *TaNAS* genes in cv. Fielder under hydroponic Fe sufficiency and deficiency. Gene expression is shown as a heatmap where white indicates low or no expression and increasing intensity of red indicates higher relative expression level. Gene expression is presented at day 0 (50 µM Fe), 1, 7, and 11 of Fe deficiency (0 µM Fe) in leaf tissue, and at day 11 of Fe deficiency in root tissue. Values represent the average of three log-transformed transcripts per million (TPM) values and the SEM for each gene (*n*=3).

RNA-seq analysis of hydroponically grown cv. Fielder plants revealed that the expression of most *TaNAS2*, *TaNAS3*, *TaNAS4*, *TaNAS5*, *TaNAS6*, *TaNAS7*, *TaNAS8*, and *TaNAS10* genes (measured in log-transformed TPM) was undetectable in leaf tissue under Fe-sufficient conditions, and strongly up-regulated by day 11 of an Fe deficiency treatment (Fig. 3). By contrast, the *TaNAS1* genes were moderately expressed in leaf tissue under Fe-sufficient conditions and up-regulated between 1.4- to 1.8-fold at day 11 of Fe deficiency. Of all the *TaNAS* genes, the *TaNAS9* homoeologues showed the highest expression in leaf tissue under Fe-sufficient conditions and the lowest expression in root tissue under Fe-deficient conditions, and the expression of these genes was down-regulated between 2.3- to 2.9-fold in the leaves at day 11 of Fe deficiency. The expression of all *TaNAS* genes was between 1.2- to 6.5-fold higher in roots compared with leaves at day 11 of Fe deficiency, excluding the *TaNAS9* genes. From RNA-seq datasets integrated into the Apollo database, most *TaNAS* genes showed high expression in wheat root tissues under Fe-sufficient conditions, apart from *TaNAS7-A2* and *TaNAS10-A1* which showed low to moderate expression in all tissues, and the *TaNAS9* genes which showed low expression in root tissues and high expression in leaf/stem tissues (Supplementary Table S2).

### Generation of six transgenic bread wheat populations with constitutive overexpression of *TaNAS* genes

**Fig. 4.**
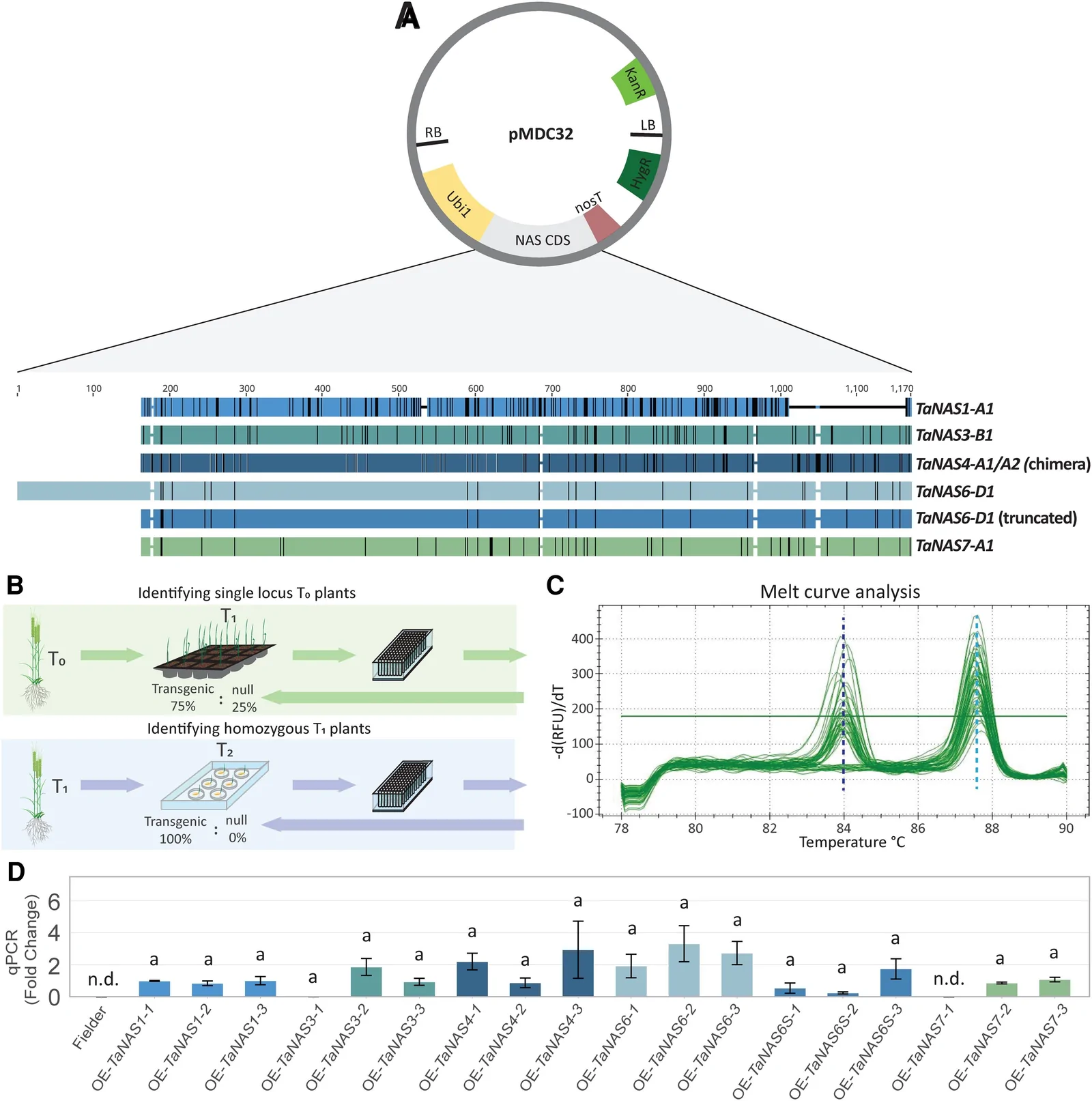
Development of six transgenic bread wheat populations with constitutive overexpression of *TaNAS* genes. (A) Schematic of the pMDC32 construct containing the right border (RB), Ubiquitin promoter 1 (Ubi-1) driving either the *TaNAS1-A1*, *TaNAS3-B1*, *TaNAS4-A1*/*A2* chimera, *TaNAS6-D1*, truncated *TaNAS6-D1*, or the *TaNAS7-A1* coding sequence, nopaline synthase terminator (nosT), hygromycin resistance (HygR), left border (LB), and kanamycin resistance (KanR). An alignment of the six *TaNAS* coding sequences with sequence variations (vertical black lines) or deletions (horizontal lines) is indicated. Nucleotide positions are indicated above the alignment. (B) Single-locus T_0_ plants were identified by screening T_1_ populations for a transgenic:null ratio of 3:1 (top panel). Homozygous T_1_ plants were identified by screening T_2_ populations for a transgenic proportion of 100% (bottom panel). (C) Both screening methods used a melt curve analysis to rapidly distinguish a null and transgenic samples. A peak at 84 °C (dark blue dashed line) indicates the presence of *HygR* (transgenic) and a peak at 87.6 °C (light blue dashed line) indicates the presence of a bread wheat housekeeping gene (*TaCyclophilin*). (D) Relative gene expression analysis of the OE-*TaNAS* transgene within 18 homozygous transgenic events. The *y*-axis represents fold change relative to OE-*TaNAS1-1*. Transgene expression was normalized against the geometric mean of three housekeeping genes (*TaACT*, *TaGAPDH*, and *TaSAR*). The error bars represent the SEM of three biological replicates (seedlings at the one-leaf stage) (*n*=3), where each biological replicate is comprised of three technical replicates. The letters indicate significant differences as determined by a one-way ANOVA (post-hoc Tukey’s HSD test). Lines where expression was not detected (n.d.) are indicated. The donor (cv. Fielder) was used as a negative control.

Six populations of bread wheat cv. Fielder plants constitutively overexpressing individual *TaNAS* genes by the maize Ubi-1 promoter were generated through *A. tumefaciens*-mediated transformation: (i) populations containing *TaNAS1-A1* constitutive overexpression constructs are hereafter referred to as OE-*TaNAS1*; (ii) populations containing *TaNAS3-B1* constitutive overexpression constructs are hereafter referred to as OE-*TaNAS3*; (iii) populations containing chimeric *TaNAS4-A1* N-terminus/*TaNAS4-A2* C-terminus constitutive overexpression constructs are hereafter referred to as OE-*TaNAS4*; (iv) populations containing *TaNAS6-D1* constitutive overexpression constructs are hereafter referred to as OE-*TaNAS6*; (v) populations containing truncated *TaNAS6-D1* constitutive overexpression constructs lacking the extended N-terminus are hereafter referred to as OE-*TaNAS6S*; and (vi) populations containing *TaNAS7-A1* constitutive overexpression constructs are hereafter referred to as OE-*TaNAS7* (Fig. 4A). Three T_1_ homozygous single-locus events (termed 1, 2, and 3) and an NS control were isolated for each *TaNAS* population using a high-throughput melt-curve approach (Fig. 4B, C) and advanced for further analysis in the glasshouse and field. The *TaNAS* transgenes were found to be expressed in all but one of the OE-*TaNAS* events and at similar expression levels (Fig. 4D). Transgene expression was not detected in OE-*TaNAS7-1*, and this event was therefore excluded from subsequent analysis.

### The OE-*TaNAS* populations displayed normal growth under glasshouse and field conditions

**Fig. 5.**
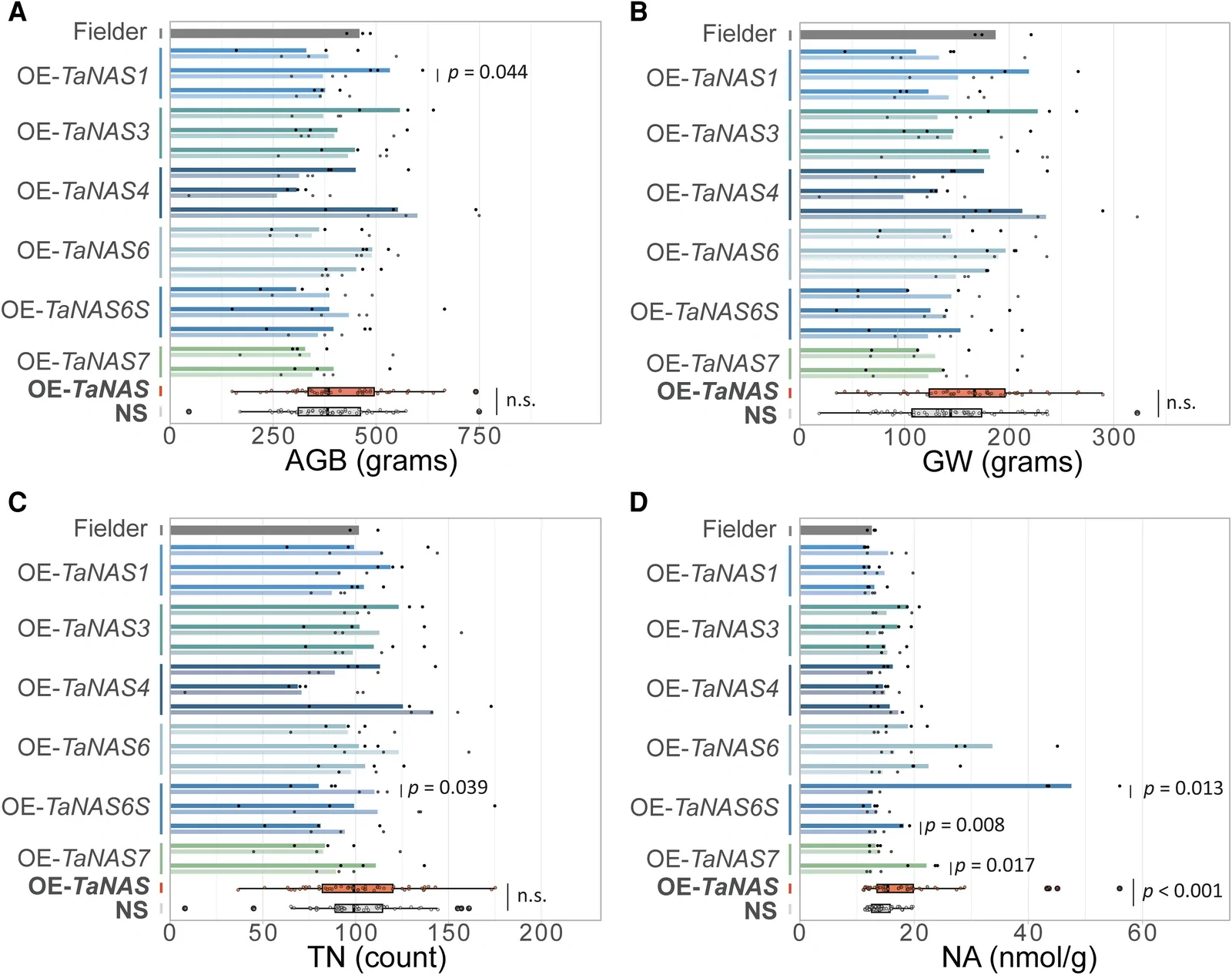
Agronomic traits and grain nicotianamine concentrations of T_4_ OE-*TaNAS* and NS plants in the field. (A) Above-ground biomass (AGB, g m^–2^), (B) grain weight (GW, g m^–2^), (C) tiller number (TN m^–2^), and (D) nicotianamine (NA) concentrations (nmol g^–1^) of OE-*TaNAS1* (blue), OE-*TaNAS3* (teal), OE-*TaNAS4* (navy blue), OE-*TaNAS6* (light blue), OE-*TaNAS6S* (dark blue), and OE-*TaNAS7* (green) lines, respective null segregants (NS; faded colour of each), and cv. Fielder (grey) in the field in 2023. The donor (cv. Fielder) is at the top (grey bar). Events 1, 2, and 3 for each OE-*TaNAS* line are shown at the top, middle, and bottom, respectively (*n*=3). Statistical differences between OE-*TaNAS* events and their respective NS are shown as *P-*values determined by a two-sample Student’s *t*-test assuming unequal variance with a 95% confidence interval. The box plots represent all OE-*TaNAS* (orange) values compared with all NS (grey) values. Non-significant (n.s.) values between all OE-*TaNAS* and all NS values as determined by a two-sample Student’s *t*-test assuming unequal variance are indicated.

At the T_1_ generation, no phenotypic differences were detected between OE-*TaNAS* plants and their respective NS (Supplementary Fig. S3A–F). To expedite grain bulking, the T_2_ generation was speed bred, and the T_3_ OE-*TaNAS* and NS plants were grown under glasshouse and field conditions in 2022. At the T_3_ in the glasshouse, above-ground biomass (AGB), tiller number, and total grain weight (TGW) were all similar between OE-*TaNAS* plants and their respective NS (Supplementary Fig. S4A–C). Exceptions include OE-*TaNAS1-2* which had 1.2-fold (*P*=0.041) higher AGB and 1.3-fold (*P*=0.011) higher TGW relative to the NS, and OE-*TaNAS3-2* which had 15% (*P*=0.028) lower AGB and 23% (*P*=0.010) lower TGW relative to the NS (Supplementary Fig. S4A, C). At the T_3_ in the field, no differences in subsampled grain weight were detected between OE-*TaNAS* plants and their respective NS (Supplementary Fig. S4D). At the T_4_ in the field, agronomic traits were similar between all OE-*TaNAS* plants and their respective NS (Fig. 5A–C). Exceptions include OE-*TaNAS1-2* which had a 1.4-fold higher AGB relative to the NS (*P*=0.044) and OE-*TaNAS6S-1* which had 28% lower tiller number relative to the NS (*P*=0.039) (Fig. 5A, C).

### Several OE-*TaNAS* populations had higher grain nicotianamine, Fe, and Zn concentrations

**Fig. 6.**
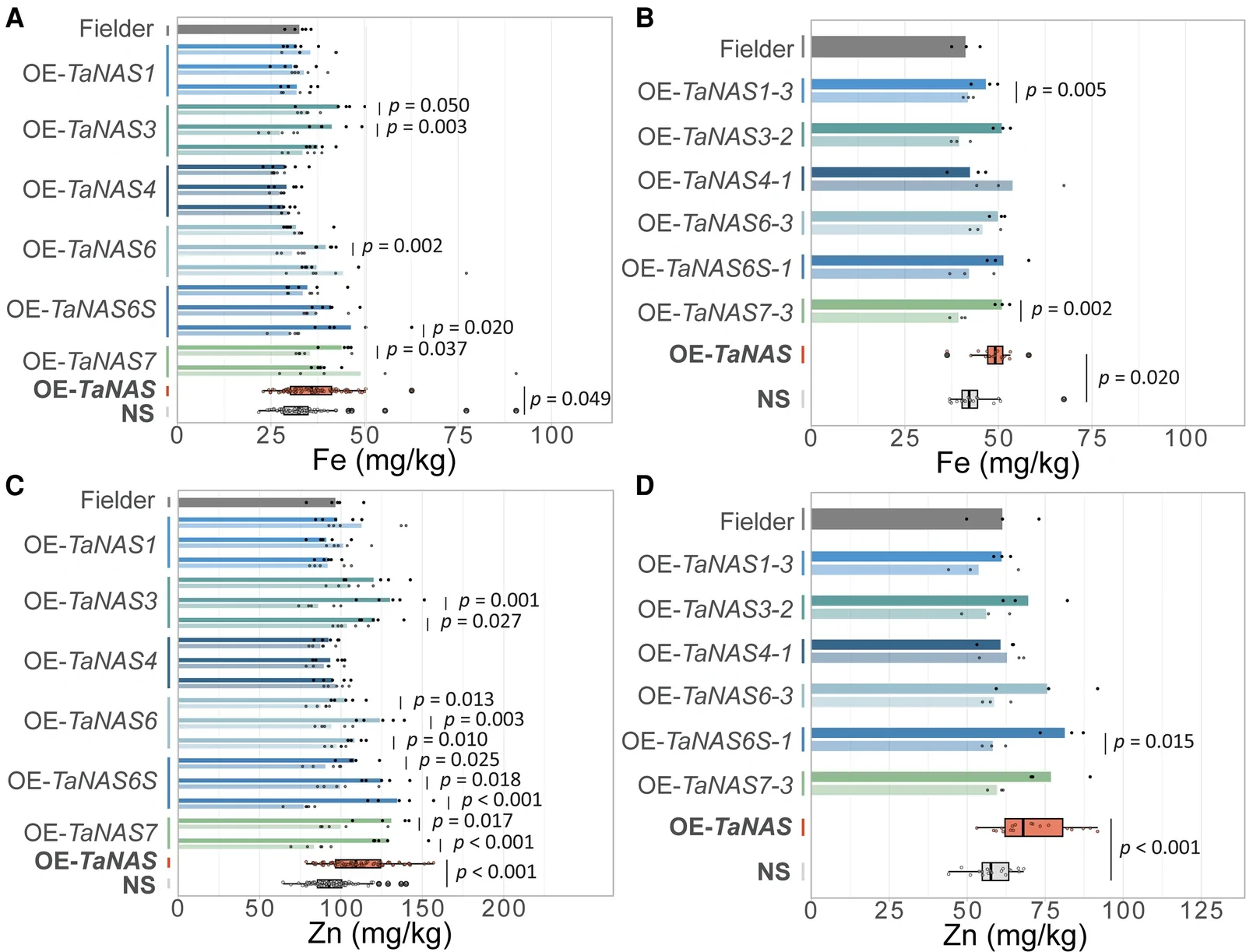
Grain Fe and Zn concentrations of T_3_ OE-*TaNAS* and NS plants in glasshouse and field experiments. Grain (A, B) Fe and (C, D) Zn concentrations of OE-*TaNAS1* (blue), OE-*TaNAS3* (teal), OE-*TaNAS4* (navy blue), OE-*TaNAS6* (light blue), OE-*TaNAS6S* (dark blue), and OE-*TaNAS7* (green) lines, respective null segregants (NS; faded colour of each), and cv. Fielder (grey) in the glasshouse and field in 2022. For the glasshouse experiment (A, C) events 1, 2, and 3 for each OE-*TaNAS* line are shown at the top, middle, and bottom, respectively (*n*=5). For the field experiment (B, D), events OE-*TaNAS1-3*, OE-*TaNAS3-2*, OE-*TaNAS4-1*, OE-*TaNAS6-3*, OE-*TaNAS6S-1*, and OE-*TaNAS7-3* are shown (*n*=3). The box plots represent all OE-*TaNAS* (orange) values compared with all NS (grey) values. Statistical differences between OE-*TaNAS* events and their respective NS are shown as *P-*values determined by a two-sample Student’s *t*-test assuming unequal variance with a 95% confidence interval.

At the T_1_ generation in the glasshouse, grain NA concentrations were >2-fold higher in OE-*TaNAS3-1* (2.7-fold), OE-*TaNAS3-2* (2.5-fold), OE-*TaNAS6-2* (2.5-fold), OE-*TaNAS6-3* (2.4-fold), OE-*TaNAS6S-1* (13.6-fold), OE-*TaNAS6S-3* (2.0-fold), OE-*TaNAS7-2* (4.2-fold), and OE-*TaNAS7-3* (2.6-fold) grain relative to their respective NS (Supplementary Fig. S3G). Notably, grain DMA concentrations were also 2.6-fold higher in OE-*TaNAS6S-1* relative to the NS (Supplementary Fig. S3H). At the T_4_ generation in the field, grain NA concentrations were 3.7- (*P*=0.013) and 1.4-fold (*P*=0.008) higher in OE-*TaNAS6S-1* and OE-*TaNAS6S-3*, respectively, relative to the NS (Fig. 5D; Supplementary Table S6). At the T_3_ in the glasshouse, grain Fe concentrations were 1.2- (*P*=0.050), 1.5- (*P*=0.003), 1.3- (*P*=0.002), 1.6- (*P*=0.020), and 1.2-fold (*P*=0.037) higher in OE-*TaNAS3-1*, OE-*TaNAS3-2*, OE-*TaNAS6-2*, OE-*TaNAS6S-3*, and OE-*TaNAS7-2*, respectively, relative to their NS (Fig. 6A; Supplementary Table S6). Under the same glasshouse conditions, grain Zn concentrations were 1.2- to 1.5-fold higher in OE-*TaNAS3,* 1.1- to 1.3-fold higher in OE-*TaNAS6*, 1.2- to 1.8-fold higher in *OE-TaNAS6S*, and 1.3- to 1.6-fold higher in OE-*TaNAS7* plants relative to the respective NS (Fig. 6C; Supplementary Table S6). At the T_3_ in the field, grain Fe concentrations were 1.3-fold (*P*=0.005) and 1.3-fold (*P*=0.002) higher in OE-*TaNAS3-2* and OE-*TaNAS7-3*, respectively, and grain Zn concentration was 1.4-fold (*P*=0.015) higher in OE-*TaNAS6S-1*, relative to the respective NS (Fig. 6B, D; Supplementary Table S6). Grain phosphorus, manganese, and copper did not differ significantly between the OE-*TaNAS* lines and their respective NS at the T_3_ generation (Supplementary Fig. S5; Supplementary Table S7).

### Transient expression of the full-length TaNAS6 enzyme increased nicotianamine biosynthesis in *Nicotiana benthamiana* leaves

**Fig. 7.**
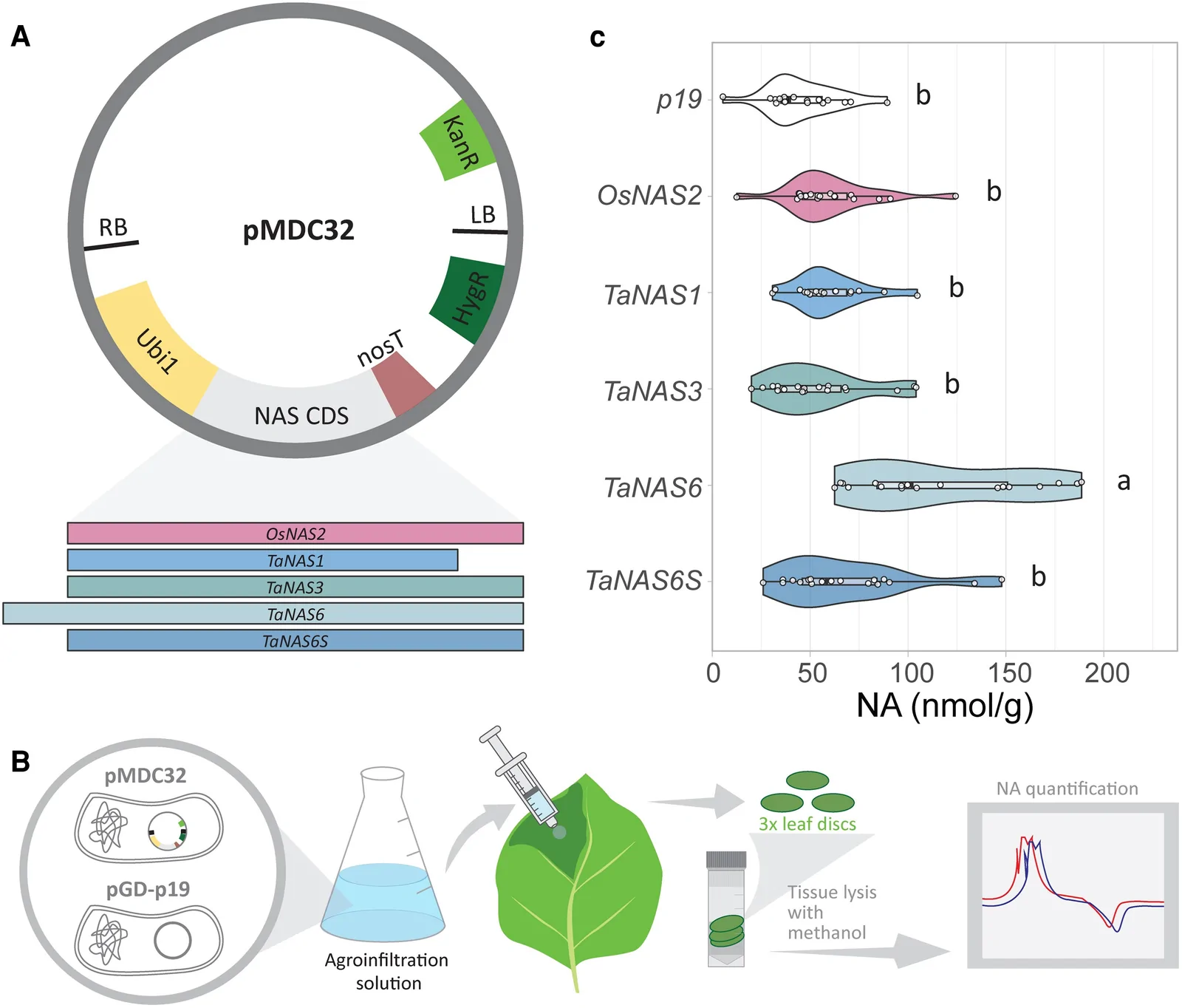
Transient expression of TaNAS enzymes in *Nicotiana benthamiana* leaves. (A) Schematic of the pMDC32 vector with different *NAS* coding sequences representing a range of NAS protein structures. (B) Each agroinfiltration solution contained *Agrobacterium tumefaciens* (GV3101) transformed with either the pMDC32-*NAS* or pGD-p19 (p19) vectors (1:1 mixture). The pGD-p19 construct reduces post-transcriptional gene silencing. Each agroinfiltration solution was infiltrated into six *N. benthamiana* leaves. Three leaf discs surrounding one infiltration site were homogenized in methanol for NA quantification. (C) NA concentrations (nmol g^–1^) for each pMDC32-*NAS* construct. The pGD-p19 (p19) construct (white) served as a negative control. The pMDC32-*OsNAS2* construct from Beasley *et al*. (2019a) was used as an additional *NAS* construct. Each data point represents three bulked leaf discs surrounding one infiltration site (*n*=18). The letters indicate significant differences as determined by a one-way ANOVA (post-hoc Tukey’s HSD test).

The activity of the TaNAS1, TaNAS3, TaNAS6, and TaNAS6S enzymes was assessed using a transient *N. benthamiana* system (Fig. 7). Transient expression of TaNAS6S, TaNAS1, TaNAS3, and a rice NAS enzyme (OsNAS2) resulted in slightly elevated NA concentrations in *N. benthamiana* leaves that did not significantly differ from endogenous leaf NA (Fig. 7C). Transient expression of the full-length TaNAS6 enzyme increased NA concentrations 2.58-fold higher (*P*=3.10×10^−10^) than endogenous leaf NA and 1.7-fold higher (*P*=1.64×10^−5^) relative to transient expression of TaNAS6S (a truncated version of TaNAS6). These results suggest that TaNAS6 is efficient at catalyzing NA biosynthesis in *N. benthamiana* leaf tissue and that the presence of the extended N-terminus is important to TaNAS6 function.

## Discussion

This analysis of the *TaNAS* gene family in bread wheat included the updated IWGSC reference sequence (Refseq2.1) and outputs of the 10+ Wheat Genome Project (Borrill *et al.*, 2016; Appels *et al.*, 2018; Ramírez-González *et al.*, 2018; Walkowiak *et al.*, 2020; Zhu *et al.*, 2021). The Apollo platform, which integrates high- and low-confidence gene annotations from IWGSC RefSeq v2.1 and NCBI, alongside RNA-seq and proteome data, provided the resource for manually annotating genes within the bread wheat genome. In Apollo, RNA-seq read alignment validated the expression of several *TaNAS* genes, resolving discrepancies in CDS annotations between IWGSC RefSeq v2.1 and NCBI for *TaNAS1-B1*, *TaNAS1-D1*, *TaNAS5-B1*, and *TaNAS6-A1* (Supplementary Tables S1, S2). In some cases, the IWGSC RefSeq v2.1 lacked a sufficient annotation, and the RNA-seq data present in Apollo corroborated the annotations from NCBI or IWGSC RefSeq v1.1 (e.g. the *TaNAS4-D1* and *TaNAS4-D2* genes). The criteria for *TaNAS* gene classification included 100% alignment with the c3fpjA_ template and a transcriptional response to Fe deficiency. Although the YXXΦ and LL motifs are generally considered important for NAS function, TaNAS5-B1 and TaNAS5-B2 in cv. Chinese Spring and TaNAS5-B2 and TaNAS5-B3 in all other cultivars do not contain these motifs (Figs 1, 2; Supplementary Table S2), as is the case for some eudicot NAS proteins (Nozoye *et al*., 2014; Seebach *et al*., 2023). We therefore included these *TaNAS5* genes in our analysis due to their 100% alignment with the c3fpjA_ template, high sequence conservation with other *TaNAS* genes, and transcriptional up-regulation under Fe deficiency (Figs 2B, 3). However, future *in planta* studies investigating the enzymatic activity of these truncated TaNAS5 proteins that lack these YXXΦ and LL motifs are required to confirm their function.

All of the TaNAS proteins contained a highly conserved ‘core’ amino acid sequence (which ranged between 126 and 284 amino acids in length), flanked by variable N- and C-terminal regions (Fig. 2B). Our *TaNAS* annotations did not identify the N-terminal extensions previously described for *TaNAS5-B1* and *TaNAS6-A1*, due to discrepancies between these original annotations (prior to publication of the first wheat genome reference), Refseq2.1, and NCBI (Bonneau *et al.*, 2016). Interestingly, the *TaNAS6-A1* annotation demonstrated inconsistent sequence length in base pairs between Refseq2.1 (which predicted a truncated protein) and NCBI (which predicted a slightly extended N-terminus without transcriptional evidence), so the longest Open Reading Frame that contained the YXXΦ and LL motifs, and had RNA-seq evidence of transcription, was annotated. Both Refseq2.1 and NCBI predicted the 162 bp extended N-terminus in *TaNAS6-D1*. However, we were unable to find evidence for transcription or translation of the extended N-terminus in cv. Fielder plants under Fe deficiency or in cv. Chinese Spring developing grain under control conditions (Supplementary Fig. S1) (Guo *et al.*, 2023). We constitutively overexpressed the *TaNAS6-D1* gene with and without the extended N-terminal region and identified one OE-*TaNAS6S* event (without the extended N-terminal region) that showed the greatest fold increase in grain NA (3.7-fold), Zn (1.4-fold), and Fe (1.2-fold) concentrations relative to the NS under field conditions (Figs 5D, 6B, D; Supplementary Table S6). Further studies are warranted to determine whether these differences in the OE-*TaNAS6S-1* event translate into changes in grain mineral localization and/or Fe and Zn bioavailability. Other OE-*TaNAS6* and OE-*TaNAS6S* events consistently showed elevated grain NA, Zn, and Fe concentrations, albeit to different degrees (Figs 5D, 6; Supplementary Fig. S3G, I, J; Supplementary Table S6). Interestingly, transient expression of the *TaNAS6-D1* gene in *N. benthamiana* resulted in 1.7-fold higher leaf NA concentrations relative to transient expression of a truncated *TaNAS6-D1* gene lacking the extended N-terminal region (Fig. 7), suggesting that the extended N-terminus plays a functional role in NA biosynthesis. However, it is likely that this role is constrained to vegetative tissue, given that the expression of this extended N-terminus is not detected in the developing grain (Supplementary Fig. S1A).

While the extended N-termini identified in the original characterization of TaNAS proteins were predicted to determine subcellular localization, a separate study in maize concluded that extended N-termini do not influence ZmNAS protein localization (Zhou *et al.*, 2013; Bonneau *et al.*, 2016; Emanuelsson *et al.*, 2000). Ultimately, additional high quality bread wheat ribosomal sequencing or proteomic datasets across multiple environments and developmental stages will be necessary to confirm whether any of the TaNAS proteins (including TaNAS6-D1) contain a functional extended N-terminus (Guo *et al.*, 2023). The TaNAS1 proteins contain truncated C-terminal regions relative to other TaNAS proteins (Fig. 2B), and truncation of the C-terminus in the Arabidopsis AtNAS1 protein increases enzymatic activity due to loss of an autoinhibitory motif (TRGCMFMPCNCS) (Seebach *et al.*, 2023). Although the same motif is poorly conserved among the TaNAS proteins, other monocot-specific C-terminal autoinhibitory motifs may be present and, given their truncated C-terminal regions, the TaNAS1 proteins may be missing these putative autoinhibitory motifs (Figs 2B, 4A). Constitutively overexpressing the *TaNAS1-A1* gene (the shortest of the *TaNAS1* homoeologues) did not influence grain Fe, Zn, or NA concentrations in all three OE-*TaNAS1* events, suggesting that the TaNAS1-A1 protein may not have increased enzymatic activity due to the absence of a C-terminal autoinhibitory motif (Figs 5D, 6A, B). Interestingly, the OE-*TaNAS1-2* event had higher AGB and grain weight in the 2022 glasshouse season (2.60-fold, *P*=0.041 for AGB and 1.33-fold, *P*=0.011 for grain weight) and 2023 field season (1.43-fold, *P*=0.044 for AGB and 1.44-fold, *P*=0.112 for grain weight) relative to the NS (Fig. 5A; Supplementary Fig. S4A, C). Whether these changes in OE-*TaNAS1-2* agronomic traits were due to an unexamined effect of *TaNAS1-A1* constitutive overexpression on plant growth (e.g. elevated NA concentrations in OE-*TaNAS1-2* vegetative tissues) or integration of the *TaNAS1* transgene into a genomic location that regulates plant growth is unclear. Further investigation into the determinants of OE-*TaNAS1* agromorphology will occur alongside functional enzymatic assays to investigate the presence of autoinhibitory motifs in TaNAS C-termini. The variation in grain Fe, Zn, and NA concentrations among independent events of the same construct (e.g. OE-*TaNAS6S-1*, OE-*TaNAS6S-2*, and OE-*TaNAS6S-3*) cannot be explained by transgene expression levels in young shoots (Figs 4D, 5D, 6; Supplementary Fig. S3G, I, J). This finding could be instead due to changes in transgene expression occurring between seedling and grain-filling stages. For example, characterization of both transgene expression at grain filling and site-specific transgene integration may explain the high grain NA concentrations observed in the OE-*TaNAS6S-1* event relative to other OE-*TaNAS6* events (Fig. 5D; Supplementary Fig. S3G).

Conventional breeding strategies to increase grain Fe concentrations in wheat are limited by a lack of natural genetic variation in modern breeding populations (Borrill *et al.*, 2014; Ludwig and Slamet-Loedin, 2019; Saini *et al.*, 2020). Landraces and synthetic varieties display greater variation in grain Fe concentrations but often have lower yield relative to elite cultivars (Heidari *et al.*, 2024). Enhancing grain NA concentration via increased expression of *NAS* genes has long been the most promising single-gene strategy for Fe and Zn biofortification of rice and wheat (Inoue *et al.*, 2003; Wirth *et al.*, 2009; Zheng *et al.*, 2010; Johnson *et al.*, 2011; Lee *et al.*, 2012; Eagling *et al.*, 2014; Singh *et al.*, 2017; Beasley *et al.*, 2019a, b, 2022). Constitutive expression of the rice *OsNAS2* gene in bread wheat results in consistently higher NA concentrations and Fe bioavailability in white wheat flour relative to NS controls, with no associated agronomic penalty (Beasley *et al.*, 2019a, 2022). While current evidence indicates that *OsNAS2* constitutive overexpression in rice does not increase grain cadmium (Cd) concentration under normal and Cd-contaminated growth conditions (Trijatmiko *et al.*, 2016), the recent observation of elevated seed Cd concentrations in *NAS* constitutively overexpressing *A. thaliana* grown on Cd-contaminated soil highlights a need for thorough assessment of heavy metal accumulation in *NAS*-engineered rice and wheat in future studies (Hollmann *et al.*, 2025). Here we conducted three years of glasshouse and field trials and demonstrated that constitutively overexpressing *TaNAS* genes also has no negative effects on wheat agronomic performance—a key factor in grower acceptance and adoption (Fig. 5; Supplementary Figs S3, S4). Furthermore, intragenic strategies (which utilize promoters, genes, terminators, and selectable markers that are entirely from sexually compatible species) are expected to face fewer regulatory challenges than conventional transgenic methods (Espinoza *et al.*, 2013; Holme *et al.*, 2013). The results described in this study, along with the updated repository of *TaNAS* genes present in multiple wheat genomes, provide breeders with a wealth of conventional, precision and intragenic resources to enhance bread wheat nutrition.

## Supplementary Material

erag242_Supplementary_Data

## Data Availability

Raw data are included in the supplementary data within this manuscript, and RNA-seq data are available from NCBI-GEO (GEO accession no. GSE283676).

## Supplementary data

The following supplementary data are available at *JXB* online.

Fig. S1. Ribosomal profile and RNA-seq coverage of *TaNAS6-D1*.

Fig. S2. Alignment of 425 *TaNAS* coding sequences from cvs Chinese Spring, ArinaLrFor, Julius, Lancer, Norin61, SY Mattis, Mace, Jagger, Landmark, Stanley, and Fielder, and *Triticum spelta* L.

Fig. S3. Phenotypic and nutritional analysis of T_1_ OE-*TaNAS* and NS plants in the glasshouse.

Fig. S4. Agronomic traits of T_3_ OE-*TaNAS* and NS plants in the glasshouse and field.

Fig. S5. Grain Mn, Cu, and P concentrations of T_3_ OE-*TaNAS* and NS plants in glasshouse and field experiments.

Table S1. Gene models and chromosomal location of 34 *TaNAS* genes in bread wheat cv. Chinese Spring and the additional nine potential *TaNAS* genes that were excluded from this study.

Table S2. Expression patterns and protein features of 34 *TaNAS* genes in bread wheat cv. Chinese Spring and the additional nine potential *TaNAS* genes that were excluded from this study.

Table S3. Gene IDs and coding sequences used as queries to blast for novel bread wheat *NAS* genes.

Table S4. Primer sequences used in OE-*TaNAS* transgene characterization.

Table S5. Haplotypes of *TaNAS* genes in 11 different bread wheat cultivars and *Triticum spelta* L.

Table S6. Grain Fe, Zn, and NA fold differences between OE-*TaNAS* and NS plants at the T_3_ and T_4_ generations.

Table S7. Grain P, Mn, and Cu fold differences between OE-*TaNAS* and NS plants at the T_3_ generation.
