# Supplementary material for: Analysis of the TaNAS gene family in bread wheat identifies additional members and candidates for intragenic biofortification
Source: J Exp Bot. 2026 May 22;77(16):5108–23. doi: 10.1093/jxb/erag242 (PMC13529340; doi:10.1093/jxb/erag242)
Supplement: Supplementary file 1 [file erag242_supplementary_data.pdf]

Supplementary Table S1. Gene models and chromosomal location of 34 *TaNAS* genes in bread wheat cv. Chinese Spring and the additional 9 potential *TaNAS* genes that were excluded from this study. Gene models (IWGSC RefSeq1.1, 2.1, and NCBI) of the included *TaNAS* genes (rows 1-34) and excluded *TaNAS* genes (rows 35-43), and their chromosomal location (IWGSC RefSeq2.1) are described.

No	New annotation	IWGSC RefSeqv1.1	IWGSC RefSeqv2.1	NCBI annotation	Chromosomal Location	CDS annotation used
1	***TaNAS1-A1***	TraesCS2A02G033500	TraesCS2A03G066300	LOC123187039	Chr2A:18046576-18048604 -	Refseq2.1
2	***TaNAS1-B1***	TraesCS2B02G047100	TraesCS2B03G098000	LOC123043227	Chr2B:26588743-26592485 -	Refseq2.1
3	***TaNAS1-D1***	TraesCS2D02G033000	TraesCS2D03G061000	LOC123051093	Chr2D:13092629-13096137 -	Refseq2.1
4	***TaNAS2-A1***	TraesCS6A02G163100	TraesCS6A03G395600	LOC123130501	Chr6A:160986866-160988156 -	Refseq2.1
5	***TaNAS2-A2***	TraesCS6A02G163200	TraesCS6A03G396300	LOC123130503	Chr6A:161119099-161125376 -	Refseq2.1
6	***TaNAS2-A3***	TraesCS6A02G165100	TraesCS6A03G404500	LOC123130517	Chr6A:165538805-165539794 -	Refseq2.1
7	***TaNAS2-B1***	TraesCS6B02G187400	TraesCS6B03G477500	LOC123133995	Chr6B:219612150-219613139 -	Refseq2.1
8	***TaNAS2-B2***	TraesCS6B02G186100	TraesCS6B03G474000	LOC123133989	Chr6B:218289819-218290808 -	Refseq2.1
9	***TaNAS2-D1***	TraesCS6D02G148200	TraesCS6D03G330700	LOC123141358	Chr6D:142768539-142771342 +	Refseq2.1
10	***TaNAS2-D2***	TraesCS6D02G148600	TraesCS6D03G331700	LOC123144159	Chr6D:143120609-143121939 -	Refseq2.1
11	***TaNAS2-D3***	TraesCS6D02G148000	TraesCS6D03G330000	LOC123141357	Chr6D:142582221-142583640 +	Refseq2.1
12	***TaNAS3-A1***	TraesCS2A02G049900	TraesCS2A03G093200	LOC123184397	Chr2A:22269439-22270719 -	Refseq2.1
13	***TaNAS3-B1***	TraesCS2B02G060800	TraesCS2B03G130700	LOC123040191	Chr2B:32997616-32998896 -	Refseq2.1
14	***TaNAS3-D1***	TraesCS2D02G049200	TraesCS2D03G092200	LOC123048357	Chr2D:18625470-18626900 -	Refseq2.1
15	***TaNAS4-A1***	TraesCS5A02G552400	TraesCS5A03G1289600	LOC123106177	Chr5A:708105606-708106934 -	Refseq2.1
16	***TaNAS4-A2***	TraesCS5A02G552000	TraesCS5A03G1289100	LOC123106176	Chr5A:708052165-708053166 -	Refseq2.1

17	***TaNAS4-D1***	TraesCSU02G125200	n/a	LOC12309921<br>6	Chr4D:513233447-<br>513234914 -	Refseq1.1
18	***TaNAS4-D2***	TraesCSU02G125500	n/a	LOC12309921<br>4	Chr4D:513215952-<br>513217368 +	Refseq1.1
19	***TaNAS5-B1***	TraesCS3B02G068500	TraesCS3B03G154800	LOC12306466<br>1	Chr3B:50315898-<br>50316948 +	Refseq2.1
20	***TaNAS5-B2***	TraesCS3B02G068400	TraesCS3B03G154700	n/a	Chr3B:50077736-<br>50078278 +	Refseq2.1
21	***TaNAS6-A1***	TraesCS4A02G127900LC	TraesCS4A03G249100LC	LOC12308376<br>6	Chr4A:148780629-<br>148781781 +	Manual<br>annotation
22	***TaNAS6-B1***	TraesCS4B02G183900	TraesCS4B03G520200	LOC12309488<br>5	Chr4B:402432887-<br>402433879 -	Refseq2.1
23	***TaNAS6-D1***	TraesCS4D02G184900	TraesCS4D03G458900	LOC12309767<br>8	Chr4D:323251547-<br>323253910 -	Refseq2.1
24	***TaNAS7-A1***	TraesCS6A02G093000	TraesCS6A03G214000	LOC12313029<br>2	Chr6A:63817836-<br>63819203 -	Refseq2.1
25	***TaNAS7-A2***	TraesCS6A02G386200	TraesCS6A03G973000	LOC12313132<br>5	Chr6A:606021899-<br>606022891 +	Refseq2.1
26	***TaNAS7-B1***	TraesCS6B02G425200	TraesCS6B03G1190400	LOC12313501<br>2	Chr6B:703619814-<br>703620806 +	Refseq2.1
27	***TaNAS7-D1***	TraesCS6D02G370800	TraesCS6D03G854100	LOC12314611<br>7	Chr6D:477989584-<br>477990867 +	Refseq2.1
28	***TaNAS8-A1***	TraesCS6A02G398700	TraesCS6A03G1000600	LOC12313137<br>1	Chr6A:612027263-<br>612028597 -	Refseq2.1
29	***TaNAS8-B1***	TraesCS6B02G438900	TraesCS6B03G1227100	LOC12313509<br>9	Chr6B:714051809-<br>714053050 -	Refseq2.1
30	***TaNAS8-D1***	TraesCS6D02G382900	TraesCS6D03G881800	LOC12314218<br>2	Chr6D:483833945-<br>483835250 +	Refseq2.1
31	***TaNAS9-A1***	TraesCS2A02G095700	TraesCS2A03G196300	LOC12318740<br>3	Chr2A:53939313-<br>53940335 +	Refseq2.1
32	***TaNAS9-B1***	TraesCS2B02G111100	TraesCS2B03G264600	LOC12304359<br>8	Chr2B:80456493-<br>80458103 +	Refseq2.1
33	***TaNAS9-D1***	TraesCS2D02G094200	TraesCS2D03G196500	LOC12305146<br>5	Chr2D:48272558-<br>48273580 +	Refseq2.1
34	***TaNAS10-A1***	TraesCS3A02G468300	TraesCS3A03G1088500	LOC12305924<br>4	Chr3A:700464391-<br>700465811 +	Refseq2.1
35		TraesCS2A02G031500LC	TraesCS2A03G065900LC		Chr2A:18030402-<br>18030782 -	Refseq2.1

36	n/a	n/a	n/a	Chr3B:50111254-50113113 +	n/a
37	TraesCS3D02G055800	TraesCS3D03G103600	n/a	Chr3D:23087305-23088112 +	Refseq2.1
38	TraesCS4A02G128000LC	TraesCS4A03G249200LC	n/a	Chr4A:148782303-148782891 +	Refseq2.1
39	TraesCS4B02G599900LC	TraesCS4B03G989100LC	n/a	Chr4B:665502039-665503016 +	Nontranslating CDS
40	TraesCS4B02G600600LC	TraesCS4B03G989900LC	n/a	Chr4B:665533725-665543791 -	Nontranslating CDS
41	TraesCSU02G125100	n/a	n/a	Chr4D:513253441-513254120 -	Refseq1.1
42	TraesCS5A02G733600LC	TraesCS5A03G1288900LC	n/a	Chr5A:708031745-708032418 +	Refseq2.1
43	TraesCS7B02G813200LC	TraesCS7B03G1308900LC	n/a	Chr7B:755163999-755164979 +	n/a

Supplementary Table S2. Expression patterns and protein features of 34 *TaNAS* genes in bread wheat cv. Chinese Spring and the additional 9 potential *TaNAS* genes that were excluded from this study. Gene expression of root, leaf, stem, spike or grains at all time points (INRA RNA-seq viewed in Apollo) are categorised with 0-5 (n/a), 5-100 (Low), 100-500 (Med), and greater than 500 (High) maximum RNA-seq reads. Upregulation or down regulation of a *TaNAS* gene in response to Fe deficiency was indicated with a 'Yes', and no change in expression was indicated with a 'No', as determined by RNA-seq data generated in this study. Protein features including the alignment of a TaNAS protein to the c3fpjA_ template with 100% confidence is indicated with a 'Yes', and lower confidence or no alignment to the c3fpjA_ template is indicated. Protein features where genes could not be annotated with confidence are indicated with 'n/a'.

No	New annotation	Root expression (Apollo)	Leaf expression (Apollo)	Stem expression (Apollo)	Spike expression (Apollo)	Grain expression (Apollo)	Response to Fe deficiency	c3fpjA_ protein	YXXΦ	LL	Length
1	***TaNAS1-A1***	High	Low	Med	Med	Low	Yes	Yes	Yes	Yes	843
2	***TaNAS1-B1***	High	Low	Low	Low	Low	Yes	Yes	Yes	Yes	933
3	***TaNAS1-D1***	High	Med	Med	Med	Med	Yes	Yes	Yes	Yes	894
4	***TaNAS2-A1***	High	n/a	n/a	Low	Low	Yes	Yes	Yes	Yes	981
5	***TaNAS2-A2***	High	n/a	n/a	n/a	n/a	Yes	Yes	Yes	Yes	951
6	***TaNAS2-A3***	High	n/a	n/a	n/a	n/a	Yes	Yes	Yes	Yes	990
7	***TaNAS2-B1***	High	n/a	n/a	n/a	n/a	Yes	Yes	Yes	Yes	990
8	***TaNAS2-B2***	High	n/a	n/a	low	n/a	Yes	Yes	Yes	Yes	990
9	***TaNAS2-D1***	High	n/a	n/a	n/a	n/a	Yes	Yes	Yes	Yes	990
10	***TaNAS2-D2***	High	n/a	n/a	n/a	n/a	Yes	Yes	Yes	Yes	990
11	***TaNAS2-D3***	High	n/a	n/a	n/a	n/a	Yes	Yes	Yes	Yes	990
12	***TaNAS3-A1***	High	n/a	n/a	n/a	n/a	Yes	Yes	Yes	Yes	993
13	***TaNAS3-B1***	High	n/a	n/a	Low	n/a	Yes	Yes	Yes	Yes	993
14	***TaNAS3-D1***	High	n/a	n/a	Low	n/a	Yes	Yes	Yes	Yes	993
15	***TaNAS4-A1***	High	n/a	n/a	Low	n/a	Yes	Yes	Yes	Yes	1002
16	***TaNAS4-A2***	High	n/a	n/a	n/a	n/a	Yes	Yes	Yes	Yes	1002
17	***TaNAS4-D1***	High	Low	Low	Low	n/a	Yes	Yes	Yes	Yes	1002

18	***TaNAS4-D2***	High	Low	Low	Low	n/a	Yes	Yes	Yes	Yes	1002
19	***TaNAS5-B1***	High	n/a	Low	Low	n/a	Yes	Yes	No	No	543
20	***TaNAS5-B2***	High	n/a	n/a	n/a	n/a	Yes	Yes	No	No	543
21	***TaNAS6-A1***	High	n/a	n/a	n/a	Low	Yes	Yes	Yes	Yes	711
22	***TaNAS6-B1***	High	n/a	n/a	n/a	Med	Yes	Yes	Yes	Yes	993
23	***TaNAS6-D1***	High	Low	Low	Low	Med	Yes	Yes	Yes	Yes	1155
24	***TaNAS7-A1***	High	n/a	n/a	n/a	n/a	Yes	Yes	Yes	Yes	993
25	***TaNAS7-A2***	Low	n/a	n/a	Low	n/a	Yes	Yes	Yes	Yes	993
26	***TaNAS7-B1***	High	n/a	n/a	n/a	n/a	Yes	Yes	Yes	Yes	993
27	***TaNAS7-D1***	High	n/a	n/a	n/a	n/a	Yes	Yes	Yes	Yes	993
28	***TaNAS8-A1***	High	n/a	n/a	n/a	n/a	Yes	Yes	Yes	Yes	987
29	***TaNAS8-B1***	High	n/a	n/a	n/a	n/a	Yes	Yes	Yes	Yes	987
30	***TaNAS8-D1***	High	n/a	n/a	n/a	n/a	Yes	Yes	Yes	Yes	987
31	***TaNAS9-A1***	Med	High	High	Low	Med	Yes	Yes	Yes	Yes	1023
32	***TaNAS9-B1***	Med	High	High	Med	Med	Yes	Yes	Yes	Yes	1023
33	***TaNAS9-D1***	Low	High	High	Med	Med	Yes	Yes	Yes	Yes	1023
34	***TaNAS10-A1***	Med	n/a	n/a	n/a	n/a	Yes	Yes	Yes	Yes	1023
35		n/a	n/a	n/a	n/a	n/a	No	No	No	No	381
36		n/a	n/a	n/a	n/a	n/a	No	n/a	n/a	n/a	n/a
37		Low	n/a	n/a	n/a	n/a	Low	99.9	No	No	648
38		Low	n/a	n/a	n/a	n/a	Yes	No	No	No	177
39		n/a	n/a	n/a	n/a	n/a	No	n/a	n/a	n/a	n/a
40		n/a	n/a	n/a	n/a	n/a	No	No	n/a	n/a	n/a
41		Med	n/a	n/a	n/a	n/a	Yes	No	No	No	315
42		n/a	n/a	n/a	n/a	n/a	No	99.9	Yes	Yes	498
43		n/a	n/a	n/a	n/a	n/a	No	n/a	n/a	n/a	n/a

Supplementary Table S3. Gene IDs and coding sequences used as queries to blast for novel bread wheat *NAS* genes.

NAS gene ID	Coding sequence
AT5G04950	ATGGCTTGCCAAAACAATCTCGTTGTGAAGCAAATCATCGACTTGTACGACCAAATCTCAAAGCTCAAGAGCTTAAAACCTTCCAAAAATG<br>TCGACACTTTGTTCGGACAACTCGTGTCCACGTGCTTACCCACGGATACAAACATCGATGTCACAAATATGTGTGAAGAAGTCAAAGACA<br>TGAGAGCTAATCTCATCAAGCTTTGTGGTGAAGCCGAAGGTTATTTAGAGCAACACTTCTCCACAATTTTGGGATCTTTACAAGAAGACCA<br>AAACCCACTTGACCATTTACACATCTTTCCTTACTACTCCAACTACCTCAAGCTAGGCAAGCTCGAGTTCGATCTCCTGAGCCAACACTCA<br>AGCCATGTCCCCACCAAGATTGCCTTCGTGGGTTCGGGTCCGATGCCTCTCACATCCATCGTATTGGCCAAGTTTCACCTCCCCAACAC<br>GACGTTCCACAACTTTGACATCGACTCACACGCAAACACACTCGCTTCAAACCTCGTCTCTCGCGACCCGGACCTCTCAAAACGCATGA<br>TCTTCCACACAACGGACGTACTAAACGCAACCGAAGGCCTTGACCAATATGACGTCGTTTTCTTAGCGGCGCTTGTAGGGATGGACAAA<br>GAGTCAAAGGTCAAAGCCATCGAGCACTTGGAGAAACACATGGCTCCTGGAGCTGTTCTTATGCTAAGGAGTGCTCATGCTCTCAGAGC<br>TTTCTTATATCCAATCGTTGACTCGTCTGATCTCAAAGGCTTTCAACTCTTGACCATCTATCATCCAACCGATGACGTGGTTAACTCGGTTG<br>TGATCGCACGTAAGCTCGGTGGTCCGACCACGCCCGGGGTTAATGGTACTCGTGGATGCATGTTTATGCCTTGTAACTGCTCCAAGATT<br>CACGCGATCATGAACAACCGTGGTAAGAAGAATATGATCGAGGAGTTTAGTGCCATCGAGTAA
AT5G56080	ATGGCTTGCGAAAACAACCTCGTTGTGAAGCAAATCATGGACTTATACAACCAAATCTCAAACCTCGAGAGCTTAAAACCATCCAAGAAT<br>GTCGACACTTTGTTCAGACAACTTGTGTCCACGTGCTTACCAACGGACACGAACATCGATGTCACAGAGATACACGATGAAAAAGTCAA<br>AGACATGAGATCTCATCTCATCAAGCTTTGTGGTGAAGCCGAAGGTTATTTAGAGCAACACTTTTCAGCAATCTTAGGCTCTTTTGAAGAC<br>AACCCTCTAAACCATTTACACATCTTCCCCTATTACAACAACTATCTCAAACTAGGCAAACTCGAATTCGATCTCCTATCTCAACACACAAC<br>CCATGTCCCGACCAAAGTCGCCTTTATTGGTTCCGGTCCGATGCCACTTACTTCCATCGTCTTGGCCAAGTTCCACCTCCCCAACACAA<br>CGTTCCACAACTTCGACATCGACTCACACGCCAACACACTCGCTTCAAACCTCGTTTCTCGTGATTCTGACCTTTCCAAACGCATGATTT<br>TCCACACAACTGATGTATTAAACGCTAAGGAGGGGTTAGACCAATACGATGTTGTTTTCTTGGCAGCTCTTGTTGGGATGGATAAAGAGT<br>CAAAGGTCAAAGCTATTGAGCATTTAGAGAAGCATATGGCTCCTGGAGCTGTGGTGATGCTAAGAAGTGCTCATGGTCTTAGAGCTTTCT<br>TGTATCCAATCGTTGACTCTTGTGATCTTAAAGGGTTTGAGGTGTTAACCATTTATCATCCGTCTGACGACGTGGTTAATTCGGTGGTCAT<br>CGCACGTAAGCTTGGTGGTTCAAATGGAGCTCGAGGCAGCCAGATCGGACGGTGTGTGGTTATGCCTTGTAATTGCTCTAAGGTCCAC<br>GCGATCTTGAACAATCGTGGTATGGAGAAGAATTTGATCGAGGAGTATAGTGCCATCGAGTAA
AT1G09240	ATGGGTTGCCAAGACGAACAATTGGTGCAAACAATATGCGATCTCTACGAAAAGATCTCAAAGCTTGAGAGTCTAAAACCATCCGAAGAT<br>GTCAACATTCTCTTCAAGCAGCTCGTTTCCACATGCATACCACCAAACCCTAACATCGATGTCACCAAGATGTGTGACAGAGTCCAAGAG<br>ATTCGACTTAATCTCATCAAGATTTGTGGTCTAGCCGAAGGTCACTTAGAAAACCATTTCTCTTCGATCTTGACCTCTTACCAAGACAACC<br>CACTTCATCATTTAAACATTTTCCCTTATTACAACAACTATTTGAAACTCGGAAAGCTCGAGTTCGACCTCCTCGAACAAAACCTAAATGGC<br>TTTGTCCCAAAGAGTGTGGCTTTCATTGGATCTGGTCCTCTTCCTCTCACTTCCATCGTTCTTGCTTCATTCCATCTCAAAGACACAATCT<br>TTCACAACTTTGACATCGACCCATCAGCGAACTCACTCGCTTCTCTTCTGGTTTCCTCTGATCCAGACATCTCTCAACGCATGTTCTTCCA<br>CACCGTTGATATAATGGACGTGACAGAGAGCTTAAAGAGCTTTGATGTCGTGTTTCTAGCTGCTCTTGTTGGAATGAACAAAGAGGAGAA<br>AGTTAAAGTGATCGAGCATCTGCAGAAACACATGGCTCCTGGTGCTGTGCTCATGCTTAGGAGTGCTCATGGTCCGAGAGCGTTTCTTT

	ATCCGATCGTTGAGCCGTGTGATCTTCAGGGGTTCGAGGTTTTGTCTATTTATCACCCAACAGATGATGTTATCAACTCCGTGGTGATCTC<br>TAAAAAGCATCCAGTTGTTTCAATTGGGAATGTTGGTGGTCCTAATTCATGCTTGCTCAAGCCTTGCAACTGTTCCAAGACCCACGCGAA<br>AATGAACAAGAACATGATGATCGAGGAGTTCGGAGCTAGGGAGGAACAGTTGTCTTAA
AT1G56430	ATGGGTTATTGCCAAGACGACCAACTCGTAAACAAGATCTGCGATCTTTACGAAAAGATCTCGAAGCTTGAGACCCTAAAGCCTTGTGAA<br>GATGTCGACACTCTCTTCAAGCAGCTCGTGTCCACATGCATACCACCAAACCCTAACATCGACGTCACCAAGATGTCTGAAAACATCCAA<br>GAGATGAGATCAAACCTCATCAAAATCTGTGGTGAGGCTGAAGGTTACTTAGAGCATCACTTCTCTTCAATCTTAACCTCTTTTGAAGATA<br>ACCCCCTTCATCATTTGAATCTTTTTCCTTACTACAACAACTACCTCAAACTAAGCAAGCTCGAGTTTGATCTCCTCGAACAGAACCTAAA<br>CGGTTTTGTTCCAAGGACTGTAGCTTTCATTGGCTCTGGTCCTCTCCCTCTTACTTCCGTCGTTCTTGCCTCTTCCCATCTCAAAGACTC<br>GATCTTTCATAACTTTGACATCGACCCATCAGCGAATATGGTAGCAGCTCGTTTGGTTTCGTCTGATCCTGATCTTTCTCAACGTATGTTTT<br>TCCATACTGTTGATATAATGGATGTAACCGAGAGCTTGAAGGGCTTCGACGTTGTGTTCTTGGCTGCTCTTGTAGGGATGGATAAAAAGG<br>AGAAGGTTAAGGTGGTCGAGCATCTTGAGAAACACATGTCTCCTGGTGCTTTGCTCATGCTGAGAAGCGCTCATGGACCTAGAGCTTTT<br>CTCTATCCAATCGTTGAGCCTTGTGATCTCGAAGGTTTCGAAGTTTTATCGGTTTATCACCCTACCGATGAAGTTATCAACTCCATTGTAAT<br>CTCAAGGAAGCTAGGTGAAGATGCTAATGGTGTTGTTCATGATCATATAGATCAAGCTTCGGATCTCGCGTGTAACTGTTCCAAGATCCAC<br>GTGATTATGAACAAGAAGAAGAGCATTATCGAGGAGTTCGCAGGTGCTAATGAAGAACAACTTACCTAG
HORVU.MOREX.r3.6HG0628880	ATGGACGCCCAGAGCAAGGAGGTCGACGCCCTTGTCCAGAAGATCACCGGCCTCCACGCCGCCATCGCCAAGCTGCCCTCGCTCAGC<br>CCGTCCCCGGACGTCGACGCGCTCTTCACCGACCTGGTCACCGCGTGCGTGCCCCCGAGCCCCGTGGACGTGACCAAGCTCGCCCC<br>GGAGGCGCAGGCGATGCGGGAGGGCCTCATCCGCCTCTGCTCCGAGGCCGAGGGCAAGCTGGAGGCGCACTACTCCGACATGCTCG<br>CCGCCTTCGACAACCCGCTCGACCACCTCGGCGTCTTCCCCTACTACAGCAACTACATCAACCTCAGCAAGCTCGAGTACGAGCTCCT<br>CGCGCGCTACGTGCCCGGCGGCATCGCCCCGGCCCGCGTCGCCTTCATCGGCTCCGGCCCGCTGCCGTTCAGCTCCTACGTCCTCG<br>CCGCGCGCCACCTGCCCGACACCGTGTTCGACAACTACGACCTGTGCGGCGCGGCCAACGACCGCGCGAGCAGGCTGTTCCGCGCG<br>GACAAGGACGTCGGCGCCCGCATGTCGTTCCACACCGCCGACGTCGCGGACCTCACCGACGAGCTCGCTACGTACGACGTCGTCTTC<br>CTGGCCGCGCTCGTTGGCATGGCCGCCGAGGACAAGGCCAAGGTGATCGCGCACCTTGGCGCGCACATGGCGGACGGGGCGGCCC<br>TCGTCGTGCGCAGCGCGCACGGGGCGCGTGGGTTCCTCTACCCGATCGTCGATCCCCAGGACATTGGTCGAGGCGGGTTCGAGGTG<br>CTCGCCGTGTGTCACCCCGACGACGACGTGGTGAACTCCGTCATCATCGCACAAAAGAGCAACGACGTGCACGAGTATGGACTTGGCA<br>GCGGGCGTGGTGGACGGTACGCGCGAGGCACGGTGGTGCCGGTGGTCAGCCCACCCTGCAGGTTCGGCGAGATGGTGGCAGACGT<br>GACCCAGAAGAGAGAGGAGTTTGCCAAGGCGGAAGTGGCCTTCTGA
HORVU.MOREX.r3.4HG0417690	ATGGCTGCCCAGAACAACAACAAGGATGTCGCTGCCCTGGTGGAGAAGATCACCGGGCTCCACGCCGCCATCGCCAAGCTGCCGTCG<br>CTCAGCCCATCCCCGGACGTCGACGCGCTCTTCACCGAGCTGGTCACGGCGTGCGTTCCCCCGAGCCCCGTGGACGTGACCAAGCT<br>CGGCCCCGAGGCGCAGGAGATGCGGGAGGGCCTCATCCGCCTCTGCTCCGAGGCCGAGGGGAAGCTGGAGGCGCACTACTCCGAC<br>ATGCTCGCCGCCTTCGACAACCCGCTGGATCACCTCGGCATCTTCCCCTACTACAGCAACTACATCAACCTCAGCAAGCTGGAGTACGA<br>GCTCCTGGCGCGCTACGTGCCCGGCGGCATCGCCCCGGTCCGCGTCGCGTTCATCGGCTCCGGCCCGCTGCCGTTCAGCTCCTTTG<br>TCCTGGCCGCGCGCCACCTGCCCGACACCATGTTTGACAACTACGACCTTTGCGGCGCGGCCAACGATCGCGCCAGCAAGCTCTTCC<br>GCGCGGACACGGACGTGGGTGCCCGCATGTCGTTCCACACGGCCGACGTCGCGGACCTCGCCAGCGAGCTCGCCAAGTACGACGTC

	GTCTTCCTGGCCGCGCTCGTCGGCATGGCTGCCGAGGACAAGGCCAAGGTGATCGTGCACCTCGGCGCACACATGGCAGACGGGGC<br>GGCCCTCGTCGTGCGCAGCGCACACGGAGCGCGCGGGTTCCTGTACCCGATTGTCGACCCCCAGGACATCGGCCGCGGCGGGTTCG<br>AGGTGCTGGCCGTGTGCCACCCCGACGACGACGTGGTGAACTCCGTCATCATCGCACAGAAGTCCAAGGAGGTGCATGCCGATGGAC<br>TTGGCAGCGCGCGTGGTGCCGGTGGACAGTACGCGCGCGGCACGGTGCCGGTTGTCAGCCCCCCGTGCAGGTTCGGTGAGATGGT<br>GGCGGACGTGACCCAGAACCACAAGAGAGACGAGTTTGCCAACGCCGAAGTGGCCTTTTGA
HORVU.MOREX.r3.2HG0099840	ATGGAGGCCGAAAACGGCGAGGTGGCTGCTCTGGTCGAGAAGATCACCGGTCTCCACGCCGCCATCTCCAAGCTCCCGTCACTAAGC<br>CCGTCTCCTCAAGTCGACGCGCTCTTCACCGAGCTGGTCGCGGCGTGCGTCCCATCAAGCCCGGTGGACGTGACCAAGCTCGGCCC<br>GGAGGCGCAGGAGATGCGGCAGGACCTCATCCGCCTCTGCTCGGCCGCCGAGGGGCTGCTCGAGGCGCACTACTCCGACATGCTCA<br>CCGCGTTGGACAGCCCGCTCGACCACCTCGGCCGCTTCCCTTACTTTGACAACTACGTCAACCTCAGCAAGCTCGAGCACGATCTTCT<br>GGCAGGTCACGTGGCGGCCCCGGCCCGCGTGGCGTTCATCGGGTCGGGGCCACTGCCGTTCAGCTCGCTCTTCCTCGCGACGTACC<br>ACCTGCGGGACACCCGGTTCGACAACTACGACCGGTGCAGCGTGGCGAACGGCCGGGCGATGAAGTTGGTCGGCGCGGCGGACGA<br>GGGCGTGCGATCACGCATGGCGTTCCACACGGCCGAAGTCACGAACCTCACGGCTGAGCTCGGCACTTACGACGTGGTTTTCCTGGC<br>CGCGCTCGTGGGAATGACGTCCGAGGAGAAGGCCGACGCCATAGCGCACTTGGGGAAGCACATGGCAGATGGGGCGGTGCTCGTGG<br>CGCGAAGCGCGCACGGGGCGCGAGCGTTCCTGTATCCTGTAGTGGAGCTGGACGATGTCGGGCGTGGCGGGTTCCAAGTGCTGGCG<br>GTGCACCACCCTGCAGGCGATGAGGTGTTCAACTCATTCATAGTTGCCCGGAAGGTGAAAATGAGTGCTTAA
LOC_Os03g19427	ATGGAGGCTCAGAACCAAGAGGTCGCTGCCCTGGTCGAGAAGATCGCCGGCCTCCACGCCGCCATCTCCAAGCTGCCGTCGCTGAGC<br>CCATCCGCCGAGGTGGACGCGCTCTTCACCGACCTCGTCACGGCGTGCGTCCCGGCGAGCCCCGTCGACGTGGCCAAGCTCGGCCC<br>GGAGGCGCAGGCGATGCGGGAGGAGCTCATCCGCCTCTGCTCCGCCGCCGAGGGCCACCTCGAGGCGCACTACGCCGACATGCTCG<br>CCGCCTTCGACAACCCGCTCGACCACCTCGCCCGCTTCCCGTACTACGGCAACTACGTCAACCTGAGCAAGCTGGAGTACGACCTCCT<br>CGTCCGCTACGTCCCCGGCATTGCCCCCACCCGCGTCGCCTTCGTCGGGTCGGGCCCGCTGCCGTTCAGCTCCCTCGTGCTCGCCG<br>CGCACCACCTGCCGGACGCGGTGTTCGACAACTACGACCGGTGCGGCGCGGCCAACGAGCGGGCGAGGAGGCTGTTCCGCGGCGC<br>CGACGAGGGCCTCGGCGCGCGCATGGCGTTCCACACCGCCGACGTGGCGACCCTGACGGGGGAGCTCGGCGCGTACGACGTCGTG<br>TTCCTGGCGGCGCTCGTGGGCATGGCGGCCGAGGAGAAGGCCGGGGTGATCGCGCACCTGGGCGCGCACATGGCGGACGGCGCG<br>GCGCTCGTCGTGCGGAGCGCGCACGGGGCGCGCGGGTTCCTGTACCCGATCGTCGATCCCGAGGACGTCAGGCGTGGCGGGTTCG<br>ACGTTCTGGCGGTGTGCCACCCGGAGGACGAGGTGATCAACTCCGTCATCGTCGCCCGCAAGGTCGGTGCCGCCGCCGCCGCCGCC<br>GCGGCGCGCAGAGACGAGCTCGCGGACTCGCGCGGCGTGGTTCTGCCGGTGGTCGGGCCGCCGTCCACGTGCTGCAAGGTGGAGG<br>CGAGCGCGGTTGAGAAGGCAGAAGAGTTTGCCGCCAACAAGGAGCTGTCCGTCTAA
LOC_Os03g19420	ATGGAGGCTCAGAACCAAGAGGTCGCTGCCCTGGTCGAGAAGATCGCCGGCCTCCACGCCGCCATCTCCAAGCTGCCGTCGCTGAGC<br>CCATCCGCCGAGGTGGACGCGCTCTTCACCGACCTCGTCACGGCGTGCGTCCCGGCGAGCCCCGTCGACGTGGCCAAGCTCGGCCC<br>GGAGGCGCAGGCGATGCGGGAGGAGCTCATCCGCCTCTGCTCCGCCGCCGAGGGCCACCTCGAGGCGCACTACGCCGACATGCTCG<br>CCGCCTTCGACAACCCGCTCGACCACCTCGCCCGCTTCCCGTACTACGGCAACTACGTCAACCTGAGCAAGCTGGAGTACGACCTCCT<br>CGTCCGCTACGTCCCCGGCATTGCCCCCACCCGCGTCGCCTTCGTCGGGTCGGGCCCGCTGCCGTTCAGCTCCCTCGTGCTCGCCG<br>CGCACCACCTGCCGGACGCGGTGTTCGACAACTACGACCGGTGCGGCGCGGCCAACGAGCGGGCGAGGAGGCTGTTCCGCGGCGC

	CGACGAGGGCCTCGGCGCGCATGGCGTTCCACACCGCCGACGTGGCGACCCTGACGGGGAGCTCGGCGCGTACGACGTCGTG<br>TTCCTGGCGGCGCTCGTGGGCATGGCGGCCGAGGAGAAGGCCGGGGTGATCGCGCACCTGGGCGCGCACATGGCGGACGGCGCG<br>GCGCTCGTCGTGCGGAGCGCGCACGGGGCGCGCGGGTTCCTGTACCCGATCGTCGATCTCGAGGACATCCGGCGGGGCGGGTTCG<br>ACGTGCTGGCCGTGTACCACCCCGACGACGAGGTGATCAACTCCGTCATCGTCGCTCGCAAGGCCGACCCGCGTCGCGGCGGCGGG<br>CTCGCCGGCGCACGCGGCGCGGTTCCAGTGGTGAGCCCGCCGTGCAAGTGCTGCAAGATGGAGGCGGCCGCCGGCGCGTTCCAGA<br>AGGCGGAAGAGTTCGCCGCCAAGAGGCTATCCGTCTGA
LOC_Os07g<br>48980	ATGACGGTGGAAGTGGAGGCGGTGACCATGGCGAAGGAGGAGCAGCCGGAGGAGGAGGAGGTGATCGAGAAGTTGGTTGAGAAGAT<br>CACCGGGCTGGCGGCGGCCATCGGCAAGCTGCCGTCGCTGAGCCCGTCGCCGGAGGTGAACGCGCTGTTCACGGAGCTGGTGATG<br>ACCTGCATCCCGCCCAGCAGCGTGGACGTGGAGCAGCTGGGGGCGGAGGCGCAGGACATGCGCGGCCGCCTCATCCGCCTCTGCG<br>CCGACGCCGAGGGCCACCTCGAGGCGCACTACTCCGACGTCCTCGCCGCCCACGACAACCCGCTCGACCACCTCGCCCTCTTCCCC<br>TACTTCAACAACTACATCCAGCTCGCCCAGCTCGAGTACGCCCTCCTCGCCCGCCACCTCCCCGCCGCCCCGCCGCCCTCCCGCCTC<br>GCCTTCCTCGGCTCCGGCCCGCTCCCGCTCAGCTCCCTCGTCCTCGCCGCCCGCCACCTCCCCGCCGCCTCCTTCCACAACTACGAC<br>ATCTGCGCCGACGCCAACCGCCGCGCCAGCCGCCTCGTCCGCGCCGACCGCGACCTGTCCGCGCGCATGGCCTTCCACACCTCCGA<br>CGTCGCCCACGTCACCACCGACCTCGCCGCCTACGACGTCGTGTTCCTGGCGGCGCTGGTGGGTATGGCCGCCGAGGAGAAGGCGC<br>GCATGGTGGAGCACCTCGGGAAGCACATGGCGCCCGGCGCCGCCCTGGTGGTGCGGAGCGCGCACGGCGCCCGGGGATTCCTGTA<br>CCCCGTGGTGGACCCGGAGGAGATCCGCCGCGGCGGCTTCGACGTGCTCGCCGTGCACCACCCGGAGGGCGAGGTGATCAACTCC<br>GTCATCATCGCGCGCAAGCCGCCCGTGGCGGCGCCGGCGTTGGAGGGAGGAGACGCGCACGCGCACGGCCATGGCGCCGTGGTGA<br>GCCGTCCATGCCAGCGCTGCGAGATGGAGGCGAGGGCGCACCAGAAGATGGAGGACATGTCCGCCATGGAGAAGCTGCCCTCCTCG<br>TAG
TraesCS2A0<br>2G033500	ATGGAGGCCGAAAACAGCGAGGTGGCTGCTCTGGTCGAGAAGATCACCGGCTTCCACGCCGCCATCTCCAAGCTCCCGTCGCTAAGC<br>CCGTCCCCTCAAGTCGACGCGCTCTTCACCGAGCTGGTCGCGGCGTGCGTCCCGTCGAGCCCGGTGGACGTGACCAAGCTCGGCCC<br>GGAGGCGCAGGAGATGCGGCAGGACCTCATCCGCCTGTGCTCAACCGCCGAGGGGCTGCTCGAGGCGCACTACTCCGACATGCTCA<br>CCGCCTTGGACAGCCCGCTCGACCACCTCGGCCGCTTCCCTTACTTTGACAACTACATCAACCTGAGCAAGCTCGAGAACGACCTTCT<br>GGCCGGTCACATGGCGGCTCCGGCCCGCGTGGCGTTCATCGGGTCAGGGCCGCTGCCGTTCAGCTCGCTCTTCCTCGCGACATACCA<br>CCTGCCGGACACCCGCTTCGACAACTACGACCGGTGCAGCGTGGCCAATGGCCGGGCGATGAAGCTGGTCGGCGCGGCGGATGTGG<br>ACGTGCGCTCGCGCATGCCGTTCCACACGGCCGAAGTCGCGGACCTCACGTCTGAGCTCGGCGCGTACGACGTGGTTTTCCTGGCG<br>GCGCTCGTGGGGATGACGTCCGAGGAGAAGGCCAACACCATCGCGCACTTGGGGAAGCACATGGCAGATGGGGCGGTGCTCGTCGC<br>GCGAAGCGCGCACGGGGCGCGAGCGTTCCTCTATCCTGTAGTGGAGCTGGACGATATCGGGCGTGGCGGGTTCCAAGTGCTGGCCG<br>TGCACCACCCTGCAGGTGATGAGGTGTTCAACTCATTCATTGTTGCACAGAAGGTGAAGATATGA
TraesCS2B0<br>2G047100	ATGGAGGCCGAAAACAGCGAGGTGGCTGCTCTGGTCGAGAAGATCACCGGCCTCCACGCCGCCATCTCCAAGCTCGCGTCTCTAAGC<br>CCGTCCCCCGAAGTCGACGCGCTCTTCACGGAGCTGGTCGCGGCGTGCGTCCCGTCGAGCCCGGTGGACGTGACCAAGCTCGGCC<br>CGGAGGCGCAGGAGATGCGGCAGGACCTCATCCGCCTCTGCTCGGCCGCCGAGGGGCTGCTCGAGGCGCACTACTCCAACATGCTC<br>ACCGCCTTGGACAACCCGCTCGACCACCTCGGCCGCTTCCCTTACTTCGACAACTACATCAACCTGAGCAAGCTCGAGCACGATCTTCT

	TGCCGGTCACGTGGCGGCCCCGGCCCGCGTGGCGTTCATCGGGTCGGGGCCGCTGCCGTTCAGCTCCCTCTTCCTCGCGATGTACC<br>ACCTGCCGGACACCCGGTTCGACAACTACGACCGGTGCAGCGTGGCCAATGGCCGTGCGATGAAGCTGGTCGGCGCGGCGGACGAG<br>GGCGTGCGTGCGCGCATGGCGTTCCACACGGCCGAAGTCGCGGACCTCACGGCTGAGCTCGGCGCGTATGATGTGGTCTTCCTGGC<br>GGCGCTCGTGGGAATGACGTCCGAGGAGAAGGCCAACACCATCGCGCACTTGGGGAAGCACATGGCAGATGGCGCGGTGCTCGTCG<br>CGCGAAGCGCCCACGGTGCGCGAGCGTTCCTGTATCCTGTAGTGGAGCTGGACGATATCGGGCGTGGCGGGTTCCAAGTGCTGGCTG<br>TGCACCATCCTGCGGGCGATGAGGTGTTCAACTCATTTATTGTTGCGCGGAAGGAGCATATTTCTACCGTGGGCACTATAGGGTGCTCAT<br>GGCAGCACGAATGTATATGGACTACAATTGAGGGTGTGGCAGACGGAGAGATGTCATTCAAGTAA
TraesCS6A02G163100	ATGGATGCCCAGAAGATGGAGGTCGCTGCTCTGATCGAGAAGATCGCCGGTCTCCAGGCCGCCATCGCCGGGCTGCCGTCGCTGAGC<br>CCGTCCCCTGAGGTCGACAGGCTCTTCACCGACCTCGTCACCGCGTGCGTCCCGCCGAGCCCCGTCGACGTGACGAAGCTCAGCCC<br>GGAGCACCAGAGGATGCGGGAGGCGCTCATCCGCCTCTGCTCCGCCGCCGAGGGGAAGCTCGAGGCGCACTACGCCGACCTGCTC<br>GCCACCTTCGACAACCCGCTCGACCACCTCGGCCGCTTCCCCTACTACAGCAACTACGTCAACCTCAGCAGGCTGGAGTACGAGCTGC<br>TGGCGCGCCACGTGCCGGGCATCGCGCCGGCGCGCGTCGCCTTCGTCGGCTCCGGCCCGCTGCCGTTCAGCTCGTTCGTCCTCGCC<br>GCGCACCACCTGCCCGACGCGCAGTTCGACAACTACGACCTGTGCGGCGCGGCCAACGAGCGCGCCAGGAAGCTGTTCGGCGCGA<br>GGGAGGACGGCGTGGGCGCGCGCATGAAGTTCCACACGGCGGACGTCGCCGACCTCACGCAGGAGCTCGGTGCGTACGACGTGGT<br>CTTCCTCGCCGCGCTCGTCGGCATGGCGGCCGAGGAGAAGGCCAAGGTGATAGCCCACCTGGGCGCGCACATGGTGGAGGGGGCGT<br>CCCTGGTCGTGCGGAGCGCGCACGGCGCTCGCGGCTTCCTGTACCCCATCGTCGACCCGGAGGACATCAGGCGGGGCGGGTTCGA<br>GGTGCTGGCCGTGCACCACCCCGAAGGTGAGGTGATCAACTCTGTCATCGTCGCCCGTAAGGCCGTCGACGCGCAGCTCAGTGGGC<br>CGCAGAACGGAGCACGGGGCGCGGTGCCGCTGGTCAGCCCGCCATGCAGCTTCTCCACCAAGATGGAGGCGAGCGCGCTTGAGAAG<br>AGCGAAGAGTTGGCCACCAAAGAGCTGGCCTTTTGA
TraesCS6D02G148200	ATGGATGCCCAGAACAACGAGGTCGCTGCTCTGATCGAGAAGATCGCCGGTCTCCAGGCCGCCATCGCTGAGCTGCCGTCGCTGAGC<br>CCGTCCCCCGAGGTCGACAGGCTCTTCACCGCCCTCGTCACGGCCTGCGTCCCGCCGAGCCCCGTCGACGTGACGAAGCTCAGCCC<br>GGAGCACCAGAGGATGCGGGAGGCGCTCATCCGCCTCTGCTCCGCCGCCGAGGGGAAGCTCGAGGCGCACTACGCCGACCTGCTC<br>GCCACCTTCGACAACCCGCTCGACCACCTCGGCCGCTTCCCCTACTACAGCAACTATGTCAACCTCAGCAGGCTGGAGTACGAGCTCC<br>TGGCGCGCCACGTGCCGGGCATCGCGCCGGCGCGCGTCGCCTTCGTCGGCTCCGGCCCGCTGCCGTTCAGCTCGTTCGTCCTCGCC<br>GCGCACCACCTGCCCGACGCCCAGTTCGACAACTACGACCTGTGCGGCGCGGCCAACGAGCGCGCCAGGAAGCTGTTCGGCGCGAG<br>CGAGGACGGCGTGGGCGCGCGCATGAAGTTCCACACGGCGGACGTCGCCGACCTCACGCAGGAGCTCGGCGCGTACGACGTGGTC<br>TTCCTCGCCGCGCTCGTCGGCATGGCGGCAGAGGAGAAGGCCAAGGTGATAGCCCACCTGGGCGCGCACATGGTGGAGGGGGCGTC<br>CCTGGTCGTGCGGAGCGCGCACGGCGCCCGCGGCTTCCTGTACCCCATCGTCGACCCGGAGGACATCAGGCGGGGCAGGTTCGAG<br>GTGCTGGCCGTGCACCACCCCGAAGGTGAGGTGATCAACTCTGTCATCGTCGCCCGTAAGGTCGTCGACGCGAAGCTCAGTGGGCCG<br>CAGAACGGAGACGCGCACGCACGGGGCGCGGTGCCGCTGGTCAGCCCGCCATGCAGCTTCTCCACCAAGATGGAGGCGGGCGCGC<br>TTGAGAAGAGCGAAGAGCTGGCCGCCAAAGAGCTGGCCTTTTGA
TraesCS6D02G148600	ATGGATGCCCAGAACAAGGAGGTCGCTGCTCTGATCGAGAAGATCGCCGGTCTCCAGTCCGCCATCGCCGAGCTGCCGTCGCTGAGC<br>CCGTCCCCCGAGGTCGACAGGCTCTTCACCGACCTCGTCACGGCCTGCGTCCCGCCGAGCCCCGTCGACGTGACGAAGCTCAGCCC

	GGAGCACCAGAGGATGCGGGAGGCGCTCATCCGCCTCTGCTCCGCCGCCGAGGGGAAGCTCGAGGCGCACTACGCCGACCTGCTC<br>GCCACCTTCGACAACCCGCTCGACCACCTCGCCCGCTTCCCCTACTACAGCAACTACGTCAACCTCAGCAGGCTGGAGTACGAGCTCC<br>TGGCGCGCCACGTGCCGGGCATCGCGCCGGCGCGCGTCGCCTTCATCGGCTCCGGCCCGCTGCCGTTCAGCTCGTTCGTCCTCGCC<br>GCGCACCACCTGCCCGACGCGCACTTCGACAACTACGACCTGTGCGGCGCGGCCAACGAGCGCGCCAGGAAGCTGTTCGGCGCGAG<br>CGAGGACGGCGTGGGCGCGCGCATGAAGTTCCACACGGCGGACGTCGCCGACCTCTCGCAGGAGCTCGGCGCGTACGACGTGGTC<br>TTCCTCGCCGCGCTCGTCGGCATGGTGGCCGAGGAGAAGGCCAAGGTGATAGCCCACCTGGGCGCGCACATGGTGGAGGGGGCGTC<br>CCTGGTCGTGCGGAGCGCGCACGGCGCCCGCGGCTTCCTGTACCCCATCGTCGACCCGGAGGACATCAGGCGAGGCGGGTTCGAG<br>GTGCTGGCCGTGCACCACCCCGAAGGTGAGGTCATCAACTCTGTCATTGTCGCCCGTAAGGTCGTCGACGCGCAGCTCAGTGGGCCG<br>CTGAACGGAGACGCGCACGCGCGGGGCGCGGTGCCGCTGGTCAGCCCGCCGTGCAGCTTCTCCACCAAGATGGAGGCGGGCGCGC<br>TTGAGAAGAGCGACGAGTTGGCCACCAAAGAGCTGGCCTTTTGA
TraesCS2A02G049900	ATGGCTCTCCAGAACAAGGAGGTGGATGCCCTGGTCCAGAAGATCACCGGACTCCACGCCGCCATCGCCAAGCTGCCGTCGCTCAGC<br>CCGTCCCCGGACGTAGACGCGCTCTTCACCGAGCTGGTCACCGCGTGCGTTCCCCCGAGCCCCGTGGACGTGACCAAGCTCGGCCC<br>GGAGGCGCAGGAGATGCGGGAGGGCCTCATCCGCCTCTGCTCTGAGGCCGAGGGGAAGCTGGAGGCGCACTACTCCGACATGCTCG<br>CGGCCTTCGACAACCCGCTCGACCACCTCGGTATGTTCCCCTACTACAACAACTACATCAACCTCAGCAAGCTTGAATACGAGCTCCTG<br>GCGCGCTACGTGCCCGGCGGCATCGCCCCGGCCCGCGTCGCCTTCATCGGCTCCGGCCCACTGCCGTTCAGCTCTTTCGTCCTCGC<br>CGCACGCCACTTGCCCGACACCATGTTCGACAACTACGACCTATGTGGCGCGGCCAATGACCGCGCCAGCAAGCTGTTCCGCGCGGA<br>CAAGGACGTGGGCGCCCGCATGTCGTTCCACACGGCCGACGTCGCGGACCTTGCCGGCGAGCTCGCTAAGTACGACGTCGTCTTCCT<br>GGCCGCGCTCGTGGGCATGGCCGCAGAGGACAAGGCCAAGGTGATCGCGCACCTCGGCGCACACATGGCAGACGGGGCGGCCCTC<br>GTTGTACGCAGTGCGCACGGGGCACGCGGGTTCCTGTACCCGATCGTAGACCCCCAGGACATCGCCGGAGGCGGGTTCAAGGTGCT<br>CGCCGTGTGCCACCCCGACGACGACGTGGTGAACTCCGTTATCATCGCACAGAAGTCCAAGGACTTGCATGCCAATGGACATCACCGT<br>GGGCATGGTGGACAGTGCGCGCATGGCACGGTGCCGGTGGTCAACCCACCGTGCAGGTTTGGTGAGATGGTGACAGACATGGCCCA<br>GAAGAGAGAGGAGTTTGCCAACGCCGAAGTGGTCTTTTGA
TraesCS2B02G060800	ATGGCTGCCCAGAACAAGGAGGTGGATGCCCTGGTCCAGAAGATCACCGGACTCCACGCCGCCATCGCCAAGCTGCCGTCGCTCAGC<br>CCGTCCCCGGACGTCGACGCGCTCTTCACCGAGCTGGTCACCGCGTGCGTTCCTCCGAGCCCTGTGGACGTGACCAAGCTCGGCCC<br>GGAGGCGCAGGAGATGCGGGAGGGCCTCATCCGCCTCTGCTCCGAGGCCGAGGGAAAGCTGGAGGCGCACTACTCCGACATGCTCG<br>CTGCCTTTGACAACCCACTCGACCACCTAGGTATGTTCCCCTACTACAACAACTACATCAACCTCAGCAAGCTTGAGTACGAGCTCCTGG<br>CGCGCTATGTGCCCGGTGGCATCGCCCCGGCCCGGGTCGCCTTCATCGGCTCCGGCCCACTGCCGTTCAGCTCTTTCGTCCTCGCCG<br>CGCGCCACTTGCCCGACACCATGTTCGACAACTATGACCTATGTGGGGCGGCCAACGACCGCGCCAGCAAGCTGTTCCGCGCGGACA<br>AGGACGTGGGCGCCCGCATGTCGTTCCACACGGCCGATGTAGCGGACCTCGCTGCTGAGCTCGCTACATACGACGTCGTCTTCCTGG<br>CTGCGCTCGTGGGCATGGCCGCGGAGGACAAGGCCAAGGTGATCGCGCACCTCGGTGCACACATGGCAGACGGTGCGGCCCTTGTT<br>GTACGCAGTGCACACGGGGCACGCGGGTTCCTGTACCCGATCGTCGACCCCCAGGACATCGCAGGAGGCGGGTTCGAGGTGCTGGC<br>CGTGTGCCACCCCGATGACGACGTGGTGAACTCCGTCATCATCGCACAGAAGTCCAAGGACGTGCTTGCCAATGGACTTCGCCGCGG

	GCATGGTGGACAGTACGCGTGCGGCACGGTGCCGGTGGTCAGCCCACCGTGCAGGTTCGGTGAGATGGTGACGGACGTGACCCACAAGAGAGAGGAGTTTGCCAACGCCGAAGTGGTCTTTTGA
TraesCS5A02G552400	ATGGCTGCCCAGAACAACAAGGAGGTGGATGCCCTGGTGGAGAAGATCACCGGGCTCCACGCCGCCATCGCCAAGCTGCCGTCGCTCAGCCCTTCCCCGGCCGTCGACCTGCTCTTCACCGAGCTGGTCACGGCGTGCGTTCCCCCGAGCCCCGTGGACGTGACCAAGCTCGGCCCGGAGGCGCAGGAGATGCGGGAGGGCCTCATCCGCCTCTGCTCCGAGGCCGAGGGGAAGCTGGAGGCGCACTACTCCGACATGCTCGCCGCCTTCGACAACCCTCTGGACCACCTCGGCATGTTCCCCTACTACAGCAACTACATCAACCTCAGCAAGCTGGAGTACGAGCTCCTGGCCCGCTACGTGCCTGGTGGCATCGCCCCTGCCCGCGTCGCCTTCATCGGCTCCGGCCCGCTGCCGTTCAGCTCCTTCGTCCTCGCCGCGCGCCACCTGCCCGACACCATGTTCGACAACTACGACCTGTGCGGCGCGGCCAATGACCGCGCCAGCAAGCTGTTCCGCGCCGACAAGGACATGGGCGCCCGCATGTCGTTCCACACGGCCGACGTAGCGGACCTCGCTGGCGAGCTCGCCAAGTACGACGTGGTCTTCCTGGCCGCACTTGTTGGCATGGCGGCTGAGGACAAGGCCAAGGTGATAGCACACCTCGGCACACACATGGCAGACGGGGCGGCCCTCGTCGTGCGCAGCGCACACAGGGCACGCGGGTTCCTGTACCCGATCGTCGACCCCCAGGACATCACCCTAGGTGGGTTCAAGGTGTTGGCCGTGTGCCATCCAGACGACGACGTGGTGAACTCCGTCATCATCGCACAGAAGTCCAAGGACGTGCATGTTAGTGGACTTCGCAGCGGGCCTGCTGTGGGTGGACAGTCTGCTCGCTCCACGGTGCCGGTGGTCAGCCCGCCGTGCAGGTTCGGTGAGATGGTGGCGGATATGACACAGAAGAGAGAGGAGTTTGCCAACGCCGAAGTGGCCTTTTGA
TraesCSU02G125200	ATGGCTGCCCAGAACAACAAGGAGGTGGATGCCCTGGTGGAGAAGATCACCGGCCTCCACGCCGCCATCGCCAAGCTGCCGTCGCTCAACCCATCCCCGGACGTCGACGCGCTCTTCACTGAGCTGGTCACGGCGTGCGTTCCCCCGAGCCCGGTGGACGTGACCAAGCTCGGCCCGGGGGCGCAGGAGATGCGGGAGGGCCTCATCCGCCTTTGCTCCGAGGCCGAGGGGAAGCTGGAGGCGCACTACTCCGACATGCTTGCCGCCTTCGACAACCCTCTGGATCACCTCGGCATGTTCCCCTACTACAGCAACTACATCAACCTCAGCAAGCTTGAGTACGAGCTCCTGGCGCGCTACGTGCCCGGTGGCATCGCCCCTGCCCGCGTCGCCTTCATCGGCTCCGGCCCACTCCCGTTCAGCTCCTTTGTCCTGGCCGCGCGCCATCTGCCCGACACCATGTTCGACAACTACGACCTGTGCGGTGCGGCCAACGACCGTGCCAGCAAGCTGTTCCGTGCGGACACGGACGTGGGCGCCCGCATGTCGTTCCACACGGCCGACGTAGCGGACCTCGCCGGCGAGCTCGCCAAGTACGACGTGGTCTTCCTGGCCGCACTTGTTGGCATGGCCGCCGAGGACAAGGCGAAGGTGATCGCACACCTCGGCGCACATATGGCGGACGGTGCGGCTCTCGTCGTGCGCAGCGCTCACGGGGCACGCGGGTTCCTGTACCCGATCGTCGACCCCCAGGACATCACCCTAGGCGGGTTCGAGGTGCTGGCCGTGTGCCACCCAGACGACGACGTGGTGAACTCCGTCATCATCGCACAGAAGTCCAAGGACGTGCATGTGAGTGGACTTCACAGTGGGCGTGCTGTGGGTGGACAGTCTGCTCGCGGCACGGTGCCGGTGGTCAGCCCGCCGTGCAGGTTCGGTGAGATGGTGGCGGAGGTGACGCAGAAGAGAGAGGAGTTCGCCAACGCCGAAGTGGCCTTCTGA
TraesCS3B02G068500	ATGTTTGACAACTACGACCTGTGCGGCGCGGCCAACGAGCGCGCCAGCAAGCTGTTCCGCGCGGACACGGACGTGGGCGCCCGCATGTCGTTCCACACGGCCGACGTCGCGGACCTCGCCGGCGAGCTTGCCAAGTACGACGTCGTCTTCCTGGCGGCGCTCGTGGGCATGGCCGCCGAGGAAAAGGCCAGGGTGATCGCGCACCTTGGCACGCACATGGCAGACGGGGCGGCCCTCGTCGTGCGCAGCGCGCACGGCGCACGCGGGTTCCTATACCCGATCGTGGACCCCCAGGACATCGGCCGAGGCGGGTTTGAGGTGCTGGCCGTGTGCCACCCCGACGACGACGTGGTGAACTCCGTCATTATCGCACACAAGTCGAAGGACATGCATGCCAGTGGACTTCGCAGCGAGCGTGCCGGTGGTGGGCAGTATGCGCGTGGCACGGTGCCGGTGGTCAGCCCGCCGTGCAGGTTCGGCAAGATGGTGGCCGACGTGAACCAGAAGAGAGAGGAGTTTGCCAAGGCCGAAGTGGCTTTTTGA

TraesCS4B02G183900	ATGGATGCCCAGAACAAGGAGGTCGACGCCCTGGTCCACAAGATCACCGGCCTCCACGCCGCCATCGCCAAGCTGCCGTCCCTCAGC<br>CCATCCCCCGACGTCGACGCGCTCTTCACCGACCTGGTCACCGCGTGCGTCCCCCCGAGCCCCGTGGACGTGACCAAGCTCGGCCC<br>GGAGGCGCAGGAGATGCGGGAGGGCCTCATCCGCCTCTGCTCCGAGGCCGAGGGGAAGCTGGAGGCGCACTACTCCGACATGCTCG<br>CCGCCTTCGACAACCCGCTCCACCACCTCGCCATCTTCCCCTACTACAGCAACTACATCAACCTCAGCAAGCTGGAGTACGAGCTCCTG<br>GCGCGCTACGTGCCCGGCGGCATCGCCCCGGCCCGCGTCGCGTTCATCGGCTCCGGCCCGCTGCCGTTCAGCTCCTACGTCCTCGC<br>CGCCCGCCACCTGCCCGACACCATGTTTGACAACTACGACCTGTGTGGCGCGGCCAACGACCGTGCGAGCAAGCTGTTCCGCGCGG<br>ACAAGGAAGTGGGCGCCCGCATGTCGTTCCACACCGCTGACGTCGCGGACCTTGCCGGCGAGCTCGCCGCGTACGACGTCGTCTTC<br>CTGGCCGCGCTCGTGGGCATGGCCGCCGAGGACAAGGCCAAGGTGATCGCACACCTCGGCGCGCACATGGCGGACGGGTCGGCCC<br>TCGTCGTGCGCAGCGCGCACGGGGCGCGTGGGTTCCTGTACCCGATCGTCGATCCCCAGGACATCGGCCGAGGCGGGTTTGAGGTG<br>CTGGCCGTGTGTCACCCCGACGACGACGTGGTGAACTCCGTCATCATCGCACAGAAGTCCAAGGACATGCATGCCAATGAACACCGCA<br>ACGGGCGTGGTGGACAGTACGCGCGGGGCACGGTGCCGGTGGTGAGCCCGCCGTGCAGGTTCGGCGAGATGGTGGCGGACGTGAC<br>CCAAAAGAGAGAGGAGTTCGCCAATGCCGAAGTGGCCTTCTGA
TraesCS4D02G184900	ATGCACAGCTTTGCCAGCTCCATCGATGAGTGCTCTAGACGGCAGGCAGCTACATATACCCCGTGCTCCTCGTTCCATAGCTCACCAAG<br>CAGCCCGATCCACCAACTCCACTCCACTCCTGTGCCTCAGAGTTCATCAAGTACTCGTCAGGTACCAGGTAAAATGGATGCCCAGAACA<br>AGGAGGTCGCCGCCCTGGTCCACAAGATCACCGGCCTCCACGCCGCCATCGCCAAGCTGCCGTCCCTCAGCCCATCCCCCGACGTC<br>GACGCGCTCTTCACCGACCTGGTCACCGCGTGCGTCCCCCCGAGCCCCGTGGACGTGACCAAGCTCGGCCCGGAGGCGCAGGAGAT<br>GCGGGAGGGCCTCATCCGCCTCTGCTCCGAGGCCGAGGGGAAGCTGGAGGCGCACTACTCCGACATGCTCGCCGCCTTCGACAACC<br>CGCTCGACCACCTCGGCATCTTCCCCTACTACAGCAACTACATCAACCTCAGCAAGCTGGAGTACGAGCTCCTGGCGCGCTACGTGCC<br>CGGCGGCATCGCCCCGGCCCGCGTCGCCTTCATCGGCTCCGGCCCGCTGCCGTTCAGCTCCTACGTCCTCGCCGCCCGCCACCTGC<br>CCGACACCATGTTCGACAACTACGACCTGTGCGGCGCGGCCAACGACCGCGCGAGCAAGCTGTTCCGCGCCGACAAGGACGTGGGC<br>GCCCGCATGTCGTTCCACACCGCCGACGTCGCGGACCTCGCCGGCGAGCTCGCCGCGTACGACGTCGTCTTCCTGGCCGCGCTCGT<br>GGGCATGGCTGCCGAGGACAAGGCCAAGGTGATCGCGCACCTCGGCGCGCACATGGCGGACGGGGCGGCCCTCGTCGTGCGCAGC<br>GCGCACGGCGCGCGTGGGTTCCTGTACCCGATCGTCGATCCCCAGGACATCGGCCGAGGCGGGTTCGAGGTGCTGGCCGTGTGTCA<br>CCCCGACGACGACGTGGTGAACTCCGTCATCATCGCACAGAAGTCCAAGGACATGCATGCCAATGAACATCGCAACGGGCGTGGTGG<br>ACAGTACGCGCGGGGCACGGTGCCGGTGGTGAGCCCACCGTGCAGGTTCGGCGAGATGGTGGCGGACGCGGCCCAGAAGAGAGAG<br>GAGTTCGCCAACGCTGAAGTGGCCTTCTGA
TraesCS6A02G093000	ATGGATGCCCAGAACAAGGAGGTCGATGCCCTGGTCCAGAAGATCACCGGCCTCCACGCCGCCATCGCCAAGCTGCCCTCGCTCAGC<br>CCGTCCCCCGACGTCGACGCGCTCTTCACCGACCTGGTCACCGCGTGCGTCCCCCCGAGCCCCGTGGACGTGACCAAGCTCGGCCC<br>GGAGGCACAGGCGATGCGGGAGGGCCTCATCCGCCTCTGCTCCGAGGCCGAGGGGAAGCTGGAGGCGCACTACTCCGACATGCTCG<br>CCGCCTTCGACAACCCGCTCGACCACCTCGGCGTCTTCCCCTACTACAGCAACTACATCAACCTCAGCAAGCTGGAGTACGAGCTCCT<br>GGCGCGCTACGTCCCCGGCGGCATCGCCCCGGCCCGTGTCGCCTTCATCGGCTCCGGCCCGCTGCCGTTCAGCTCGTACGTCCTCG<br>CCGCACGCCACCTGCCCGACGCGGTGTTCGACAACTACGACCTGTGTGGCGCGGCCAACGACCGTGCGAGCAAGCTGTTCCGCGCC<br>GACAAGGACGTGGGCGCACGCATGTCATTCCACACCGCCGACGTAGCGGACCTCACCGACGAGCTCGCCACGTACGACGTCGTCTTC

	CTGGCCGCGCTCGTGGGCATGGCTGCCGAGGACAAGGCCAAGGTGATCGCACACCTTGGCGCGCACATGGCGGACGGGGCGGCCC<br>TCGTCGTGCGGAGCGCGCACGGGGCGCGTGGGTTCCTGTACCCGATCGTCGATCCCCAGGACATCGGCCGAGGCGGGTTCGAGGTG<br>CTCGCCGTGTGTCACCCCGACGACGACGTGGTGAACTCTGTCATCATCGCGCAGAAGTCCAGCGACATGCATGCGAATGGATTTCGCA<br>ACGGGCGTGGTGGACAGTGCGCGCGGGGCACGGTGCCGGTGGTCAGCCCACCCTGCAGGTTCGGCGAGATGGTGGCGGACGTGAG<br>CCAGAAGAGAGAGGAGTTTGCCAACGCGGAAGTGGCCTTCTAA
TraesCS6A02G386200	ATGGACGCCCAGAACAAGGAGGTGGACGCCCTGGTCCAGAAGATCACCGGCCTCCACGCCGCCATCGCCAAGCTGCCCTCGCTCAG<br>CCCGTGCCCCGCCGTCGACGCGCTCTTCACCGACCTCGTCACCGCGTGCGTCCCCCCGAGCCCCGTGGACGTGACCGAGCTCGGCC<br>CGGAGGCGCAGGCGATGCGCGAGGGCCTCATCCGCCTCTGCTCCGAGGCCGAGGGGAAGCTGGAGGCGCACTACTCCGACATGCTC<br>GCCGCCTTCGACAACCCGCTCGACCACCTCGGCGTCTTCCCTTACTACAGCAACTACATCAACCTCAGCAAGCTGGAGTACGAGCTCC<br>TGTCGCGCTACGTGCCCGGCGGCATCGCCCCGGCCCGTGTCGCCTTCATCGGCTCCGGCCCGCTGCCGTTCAGCTCCTACGTCCTCG<br>CCGCGCGCCACCTGCCCGACACGGTGTTCGACAACTACGACCTGTGCGGCGCGGCCAACGCCCGCGCGAGCAGGCTGTTCCGCGC<br>GGACAGGGACGTGGGCGCCCGCATGTCGTTCCACACCGCCGACGTCGCGGACCTCACCGACGTGCTCGCTACGTATGATGTCGTCTT<br>CCTGGCCGCGCTCGTGGGCATGGCGGCCGAGGACAAGGCCAAGGTGATCGCGCACCTTGGCGCGCACATGGCGGACGGGGCGGCC<br>CTCGTCGTGCGCAGCGCGCACGGGGCGCGTGGGTTCCTGTACCCGATCGTCGATCCCCAGGACATCGGCCGAGGCGGGTTCGAGGT<br>GCTCGCCGTGTGTCACCCAGACGACGACGTGGTGAACTCCGTCATCATCGCGCAAAAGACCAACGACGTGCATGAGTATGGACTTGGC<br>AACGGGCGTGGTGGACGGTACGCGCGGGGCACGGTGCCGGTGGTCAGCCCGCCCTGCAGGTTCGGCGAGATGGTGGCGGACGTGA<br>CCCAGAAGAGAGAGGAGTTTGCCAACGCGGAACTGGCCTTCTGA
TraesCS6D02G370800	ATGGACGCCCAGAACAAGGAGGTCGACGCCCTGGTCCAGAAGATCACCAGGCTCCACGCCGCCATCTCCAAGCTGCCCTCGCTCAGC<br>CCGTCCCCCGACGTCGACGCGCTCTTCACCGACCTCGTCACCGCGTGCGTCCCCGCGAGCGCCGTGGACGTGACCAAGCTCGGCCC<br>GGAGGCGCAGGCGATGCGGGAGGGCCTCATCCGCCTCTGCTCCGAGGCCGAGGGAAAGCTGGAGGCGCACTACTCCGACATGCTCG<br>CCGCCTTCGACAACCCGCTCGACCACCTCGGCGTCTTCCCCTACTACAGCAACTACATCAACCTCAGCAAGCTGGAGTACGAGCTCCT<br>CGCGCGCTACGTGCCCGGCGGCATCGCCCCGGCACGTGTCGCGTTCATCGGCTCCGGCCCGCTGCCGTTCAGCTCCTACGTCCTCG<br>CCGCGCGCCACCTGCCCGACACGGTGTTCGACAACTACGACCTGTGCGGCGCGGCCAACGCCCGTGCGAGCAAGCTGTTCCGTGCG<br>GACAGGGACGTGGGCGCCCGCATGTCGTTCCACACCGCCGACGTCGCGGACCTCACCGACGTGCTCGCCACGTACGACGTCGTCTT<br>CCTGGCCGCGCTCGTGGGCATGGCTGCCGAGGACAAGGCCAAGGTGATCGCACACCTTGGCGCGCGCATGGCGGACGGGGCGGCC<br>CTCGTCGTGCGCAGCGCGCACGGGGCGCGTGGGTTCCTGTACCCGATCGTCGATCCCCAGGACATCGGCCGAGGCGGGTTCGAGGT<br>GCTCGCCGTGTGTCACCCCGACGACGACGTGGTGAACTCCGTCATCATCGCACAAAAGTCCAACGACATGCATGCGAATGGACTTCGC<br>AACGGGCGTGGTGGACAGTACGCGCGGGGCACGGTGCCGGTGGTCAGCCCACCCTGCAGGTTCGGCGAGATGGTGGCAGACGTGG<br>CCCAGAAGAGAGAGGAGTTTGCCAACGCGGAAGTGGCCTTCTGA
TraesCS2A02G095700	ATGGGCGTGGAGGGCTGCTGCAACAAGAAGGTGATGGAGGAGGAGGCCCTGGTGAAGAAGATCACCGGCCTCGCCGCGGCCATCGG<br>CGAGCTGCCCTCGCTGAGCCCCTCGCCGGCGGTGAACGCGCTCTTCACCGAGCTGGTGACGTCCTGCATCCCGGCGAGCACCGTCG<br>ACGTCGACGCGCTGGGCCCGGAGGCGCAGGAGATGCGCGCCCGCCTCATCCGCCTCTGCGCCGACGCCGAGGGCCACCTGGAGGC<br>GCACTACTCCGACCTCCTCGCCGCGCACGACAACCCGCTCGACCACCTCACCCTCTTCCCCTACTTCAACAACTACATCAAGCTCAGC

	CAGCTGGAGCACGGCCTCCTCGCCCGCCACGTCCCGGGCCCGGCCCCGGCCCGCGTCGCCTTCCTCGGCTCCGGCCCGCTGCCGC<br>TCAGCTCCCTCGTGCTCGCCGCGCGCCACCTGCCCGACGCCTCCTTCGACAACTACGACATCTCCGGCGAGGCCAACGAGCGCGCC<br>AGCCGGCTGGTCCGCAACGACGCCGACGCCGGCGCGCGCATGGCGTTCCGCACGGCAGACGTGGCCGACGTGACGACCGAGCTGG<br>CGGGCTACGACGTGGTGTTCCTGGCGGCGCTGGTGGGCATGGCGGCCGAGGAGAAGGCGCGCCTGGTGGAGCACCTCGGGAGGCA<br>CATGGCCCCCGGCGCGGCGCTCGTGGTGCGGAGCGCGCACGGCGCGCGCGGGTTCCTGTACCCGGTGGTGGACCCCGAGGAGATC<br>CGGCGAGGCGGGTTCGAGGTGCTCACCGTGCACCACCCGGAGGACGAGGTGATCAACTCCGTCATCATCGCGCGCAAGGCGGCGGC<br>GCCAGCAGCCGTCGCCGCCGACGGCGACGTGCCGGTGAACAACGGCGGCATGCCGGCCCAGTGCGCGGTGGCGGTGAGCAGACC<br>GTGCCTGGGCTGCGCCTGCGAGCTGGGGACGAGGGCGCACCAGAAGATGAAGGAGATGGCCATGGAGGAGATGGAGGCGTAG
TraesCS2B0<br>2G111100	ATGGGCATGGAAGGCTGCTGCAACAAGAAGGTGATGGAGGAGGAGGCCCTGGTGAAGAAGATCACCGGCCTCGCCGCGGCCATCGG<br>CGAGCTGCCCTCGCTGACCCCGTCGCCGGCGGTGAACGCGCTCTTCACCGAGCTGGTGACGTCCTGCATCCCGGCGAGCACCGTCG<br>ACGTCGACGCGCTGGGCCCGGAGGCGCAGGAGATGCGCGCCCGCCTGATCCGCCTCTGCGCCGACGCCGAGGGCCACCTGGAGGC<br>GCACTACTCCGACCTCCTCGCCGCGCACGACAACCCGCTCGACCACCTCACCCTCTTCCCCTACTTCAACAACTACATCAAGCTCAGC<br>CAGCTGGAGCACGGCCTCCTCGCCCGCCACGTCCCGGGCCCTGCCCCGGCCCGCGTCGCCTTCCTCGGCTCCGGCCCGCTGCCGC<br>TCAGCTCCCTCGTGCTCGCCGCGCGCCACCTCCCCGACGCCTCCTTCGACAACTACGACATCTCCGGCGAGGCCAACGAGCGCGCCA<br>GCCGGCTGGTCCGCAACGACGCCGACGCCGGCGCGCGCATGGCGTTCCGCACGGCGGACGTGGCCGACGTGACCACCGAGCTGGC<br>GGGGTACGACGTGGTGTTCCTGGCGGCGCTGGTGGGCATGGCGGCCGAGGAGAAGGCGCGCCTGGTGGAGCACCTCGGGAGGCAC<br>ATGGCCCCCGGCGCGGCGCTCGTGGTGCGGAGCGCGCACGGCGCGCGCGGGTTCCTGTACCCGGTGGTGAACCCCGAGGAGATCC<br>GGCGAGGCGGGTTCGAGGTGCTCACCGTGCACCACCCGGAGGACGAGGTGATCAACTCCGTCATCATCGCGCGCAAGGCGGCCGCG<br>CCAGCAGCCGTTGCCGCCGACGGCGACGTGCCGGTGAACAGCGGCAGCATGCCGGCCCAGTGCGCGGTGGCGGTGAGCAGACCGT<br>GCCTGGGCTGCGCCTGCGAGCTGGGGGCGAGGGCGCACCAGAAGATGAAGGAGATGGCCATGGAGGAGATGGAGGCGTAG
TraesCS2D0<br>2G094200	ATGGGCATGGAGGGGTGCTGCGACAAGAAGGTGATGGAGGAGGAGGCCCTGGTGAAGAAGATCACCGGCCTCGCCGCGGCCATCGG<br>CGAGCTGCCCTCGCTGAGCCCGTCGCCGGCGGTGAACGCGCTCTTCACCGAGCTGGTGACGTCCTGCATCCCGGCGAGCACCGTGG<br>ACGTGGACGCGCTGGGCCCGGAGGCGCAGGAGATGCGCGCCCGCCTCATCCGCCTCTGCGCCGACGCCGAGGGCCACCTGGAGGC<br>GCACTACTCCGACCTCCTCGCCGCGCACGACAACCCGCTCGACCACCTCACCCTCTTCCCCTACTTCAACAACTACATCAAGCTCAGC<br>CAGCTGGAGCACGGCCTCCTCGCCCGCCACGTCCCGGGCCCCGCCCCGGCCCGCGTCGCCTTCCTCGGCTCCGGCCCGCTGCCGC<br>TCAGCTCCCTCGTGCTCGCGGCGCGCCACCTCCCCGACGCCTCCTTCGACAACTACGACATCTCCGGCGAGGCCAACGAGCGCGCC<br>AGCCGGCTCGTGCGCAACGACGCCGACGCCGGCGCGCGCATGGCGTTCCGCACCGCCGACGTGGCCGACGTGACGACCGAGCTGG<br>CGGGGTACGACGTGGTGTTCCTGGCGGCGCTGGTGGGCATGGCCGCCGAGGAGAAGGCGCGCCTGGTGGAGCACCTCGGGAGGCA<br>CATGGCCCCCGGCGCGGCGCTCGTGGTGCGGAGCGCGCACGGCGCGCGCGGCTTCCTGTACCCGGTGGTGGACCCCGAGGAGATC<br>CGGCGAGGCGGGTTCGAGGTGCTCACCGTGCACCACCCGGAGGACGAGGTGATCAACTCCGTCATCATCGCGCGCAAGGCGGCGGC<br>GCCGGCGGCCGTTGCCGCCGACGGCGACGTGCCGGTGAACAACGGCGGCATGCCGGCGCAGTGCGCGGTGGCGGTGAGCAGACC<br>GTGCCTCGGCTGCGCCTGCGAGCTGGGGACGAGGGCGCACCAGAAGATGAAGGAGATGGCCATGGAGGAGATGGAGGCGTAG

Zm00001eb<br>014680	ATGGAGGCCCAGAACGTGGAGGTTGCTGCCCTGGTGAAGAAGATCGCGGACCTCCACGCCGACATCACCAAGCTGCCGTCGCTGAGC<br>CCGTCCCCCGACGTCAACGCGCTATTCACCAGCCTCGTCATGGCCTGCGTCCCGCCGAGCACTGTCGACGTGACCAAGCTCAGCCCG<br>GACTCCCAGAGGATGCGGGAGGAGCTCATTCGCCTCTGCTCCGACGCCGAGGGCCACCTCGAGGCGCACTACGCCGACATGCTGGC<br>CGCCTTCGACAACCCGCTCGACCATCTCGGCCGCTTCCCATACTTTAGCAACTACATCAACCTGAGCAAGCTGGAGTACGACCTCCTGG<br>TCCGCTACATCCCGGGTCTCGCGCCTTCCCGCGTCGCCTTCGTCGGGTCAGGCCCCTTGCCGTTCAGCTCGCTCGTGCTCGCCGCCC<br>GCCACCTGCCCAACACGACATTCGACAACTATGACCGGTGCGCTGCCGCCAACGACCGTGCGCGGAAGCTGGTCCGCGCGGACAAG<br>GACCTGAACGCGCGCATGTCCTTCCACACGGTGGACGTAGCCAACCTGACGGATGACCTCGGCAAGTACGACGTCGTCTTCCTTGCC<br>GCGCTCGTCGGCATGGCGGCCGAGGACAAGGCCAAGGTGGTCGTGCACCTTGGCAGGCACATGGCGGACGGAGCGGCTCTCGTCG<br>TGCGGAGCGCGCACGGGGCTCGCGGGTTCCTATACCCGATCGTGGATCCCGAGGACATCCGTCGTGGCGGGTTTGACGTTCTGACGG<br>TGTACCACCCGGACGATGAGGTGATCAACTCTGTCATCATCGCGCGCAAGATCGACGCCCATGCAAACACGGAGGTTTCTGCCCTGGT<br>GCAGAAGATAACCGGCCTCCACGCCGCCATCAACAAGCTGCCATCGCTGAGCCCATCCCCCGACGTCGACGCGCTGTTCACCGAGCT<br>CGTCATGGCCTGCGTCCCACCGAGCCCCGTCGACGTGACCAAGCTCGGCACGGACGCGCAGAGGATGCGGGAGGAGCTCATCCGCC<br>TCTGCTCCGACGCCGAGGGCCACCTCGAGGCGCACTATGCCGACATGCTCGCCGCCTTCGACAACCCGCTCGACCATCTCGGCCGCT<br>TCCCTTACTTCAACAACTACGTCAACCTGAGCAAGTTGGAGTACGACCTCCTGGTTCGCTATGTCCCGGGCATCGCACCTTCCAGAATC<br>GCCTTCGTCGGGTCCGGACCCCTGCCGTTCAGCTCGCTCGTGCTCGCCTCCCGCCACCTGCCGAACGTGATGTTCGACAACTACGAC<br>CGGTGCGCCGCCGCCAACGACCGTGCACGGAAGCTGGTCCGCGCGGACGAGGGTCTGCGCAAGCGAATGTTCTTCCACACCGCCGA<br>CGTCGCCAACCTCACGGACGAGCTCCGCAAGTACGACGTGGTCTTCCTGGCCGCGCTCGTCGGCATGGCGGCCGAGGACAAGGCCA<br>AGGTGGCTACGCACCTTGGCAGACACATGGCGGATGGAGCGGCTCTCATCGTGCGGAGCGCCCACGAGGCTCGAGGGTTCCTGTACC<br>CGATCGTCGATCCCGAGGACATCCGTCGCAGCGGCTTCGACGTGCTTGCCGTGTACCACCCGGACGACGAAGTCATTAACTCCGTCAT<br>CGTCGCGCGCAAGATCAACGCCCACGTGAAGGGGCTTCAGGATGGACACGCGCATGCGCGCGGTGTGGTGCCGATAGTGAGCCCGC<br>CATGCAAGTGCTGCAAGATGGAGGCGAACACGCTCCACCAAAAGAGGGAGGAGATGGCGACGGCGTAG
Zm00001eb<br>396250	ATGGAGGCCCAGAACGTGGAGGTTGCTGCCCTGGTGCAGAAGATCGCGGCCCTCCACGCCGACATCGCCAAGCTGCCGTCGCTGAG<br>CCCGTCCCCCGACGCCAACGCGCTGTTCACCAGCCTCGTCATGGCCTGCGTCCCGCCAAACCCTGTCGACGTGACCAAGCTCAGCCC<br>GGACGTCCAGGGGATGCGAGAGGAGCTCATCCGCCTCTGCTCCGACGTCGAGGGCCACCTCGAGGCGCACTACGCCGACATGCTCG<br>CCGCCTTCGACAACCCGCTCGACCACCTCGGCCGCTTTCCCTACTTCAGCAACTACATCGACCTGAGCAAGCTGGAGTTTGACCTCCT<br>TGTCCGCTACATCCCAGGGCTCGCGCCTTCCCGTGTCGCCTTCGTCGGCTCCGGCCCCTTGCCGTTCAGCTCGCTCGTGCTCGCCGC<br>GCGCCACCTGCCCAACACACTGTTCGACAACTACGATCGGTGTGCCGCCGCCAACGACCGTGCTCGGAAGCTGGTCCGTGCGGACAA<br>GGACCTGAACGCGCGCATGTCCTTCCACACGGTCGACGTCGCCAACCTCACGGACGAGCTCGCCAAGTACGAC
Zm00001eb<br>218440	ATGGTCGTCATGGGCAAGCACGATGACGATGAGAAGCAGCAGCAGCAGATGGAGATGGAAGACGTACATGAAGTAGGAGCCGTCGAG<br>GTGCTGCCGCTGGAGAATGAGGCGGAGGCCGCGCTGGTGCGCAAGATCTCCGGCCTGGCCGCCGCCATCGCGAAGCTGCCGTCGCT<br>GAGCCCGTCGCCGGAGGTGAACGCGCTGTTCACGGACCTCGTCACGGCCTGCATCCCGCGCAGCACCGTGGACGTGGAACGCCTGG<br>GCCCGGAGCTGCAGCGCATGCGCGCGGGCCTCATCCGCCGCTGCGCCGACGCCGAGGCCCAGCTGGAGGCGCACTACTCGGACCT<br>GCTGGCGGCCTTGGACAGCCCGCTGGACCACCTGCCGCTCTTCCCCTACTTCGGCAACTACCTGCTGCTGGGCGAGCTGGAGCACG

	GCCTGCTGGCGCGCCACGTCCCGGGCCCGCCGCCCGCCCGCGTCGCCTTCGTGGGGTCCGGCCCGCTCCCGCTCAGCTCCCTCGT<br>GCTCGCCGCGCGCCACCTCCCCGCCGCCGCCTTCGACAACTACGACATCTGCGCGGAGGCCAACGGCCGGGCGCGCCGCCTCGTG<br>CGCGCCGACGCCGCGCTCGCCGCGCGCATGGCGTTCCGCACCTCCGACGTGGCCCACGTCACGCGGGGCCTGGCCACCTACGACG<br>TCGTCTTCCTGGCGGCGCTCGTGGGCGTGGCGGCCGAGGAGAAGGCGCGCGTGGTGGAGCACCTCGGGAGGCACATGGCGCCCGG<br>CGCCGCGCTGGTGGTGCGCAGCGCGCACGGCGCGCGCGCCTTCCTGTACCCCGTCGTCGACCCAGACGAGATCCGCCGCGGCGGC<br>TTCGACGTGCTCGCCGTGCACCACCCGGAGGGGGAGGTGATCAACTCCGTCATCGTCGCGCGTAAGCCCGTCCCCGTCGCCGGAGT<br>GGGGCACGCGCACGCGCACGCCCACGGGCACGGCGCCGTGCTCAGCCGGCCCTGCTGCCTCTGCTGCGAGATGGAGGCCCGGACG<br>CAC

Supplementary Table S4. Primer sequences used in OE-*TaNAS* transgene characterization. Detection of the transgene used the *hptII* primer pair and the *TaCyclophillin* primer pair as a housekeeping control. Amplification and sequencing of the *TaNAS* transgenes used a primer pair in the ubiquitin promoter 1 (ubi1) and nopaline synthase terminator (nosT). Quantification of *TaNAS* transgene expression used the *attB2-nosT* primer pair normalised to three housekeeping genes: *TaACT*, *TaGPDH*, and *TaSAR*.

**Gene target**	**Forward primer (5'-3')**	**Reverse primer (5'-3')**
hptII	CTCGGAGGGCGAAGAATCTC	GCGGGAGATGCAATAGGTCA
TaCyclophilin	CAAGCCGCTGCACTACAAGG	AGGGGACGGTGCAGATGAA
ubi1-nosT	TTTAGCCCTGCCTTCATACG	AATGTTTGAACGATCGGGGAA
attB2-nosT	CCGACCCAGCTTTCTTGTAC	TGTTTGAACGATCGGGGAAA
TaACT	GACAATGGAACCGGAATGGTC	GTGTGATGCCAGATTTTCTCCAT
TaGAPDH	TTCAACATCATTCCAAGCACCA	CGTAACCCAAAATGCCCTTG
TaSAR	GAGTCTGCCCACCCATTCGTAA	GACATGCCATAGGTTTCAGCGAC

Supplementary Table S5. Haplotypes of *TaNAS* genes in 11 different bread wheat cultivars and *Triticum spelta* L. *TaNAS* genes present in cvs. Chinese Spring, ArinaLrFor, Julius, Lance, Norin61, SY Mattis, Mace, *Triticum spelta* L., Jagger, Landmark, and Stanley are in first section of the table (rows 1-137). *TaNAS* genes not present in cv. Chinese Spring, but present in other cultivars are in the second section of the table (rows 138-154). Gaps in cells indicate that the *TaNAS* gene was not identified. Chromosomal locations were supplied where gene models were not available. The gene models for cv. Chinese Spring used IWGSC RefSeq2.1. Gene and protein variations between the 11 different bread wheat cultivars and *Triticum spelta* L. with Chinese Spring are indicated except for *TaNAS3-B1* and *TaNAS6-A1* where the cv. Chinese Spring *TaNAS* gene variation is unique relative to all other cultivars. The *TaNAS2-B3*, *TaNAS4-A3*, *TaNAS5-B3*, and *TaNAS8-B2* genes are not present in cv. Chinese Spring and variations are compared to the most common haplotype (H). Nucleotide (nt), Single Nucleotide Polymorphisms (SNPs), conserved (cv) and non-conserved (ncs) amino acid (aa) substitutions (sub), replacements (rep), extensions (ext), truncations (trun), deletions (del), insertions (ins) at the N-terminus (N-ter), and C-terminus (C-ter) are indicated.

**Gene**	**Chinese Spring RefSeq2.1**	**ArinaLrFor**	**Julius**	**Lancer**	**Norin61**	**SY Mattis**	**Mace**	**Triticum spelta**	**Jagger**	**Landmark**	**Stanley**	**Fielder**
**TaNAS1-A1**	**TraesCS2A03G066300**	**TraesARI2A03G00595190**	**TraesJUL2A03G00591680**	**TraesLAC2A03G00592550**	**TraesNOR2A03G00596190**	**TraesSYM2A03G00595200**	**TraesMAC2A03G00587670**	**TraesTSP2A01G037400**	**TraesJAG2A03G00587010**	**TraesLDM2A03G00590820**	**TraesSTA2A03G00587780**	**2A:14977723-14976881 Reverse**
Gene	-	100% identical	50→83 nt rep (3')	100% identical	100% identical	57 SNPs	57 SNPs	46 SNPs	57 SNPs	100% identical	57 SNPs	100% identical
Protein	-	-	16→27 aa rep (C-ter)	-	-	9 cs & 6 ncs aa subs, 6 aa ext (C-ter)	9 cs & 6 ncs aa subs, 6 aa ext (C-ter)	4 cs & 8 ncs aa subs	9 cs & 6 ncs aa subs, 6 aa ext (C-ter)	-	9 cs & 6 ncs aa subs, 6 aa ext (C-ter)	-
Haplotype	H1	H1	H4	H1	H1	H2	H2	H3	H2	H1	H2	H1
**TaNAS1-B1**	**TraesCS2B03G098000**	**TraesARI2B03G00842250**	**TraesJUL2B03G00837550**	**TraesLAC2B03G00829000**	**TraesNOR2B03G00843360**	**TraesSYM2B03G00842670**	**TraesMAC2B03G00830370**	**TraesTSP2B01G050200**	**TraesJAG2B03G00832430**	**TraesLDM2B03G00834640**	**TraesSTA2B03G00833000**	**2B:23550688-23549857 Reverse**
Gene	-	100% identical	100% identical	100% identical	100% identical	4 SNPs	100% identical	3 nt del	100% identical	100% identical	100% identical	100% identical
Protein	-	-	-	-	-	1 cs aa sub	-	1 aa del	-	-	-	-
Haplotype	H1	H1	H1	H1	H1	H2	H1	H3	H1	H1	H1	H1
**TaNAS1-D1**	**TraesCS2D03G061000**	**TraesARI2D03G01105860**	**TraesJUL2D03G01095670**	**TraesLAC2D03G01042340**	**TraesNOR2D03G01105730**	**TraesSYM2D03G01104740**	**TraesMAC2D03G01089010**	**TraesTSP2D01G038800**	**TraesJAG2D03G01093810**	**TraesLDM2D03G01091450**	**TraesSTA2D03G01078740**	**2D:12872919-12872085 Reverse**

Gene	-	59→26 nt rep (3')	23 SNPs	59→26 nt rep (3')	59→26 nt rep (3')	59→26 nt rep (3')	59→26 nt rep (3')	100% identical	59→26 nt rep (3')	59→26 nt rep (3')	59→26 nt rep (3')	59→26 nt rep (3')
Protein	-	19→8 aa rep (C-ter)	2 cs & 4 ncs aa subs	19→8 aa rep (C-ter)	19→8 aa rep (C-ter)	19→8 aa rep (C-ter)	19→8 aa rep (C-ter)	-	19→8 aa rep (C-ter)	19→8 aa rep (C-ter)	19→8 aa rep (C-ter)	19→8 aa rep (C-ter)
Haplotype	H2	H1	H3	H1	H1	H1	H1	H2	H1	H1	H1	H1
**TaNAS2-A1**	**TraesCS6A03G395600**	**TraesARI6A03G03251690**	**TraesJUL6A03G03322480**	**TraesLAC6A03G03251150**	**TraesNOR6A03G03327840**	**TraesSYM6A03G03237010**	**TraesMAC6A03G03295720**	**TraesTSP6A01G136800**	**TraesJAG6A03G03290620**	**TraesLDM6A03G03299590**	**TraesSTA6A03G03287000**	**6A:158317844-158316864 Reverse**
Gene	-	100% identical	100% identical	100% identical	100% identical	100% identical	100% identical	100% identical	100% identical	100% identical	100% identical	100% identical
Protein	-	-	-	-	-	-	-	-	-	-	-	-
Haplotype	H1	H1	H1	H1	H1	H1	H1	H1	H1	H1	H1	H1
**TaNAS2-A2**	**TraesCS6A03G396300**	**TraesARI6A03G03251820**	**TraesJUL6A03G03322590**	**TraesLAC6A03G03251260**	**TraesNOR6A03G03327970**	**TraesSYM6A03G03237130**	**TraesMAC6A03G03295840**	**TraesTSP6A01G137000**	**TraesJAG6A03G03290740**	**TraesLDM6A03G03299720**	**TraesSTA6A03G03287100**	**6A:158454932-158448655 Reverse**
Gene	-	30 nt ins, 2 SNPs	33 nt ins, 1 SNP	30 nt ins, 1 SNP	33 nt ins, 1 SNP	30 nt ins, 1 SNP	30 nt ins, 2 SNPs	1 SNP	33 nt ins, 1 SNP	2 SNPs	2 SNPs	1 SNP
Protein	-	10 aa insert, 1 ncs aa sub	11 aa ins, 1 ncs aa sub	10 aa ins, 1 ncs aa sub	11 aa ins, 1 ncs aa sub	10 aa ins, 1 ncs aa sub	10 aa ins, 1 ncs aa sub	1 ncs aa sub	10 aa ins, 1 ncs aa sub	1 ncs sub	1 ncs sub	1 ncs aa sub
Haplotype	H7	H4	H6	H3	H5	H3	H4	H2	H5	H1	H1	H2
**TaNAS2-A3**	**TraesCS6A03G404500**	**TraesARI6A03G03253210**	**TraesJUL6A03G03323910**	**TraesLAC6A03G03252550**	**TraesNOR6A03G03329190**	**TraesSYM6A03G03238460**	**TraesMAC6A03G03297120**	**TraesTSP6A01G139100**	**TraesJAG6A03G03291990**	**TraesLDM6A03G03301050**	**TraesSTA6A03G03288370**	**6A:162891463-162890474 Reverse**
Gene	-	100% identical	100% identical	100% identical	100% identical	100% identical	100% identical	100% identical	100% identical	100% identical	100% identical	100% identical
Protein	-	-	-	-	-	-	-	-	-	-	-	-
Haplotype	H1	H1	H1	H1	H1	H1	H1	H1	H1	H1	H1	H1
**TaNAS2-B1**	**TraesCS6B03G477500**	**TraesARI6B03G03457020**	**TraesJUL6B03G03520670**	**TraesLAC6B03G03452760**	**TraesNOR6B03G03532210**	**TraesSYM6B03G03436680**	**TraesMAC6B03G03496990**	**TraesTSP6B01G203600**	**TraesJAG6B03G03488970**	**TraesLDM6B03G03502950**	**TraesSTA6B03G03489330**	**6B:213488328-213487339 Reverse**

Gene	-	100% identical	100% identical	1 SNP	100% identical	100% identical	1 SNP	100% identical	23 SNPs	100% identical	23 SNPs	100% identical
Protein	-	-	-	Identical	-	-	Identical	-	3 cs & 3 ncs subs	-	3 cs & 3 ncs subs	-
Haplotype	H1	H1	H1	H2	H1	H1	H2	H1	H3	H1	H3	H1
**TaNAS2-B2**	**TraesCS6B03G474000**	**TraesARI6B03G03456420**	**TraesJUL6B03G03521300**	**TraesLAC6B03G03452140**	**TraesNOR6B03G03531560**	**TraesSYM6B03G03437300**	**TraesMAC6B03G03496390**	**TraesTSP6B01G202200**	**TraesJAG6B03G03488340**	**TraesLDM6B03G03502410**	**TraesSTA6B03G03488680**	**6B:212159685-212158696 Reverse**
Gene	-	100% identical	6 SNPs	100% identical	100% identical	6 SNPs	100% identical	100% identical	12 SNPs	100% identical	12 SNPs	2 SNPs
Protein	-	-	1 cs & 1 ncs aa sub	-	-	1 cs & 1 ncs aa sub	-	-	2 cs & 3 ncs aa subs	-	2 cs & 3 ncs aa subs	1 ncs aa sub
Haplotype	H1	H1	H3	H1	H1	H3	H1	H1	H4	H1	H4	H2
**TaNAS2-D1**	**TraesCS6D03G330700**	**TraesARI6D03G03658000**	**TraesJUL6D03G03726950**	**TraesLAC6D03G03644720**	**TraesNOR6D03G03734690**	**TraesSYM6D03G03641330**	**TraesMAC6D03G03692730**	**TraesTSP6D01G156200**	**TraesJAG6D03G03677370**	**TraesLDM6D03G03697920**	**TraesSTA6D03G03688240**	**6D:121225558-121226547 Forward**
Gene	-	100% identical	100% identical	100% identical	100% identical	100% identical	100% identical	100% identical	100% identical	100% identical	100% identical	100% identical
Protein	-	-	-	-	-	-	-	-	-	-	-	-
Haplotype	H1	H1	H1	H1	H1	H1	H1	H1	H1	H1	H1	H1
**TaNAS2-D2**	**TraesCS6D03G331700**	**TraesARI6D03G03658210**	**TraesJUL6D03G03727150**	**TraesLAC6D03G03644920**	**TraesNOR6D03G03734910**	**TraesSYM6D03G03641550**	**TraesMAC6D03G03692940**	**TraesTSP6D01G156600**	**TraesJAG6D03G03677580**	**TraesLDM6D03G03698140**	**TraesSTA6D03G03688470**	**6D:121580460-121579471 Reverse**
Gene	-	100% identical	100% identical	100% identical	100% identical	100% identical	100% identical	100% identical	100% identical	100% identical	100% identical	100% identical
Protein	-	-	-	-	-	-	-	-	-	-	-	-
Haplotype	H1	H1	H1	H1	H1	H1	H1	H1	H1	H1	H1	H1
**TaNAS2-D3**	**TraesCS6D03G330000**	**TraesARI6D03G03657880**	**TraesJUL6D03G03726840**	**TraesLAC6D03G03644600**	**TraesNOR6D03G03734570**	**TraesSYM6D03G03641210**	**TraesMAC6D03G03692600**	**TraesTSP6D01G156000**	**TraesJAG6D03G03677250**	**TraesLDM6D03G03697800**	**TraesSTA6D03G03688120**	**6D:121064488-121065477 Forward**

Gene	-	100% identical	100% identical	100% identical	100% identical	100% identical	100% identical	100% identical	100% identical	100% identical	100% identical	100% identical
Protein	-	-	-	-	-	-	-	-	-	-	-	-
Haplotype	H1	H1	H1	H1	H1	H1	H1	H1	H1	H1	H1	H1
**TaNAS3-A1**	**TraesCS2A03G093200**	**TraesARI2A03G00598990**	**TraesJUL2A03G00595510**	**TraesLAC2A03G00596440**	**TraesNOR2A03G00600070**	**TraesSYM2A03G00599320**	**TraesMAC2A03G00591870**	**TraesTSP2A01G052900**	**TraesJAG2A03G00591190**	**TraesLDM2A03G00594610**	**TraesSTA2A03G00591890**	**2A:19164152-19163160 Reverse**
Gene	-	100% identical	100% identical	100% identical	100% identical	42 SNPs	42 SNPs	22 SNPs	42 SNPs	100% identical	42 SNPs	100% identical
Protein	-	-	-	-	-	7 cs & 7 ncs aa subs	7 cs & 7 ncs aa subs	1 cs & 2 ncs aa subs	7 cs & 7 ncs aa subs	-	7 cs & 7 ncs aa subs	-
Haplotype	H1	H1	H1	H1	H1	H2	H2	H3	H2	H1	H2	H1
**TaNAS3-B1**	**TraesCS2B03G130700**	**TraesARI2B03G00849120**	**TraesJUL2B03G00841820**	**TraesLAC2B03G00833920**	**TraesNOR2B03G00848160**	**TraesSYM2B03G00847200**	**TraesMAC2B03G00835170**	**TraesTSP2B01G068000**	**TraesJAG2B03G00837080**	**TraesLDM2B03G00839040**	**TraesSTA2B03G00837720**	**2B:29119026-29120018 Forward**
Gene	2 SNPs	100% identical	100% identical	100% identical	100% identical	100% identical	100% identical	100% identical	100% identical	100% identical	100% identical	100% identical
Protein	Identical	-	-	-	-	-	-	-	-	-	-	-
Haplotype	H2	H1	H1	H1	H1	H1	H1	H1	H1	H1	H1	H1
**TaNAS3-D1**	**TraesCS2D03G092200**	**TraesARI2D03G01109930**	**TraesJUL2D03G01099970**	**TraesLAC2D03G01045510**	**TraesNOR2D03G01109800**	**TraesSYM2D03G01108480**	**TraesMAC2D03G01092650**	**TraesTSP2D01G057400**	**TraesJAG2D03G01097390**	**TraesLDM2D03G01095590**	**TraesSTA2D03G01082660**	**2D:18169945-18168953 Reverse**
Gene	-	12 SNPs	100% identical	12 SNPs	100% identical	12 SNPs	12 SNPs	10 SNPs	12 SNPs	100% identical	100% identical	3 SNPs
Protein	-	-	-	3 cs & 3 ncs aa subs	-	3 cs & 3 ncs aa subs	3 cs & 3 ncs aa subs	3 cs & 3 cns aa subs	3 cs & 3 ncs aa subs	-	-	1 cs aa sub
Haplotype	H1	H4	H1	H4	H1	H4	H4	H3	H4	H1	H1	H2
**TaNAS4-A1**	**TraesCS5A03G1289600**	**TraesARI5A03G02826930**	**TraesJUL5A03G02803850**	**TraesLAC5A03G02740020**	**TraesNOR5A03G02809500**	**TraesSYM5A03G02817800**	**TraesMAC5A03G02783860**	**TraesTSP5A01G589500**	**TraesJAG5A03G02786450**	**TraesLDM5A03G02788100**	**TraesSTA5A03G02776250**	**5A:705403281-705402280 Reverse**

Gene	-	53 SNPs	100% identical	100% identical	100% identical	53 SNPs	100% identical	100% identical	1 SNP→24 nt ext (3')	1 SNP→24 nt ext (3')	1 SNP→24 nt ext (3')	1 SNP
Protein	-	7 ncs subs	-	-	-	7 ncs subs	-	-	8 aa ext (C-ter)	8 aa ext (C-ter)	8 aa ext (C-ter)	1 ncs aa sub
Haplotype	H1	H4	H1	H1	H1	H4	H1	H1	H3	H3	H3	H2
**TaNAS4-A2**	**TraesCS5A03G1289100**	**TraesARI5A03G02826980**	**TraesJUL5A03G02803790**	**TraesLAC5A03G02740080**	**TraesNOR5A03G02809440**	**TraesSYM5A03G02817780**	**TraesMAC5A03G02783780**	**TraesTSP5A01G589900**	**TraesJAG5A03G02786390**	**TraesLDM5A03G02788040**	**TraesSTA5A03G02776180**	**5A:705349537-705348536 Reverse**
Gene	-	27 SNPs	100% identical	100% identical	100% identical	27 SNPs	100% identical	100% identical	100% identical	100% identical	100% identical	2 SNPs
Protein	-	3 cs & 4 ncs aa subs	-	-	-	3 cs & 4 ncs aa subs	-	-	-	-	-	Identical
Haplotype	H1	H3	H1	H1	H1	H3	H1	H1	H1	H1	H1	H2
**TaNAS4-D1**	**4D:513216136-513217137**	**TraesARIUn03G04594360**	**TraesJULUn03G04549510**	**TraesLACUn03G04460380**	**TraesNORUn03G04558740**	**scaffold_corrected_130-1:4924198-4925199**	**TraesMACUn03G04511200**	**TraesTSP4D01G404100**	**TraesJAG1D03G00574960**	**TraesLDM6B03G03624440**	**TraesSTAUn03G04532820**	**Un:108614518-108613517 Reverse**
Gene	-	27 SNPs	27 SNPs	27 SNPs	100% identical	27 SNPs	27 SNPs	28 SNPs	27 SNPs	27 SNPs	27 SNPs	100% identical
Protein	-	3 cs & 6 ncs aa subs	3 cs & 6 ncs aa subs	3 cs & 6 ncs aa subs	-	3 cs & 6 ncs aa subs	3 cs & 6 ncs aa subs	3 cs & 7 ncs aa subs	3 cs & 6 ncs aa subs	3 cs & 6 ncs aa subs	3 cs & 6 ncs aa subs	-
Haplotype	H2	H1	H1	H1	H2	H1	H1	H3	H1	H1	H1	H2
**TaNAS4-D2**	**4D:513233756-513234757**	**TraesARIUn03G04594330**	**TraesJULUn03G04549540**	**TraesLACUn03G04460410**	**TraesNORUn03G04558770**	**scaffold_corrected_130-1:4906770-4907771**	**TraesMACUn03G04511170**	**TraesTSP4D01G404400**	**TraesJAG1D03G00574990**	**TraesLDM6B03G03624480**	**TraesSTAUn03G04532850**	**Un:108595897-108596898 Forward**
Gene		31 SNPs	31 SNPs	31 SNPs	100% identical	31 SNPs	31 SNPs	31 SNPs	31 SNPs	31 SNPs	31 SNPs	8 SNPs
Protein		1 cs & 3 ncs aa subs	1 cs & 3 ncs aa subs	1 cs & 3 ncs aa subs		1 cs & 3 ncs aa subs	1 cs & 3 ncs aa subs	1 cs & 3 ncs aa subs	1 cs & 3 ncs aa subs	1 cs & 3 ncs aa subs	1 cs & 3 ncs aa subs	1 cs & 2 ncs aa subs

Haplotype	H2	H1	H1	H1	H2	H1	H1	H1	H1	H1	H1	H3
**TaNAS5-B1**	**TraesCS3B03G154800**	**TraesARI3B03G01580550**	**TraesJUL3B03G01569870**	**TraesLAC3B03G01499150**	**TraesNOR3B03G01579440**	**TraesSYM3B03G01580660**	**TraesMAC3B03G01556940**	**TraesTSP3B01G085300**	**TraesJAG3B03G01566210**	**TraesLDM3B03G01558270**	**TraesSTA3B03G01550190**	**3B:40773334-40774332 Forward**
Gene	-	456 nt ext (5')	456 nt ext (5')	456 nt ext (5')	456 nt ext (5')	456 nt ext (5')	456 nt ext (5')	100% identical	456 nt ext (5')	456 nt ext (5'), 1 SNP	456 nt ext (5'), 1 SNP	456 nt ext (5')
Protein	-	152 aa ext (N-ter)	152 aa ext (N-ter)	152 aa ext (N-ter)	152 aa ext (N-ter)	152 aa ext (N-ter)	152 aa ext (N-ter)	-	152 aa ext (N-ter)	152 aa ext (N-ter)	152 aa ext (N-ter)	152 aa ext (N-ter)
Haplotype	H2	H1	H1	H1	H1	H1	H1	H2	H1	H3	H3	H1
**TaNAS5-B2**	**TraesCS3B03G0154700**	**TraesARI3B03G01580480**	**TraesJUL3B03G01569780**	**TraesLAC3B03G01499070**	**TraesNOR3B03G01579350**	**TraesSYM3B03G01580590**	**TraesMAC3B03G01556850**	**TraesTSP3B01G085100**	**TraesJAG3B03G01566110**	**TraesLDM3B03G01558180**	**TraesSTA3B03G01550090**	**3B:40535199-40535741 Forward**
Gene	-	1 SNP	1 SNP	1 SNP	1 SNP	1 SNP	1 SNP	1 SNP	1 SNP	16 SNPs & 57 nt ext (3')	16 SNPs & 57 nt ext (3')	1 SNP
Protein	-	1 ncs aa sub	1 ncs aa sub	1 ncs aa sub	1 ncs aa sub	1 ncs aa sub	1 ncs aa sub	1 ncs aa sub	1 ncs aa sub	8 ncs subs, 19 aa ext (C-ter)	8 ncs subs, 19 aa ext (C-ter)	1 ncs aa sub
Haplotype	H2	H1	H1	H1	H1	H1	H1	H1	H1	H3	H3	H1
**TaNAS6-A1**	**TraesCS4A03G249100LC**	**TraesARI4A03G02082110**	**TraesJUL4A03G02065240**	**TraesLAC4A03G02000050**	**TraesNOR4A03G02073110**	**TraesSYM4A03G02072240**	**TraesMAC4A03G02046010**	**4A:148417599-148419883**	**TraesJAG4A03G02053440**	**TraesLDM4A03G02045130**	**TraesSTA4A03G02042380**	**4A:149046701-149047678 Forward**
Gene	348 nt trun (5')	100% identical	100% identical	1 SNP	140→95 nt sub (3')	100% identical	140→95 nt sub (3')	100% identical	140→95 nt sub (3')	100% identical	140→95 nt sub (3')	100% identical
Protein	116 aa trun (N-ter)	-	-	1 ncs aa sub	46→31 aa (C-ter)	-	1 ncs aa sub, 46→31 aa (C-ter)	-	46→31 aa (C-ter)	-	46→31 aa (C-ter)	-
Haplotype	H5	H1	H1	H2	H3	H1	H4	H1	H3	H1	H3	H1
**TaNAS6-B1**	**TraesCS4B03G520200**	**TraesARI4B03G02364430**	**TraesJUL4B03G02345670**	**TraesLAC4B03G02280440**	**TraesNOR4B03G02343820**	**TraesSYM4B03G02353360**	**TraesMAC4B03G02326520**	**TraesTSP4B01G198800**	**TraesJAG4B03G02324730**	**TraesLDM4B03G02327870**	**TraesSTA4B03G02322000**	**4B:403254329-403253337 Reverse**

Gene	-	100% identical	100% identical	100% identical	100% identical	100% identical	100% identical	100% identical	100% identical	100% identical	100% identical	100% identical
Protein	-	-	-	-	-	-	-	-	-	-	-	-
Haplotype	H1	H1	H1	H1	H1	H1	H1	H1	H1	H1	H1	H1
**TaNAS6-D1**	**TraesCS4D03G458900**	**TraesARI4D03G02539330**	**TraesJUL4D03G02519460**	**TraesLAC4D03G02453710**	**TraesNOR4D03G02517730**	**TraesSYM4D03G02528210**	**TraesMAC4D03G02498320**	**TraesTSP4D01G198500**	**TraesJAG4D03G02497690**	**TraesLDM4D03G02502750**	**TraesSTA4D03G02495500**	**4D:323098111-323096957 Reverse**
Gene	-	162 nt trun (5')	162 nt trun (5')	162 nt trun (5')	162 nt trun (5')	162 nt trun (5')	162 nt trun (5')	100% identical	162 nt trun (5')	162 nt trun (5')	162 nt trun (5')	162 nt trun (5')
Protein	-	54 aa trun (N-ter)	54 aa trun (N-ter)	54 aa trun (N-ter)	54 aa trun (N-ter)	54 aa trun (N-ter)	54 aa trun (N-ter)	-	54 aa trun (N-ter)	54 aa trun (N-ter)	54 aa trun (N-ter)	54 aa trun (N-ter)
Haplotype	H2	H1	H1	H1	H1	H1	H1	H2	H1	H1	H1	H1
**TaNAS7-A1**	**TraesCS6A03G214000**	**TraesARI6A03G03221180**	**TraesJUL6A03G03292100**	**TraesLAC6A03G03220000**	**TraesNOR6A03G03297080**	**TraesSYM6A03G03206290**	**TraesMAC6A03G03265460**	**-**	**TraesJAGUn03G04499290**	**TraesLDM6A03G03269390**	**TraesSTA6A03G03256200**	**6A:60973173-60972181 Reverse**
Gene	-	2 SNPs	2 SNPs	100% identical	100% identical	2 SNPs	100% identical	-	100% identical	100% identical	100% identical	100% identical
Protein	-	1 cs aa sub	1 cs aa sub	-	-	1 cs aa sub	-	-	-	-	-	-
Haplotype	H1	H2	H2	H1	H1	H2	H1	-	H1	H1	H1	H1
**TaNAS7-A2**	**TraesCS6A03G973000**	**TraesARI6A03G03362910**	**TraesJUL6A03G03432260**	**TraesLAC6A03G03362370**	**TraesNOR6A03G03439190**	**TraesSYM6A03G03348250**	**TraesMAC6A03G03404280**	**TraesTSP6A01G374500**	**TraesJAG6A03G03398040**	**TraesLDM6A03G03408800**	**TraesSTA6A03G03395830**	**6A:603249197-603250187 Forward**
Gene	-	100% identical	100% identical	17 SNPs	100% identical	100% identical	100% identical	2 SNPs	100% identical	100% identical	100% identical	16 SNPs
Protein	-	-	-	5 cs & 1 ncs aa sub	-	-	-	1 ncs aa sub	-	-	-	5 cs & 1 ncs sub
Haplotype	H1	H1	H1	H4	H1	H1	H1	H2	H1	H1	H1	H3
**TaNAS7-B1**	**TraesCS6B03G1190400**	**TraesARI6B03G03586430**	**TraesJUL6B03G03656530**	**TraesLAC6B03G03580460**	**TraesNOR6B03G03662980**	**TraesSYM6B03G03568520**	**TraesMAC6B03G03626210**	**TraesTSP6B01G462100**	**TraesJAG6B03G03616290**	**TraesLDM6B03G03626760**	**TraesSTA6B03G03614750**	**6B:694258986-694259978 Forward**

Gene	-	7 SNPs	5 SNPs	43 SNPs	5 SNPs	5 SNPs	43 SNPs	43 SNPs	5 SNPs	5 SNPs	100% identical	5 SNPs
Protein	-	1 cs & 1 ncs aa sub	1 ncs aa sub	7 cs & 10 ncs aa subs	1 ncs aa sub	1 ncs aa sub	7 cs & 10 ncs aa subs	7 cs & 10 ncs aa subs	1 ncs aa sub	1 ncs aa sub	-	1 ncs aa sub
Haplotype	H3	H4	H1	H2	H1	H1	H2	H2	H1	H1	H3	H1
**TaNAS7-D1**	**TraesCS6D03G854100**	**TraesARI6D03G03755720**	**TraesJUL6D03G03824340**	**TraesLAC6D03G03741940**	**TraesNOR6D03G03831960**	**TraesSYM6D03G03739130**	**TraesMAC6D03G03789530**	**TraesTSP6D01G398500**	**TraesJAG6D03G03774220**	**TraesLDM6D03G03795250**	**TraesSTA6D03G03784240**	**6D:456540534-456541526 Forward**
Gene	-	100% identical	100% identical	4 SNPs	100% identical	4 SNPs	100% identical	1 SNP	100% identical	100% identical	100% identical	4 SNPs
Protein	-	-	-	1 cs & 2 ncs aa subs	-	1 cs & 2 ncs aa subs	-	Identical	-	-	-	1 cs & 2 ncs aa subs
Haplotype	H1	H1	H1	H2	H1	H2	H1	H3	H1	H1	H1	H2
**TaNAS8-A1**	**TraesCS6A03G1000600**	**TraesARI6A03G03366660**	**TraesJUL6A03G03435950**	**TraesLAC6A03G03365270**	**TraesNOR6A03G03443150**	**TraesSYM6A03G03352220**	**TraesMAC6A03G03408270**	**TraesTSP6A01G387500**	**TraesJAG6A03G03401930**	**TraesLDM6A03G03412690**	**TraesSTA6A03G03399820**	**6A:609149647-609148661 Reverse**
Gene	-	16 SNPs	16 SNPs	16 SNPs	100% identical	16 SNPs	16 SNPs	100% identical	16 SNPs	16 SNPs	16 SNPs	3 SNPs
Protein	-	2 cs & 2 ncs aa subs	2 cs & 2 ncs aa subs	2 cs & 2 ncs aa subs	-	2 cs & 2 ncs aa subs	2 cs & 2 ncs aa subs	-	2 cs & 2 ncs aa subs	2 cs & 2 ncs aa subs	2 cs & 2 ncs aa subs	2 cs & 1 ncs aa subs
Haplotype	H2	H1	H1	H1	H2	H1	H1	H2	H1	H1	H1	H3
**TaNAS8-B1**	**TraesCS6B03G1227100**	**TraesARI6B03G03592370**	**TraesJUL6B03G03662750**	**TraesLAC6B03G03583300**	**TraesNOR6B03G03669280**	**TraesSYM6B03G03574130**	**TraesMAC6B03G03629250**	**TraesTSP6B01G478100**	**TraesJAG6B03G03622080**	**TraesLDM6B03G03631140**	**TraesSTA6B03G03620490**	**6B:704833079-704832093 Reverse**
Gene	-	100% identical	100% identical	33 SNPs, 3 nt ins	100% identical	34 SNPs, 3 nt ins	100% identical	31 SNPs, 3 nt ins	31 SNPs, 3 nt ins	34 SNPs, 3 nt ins	100% identical	100% identical
Protein	-	-	-	2 cs & 7 ncs aa subs, 1 aa ins	-	2 cs & 10 ncs aa subs, 1 aa ins, 1 aa del	-	1 cs & 7 ncs aa subs, 1 aa ins	1 cs & 7 ncs aa subs, 1 aa ins	2 cs & 10 ncs aa subs, 1 aa ins, 1 aa del	-	-
Haplotype	H1	H1	H1	H4	H1	H3	H1	H2	H2	H3	H1	H1

**TaNAS8-D1**	**TraesCS6D03G881800**	**TraesARI6D03G03764430**	**TraesJUL6D03G03828250**	**TraesLAC6D03G03745810**	**TraesNOR6D03G03835840**	**TraesSYM6D03G03742780**	**TraesMAC6D03G03793230**	**TraesTSP6D01G412500**	**TraesJAG6D03G03777380**	**TraesLDM6D03G03799190**	**TraesSTA6D03G03787970**	**6D:462354308-462355294 Forward**
Gene	-	100% identical	100% identical	33 SNPs	100% identical	100% identical	33 SNPs	33 SNPs	33 SNPs	100% identical	33 SNPs	26 SNPs
Protein	-	-	-	7 cs & 6 ncs aa subs	-	-	7 cs & 6 ncs aa subs	7 cs & 6 ncs aa subs	7 cs & 6 ncs aa subs	-	7 cs & 6 ncs aa subs	6 cs & 4 ncs aa subs
Haplotype	H1	H1	H1	H2	H1	H1	H2	H2	H2	H1	H2	H3
**TaNAS9-A1**	**TraesCS2A03G196300**	**TraesARI2A03G00615350**	**TraesJUL2A03G00611970**	**TraesLAC2A03G00612520**	**TraesNOR2A03G00616330**	**TraesSYM2A03G00614950**	**TraesMAC2A03G00607040**	**TraesTSP2A01G104200**	**TraesJAG2A03G00607290**	**TraesLDM2A03G00610790**	**TraesSTA2A03G00607220**	**2A:49221108-49222130 Forward**
Gene	-	100% identical	100% identical	100% identical	100% identical	100% identical	100% identical	100% identical	33 nt 5' trunation	100% identical	100% identical	100% identical
Protein	-	-	-	-	-	-	-	-	11 aa N-term trunation	-	-	-
Haplotype	H1	H1	H1	H1	H1	H1	H1	H1	H2	H1	H1	H1
**TaNAS9-B1**	**TraesCS2B03G264600**	**TraesARI2B03G00868570**	**TraesJUL2B03G00862760**	**TraesLAC2B03G00854580**	**TraesNOR2B03G00869150**	**TraesSYM2B03G00868510**	**TraesMAC2B03G00856020**	**TraesTSP2B01G123800**	**TraesJAG2B03G00857470**	**TraesLDM2B03G00859510**	**TraesSTA2B03G00858670**	**2B:72895158-72896180 Forward**
Gene	-	19 SNPs	18 SNPs	100% identical	100% identical	18 SNPs	100% identical	100% identical	100% identical	100% identical	100% identical	100% identical
Protein	-	4 cs & 2 ncs aa subs	4 cs & 2 ncs aa subs	-	-	4 cs & 2 ncs aa subs	-	-	-	-	-	-
Haplotype	H1	H3	H2	H1	H1	H2	H1	H1	H1	H1	H1	H1
**TaNAS9-D1**	**TraesCS2D03G196500**	**TraesARI2D03G01126000**	**TraesJUL2D03G01115740**	**TraesLAC2D03G01061350**	**TraesNOR2D03G01125590**	**TraesSYM2D03G01123730**	**TraesMAC2D03G01108120**	**TraesTSP2D01G110200**	**TraesJAG2D03G01112430**	**TraesLDM2D03G01110830**	**TraesSTA2D03G01098320**	**2D:45799198-45800220 Forward**
Gene	-	100% identical	100% identical	100% identical	100% identical	33 nt trun (5')	100% identical	100% identical	100% identical	33 nt trun (5')	33 nt trun (5')	100% identical
Protein	-	-	-	-	-	11 aa trun (N-ter)	-	-	-	11 aa trun (N-ter)	11 aa trun (N-ter)	-

Haplotype	H1	H1	H1	H1	H1	H2	H1	H1	H1	H2	H2	H1
**TaNAS10-A1**	**TraesCS3A03G1088500**	**TraesARI3A03G01521050**	**TraesJUL3A03G01511990**	**TraesLAC3A03G01443470**	**TraesNOR3A03G01520940**	**TraesSYM3A03G01522450**	**TraesMAC3A03G01497740**	**TraesTSP3A01G496900**	**TraesJAG3A03G01508120**	**TraesLDM3A03G01499820**	**TraesSTA3A03G01491250**	**3A:701211258-701210236 Reverse**
Gene	-	100% identical	2 SNPs	100% identical	100% identical	100% identical	100% identical	2 SNPs	100% identical	100% identical	100% identical	100% identical
Protein	-	-	1 cs aa sub	-	-	-	-	2 ncs aa subs	-	-	-	-
Haplotype	H1	H1	H2	H1	H1	H1	H1	H3	H1	H1	H1	H1
	Chinese Spring	ArinaLrFor	Julius	Lancer	Norin61	SY Mattis	Mace	Triticum spelta	Jagger	Landmark	Stanley	Fielder
**TaNAS2-B3**	**-**	**-**	**TraesJUL6B03G03521280**	**-**	**-**	**TraesSYM6B03G03437270**	**-**	**-**	**TraesJAG6B03G03488360**	**-**	**TraesSTA6B03G03488710**	**-**
Gene	-	-	-	-	-	-	-	-	1 SNP	-	1 SNP	-
Protein	-	-	-	-	-	-	-	-	1 cs aa sub	-	1 cs aa sub	-
Haplotype	-	-	H1	-	-	H1	-	-	H2	-	H2	-
**TaNAS4-A3**	**-**	**TraesARI5A03G02826920**	**-**	**-**	**-**	**TraesSYM5A03G02817720**	**-**	**-**	**-**	**-**	**-**	**-**
Gene	-	-	-	-	-	-	-	-	-	-	-	-
Protein	-	-	-	-	-	-	-	-	-	-	-	-
Haplotype	-	H1	-	-	-	H1	-	-	-	-	-	-
**TaNAS5-B3**	**-**	**TraesARI3B03G01580520**	**TraesJUL3B03G01569830**	**TraesLAC3B03G01499120**	**TraesNOR3B03G01579400**	**TraesSYM3B03G01580630**	**TraesMAC3B03G01556900**	**TraesTSP3B01G085200**	**TraesJAG3B03G01566170**	**TraesLDM3B03G01558230**	**TraesSTA3B03G01550140**	**3B:40569853-40570395 Forward**
Gene	-	-	105 nt ext (5')	-	-	-	-	-	-	7 SNPs	7 SNPs	-
Protein	-	-	35 aa ext (N-ter)	-	-	-	-	-	-	2 cs & 2 ncs aa subs	2 cs & 2 ncs aa subs	-
Haplotype	-	H1	H3	H1	H1	H1	H1	H1	H1	H2	H2	H1

**TaNAS8-B2**	**-**	**-**	**-**	**-**	**-**	**TraesSYM6B03G03574110**	**-**	**-**	**-**	**-**	**-**	**-**
Gene	-	-	-	-	-	-	-	-	-	-	-	-
Protein	-	-	-	-	-	-	-	-	-	-	-	-
Haplotype	-	-	-	-	-	H1	-	-	-	-	-	-

Supplementary Table S6. Grain Fe, Zn, and NA fold-differences between OE-*TaNAS* and NS plants at the T_3 and T_4 generations. Fold differences (bold) in grain Fe, Zn and NA concentrations between OE-*TaNAS* events and their respective NS with p-values as determined by a two-sample Student's t-test assuming unequal variance shown in the adjacent column. Underlined values indicated statistically significant differences where p < 0.05.

	2022 glasshouse				2022 field				2023 field	
**Event**	**Fe**	**p value**	**Zn**	***p* value**	**Fe**	***p* value**	**Zn**	***p* value**	**NA**	***p* value**
OE-*TaNAS1-1*	**0.898**	0.315	**0.868**	0.253					**0.746**	0.185
OE-*TaNAS1-2*	**0.909**	0.278	**0.898**	0.156					**0.832**	0.430
OE-*TaNAS1-3*	**1.003**	0.976	**1.000**	1.000	**1.114**	0.140	**1.137**	0.382	**1.056**	0.607
OE-*TaNAS3-1*	**1.248**	0.050	**1.145**	0.146					**1.247**	0.222
OE-*TaNAS3-2*	**1.506**	0.003	**1.512**	0.001	**1.288**	0.005	**1.240**	0.165	**1.279**	0.101
OE-*TaNAS3-3*	**1.121**	0.145	**1.166**	0.027					**0.980**	0.902
OE-*TaNAS4-1*	**1.089**	0.346	**1.057**	0.257	**0.788**	0.241	**0.967**	0.745	**1.273**	0.098
OE-*TaNAS4-2*	**1.068**	0.363	**1.044**	0.517					**0.980**	0.847
OE-*TaNAS4-3*	**0.962**	0.452	**0.980**	0.710					**0.915**	0.654
OE-*TaNAS6-1*	**0.973**	0.746	**1.177**	0.013					**1.361**	0.129
OE-*TaNAS6-2*	**1.292**	0.002	**1.315**	0.003					**2.032**	0.085
OE-*TaNAS6-3*	**0.840**	0.462	**1.113**	0.010	**1.092**	0.226	**1.289**	0.206	**1.555**	0.081
OE-*TaNAS6S-1*	**1.038**	0.728	**1.196**	0.025	**1.217**	0.132	**1.396**	0.015	**3.702**	0.013
OE-*TaNAS6S-2*	**1.109**	0.225	**1.255**	0.018					**0.924**	0.495
OE-*TaNAS6S-3*	**1.550**	0.020	**1.746**	0.000					**1.364**	0.008
OE-*TaNAS7-1*	**0.950**	0.407	**0.938**	0.460	**1.109**	0.419	**0.986**	0.839	**0.890**	0.273
OE-*TaNAS7-2*	**1.238**	0.037	**1.309**	0.017					**0.950**	0.612
OE-*TaNAS7-3*	**0.793**	0.425	**1.546**	0.001	**1.294**	0.002	**1.290**	0.101	**1.746**	0.017

Supplementary Table S7. Grain P, Mn, and Cu fold-differences between OE-*TaNAS* and NS plants at the T_3 generation. The table contains fold differences (bolded) of OE-*TaNAS* and their respective NS with p-values as determined by a two-sample Student's t-test assuming unequal variance shown in the adjacent column. Underlined values indicated statistically significant differences where $p < 0.05$.

	**2022 glasshouse**						**2022 field**					
**Event**	**P**	***p* value**	**Mn**	***p* value**	**Cu**	***p* value**	**P**	***p* value**	**Mn**	***p* value**	**Cu**	***p* value**
OE-*TaNAS1-1*	**0.995**	0.934	**0.927**	0.264	**0.931**	0.265						
OE-*TaNAS1-2*	**0.873**	0.050	<u>**0.884**</u>	<u>0.026</u>	<u>**0.782**</u>	<u>0.001</u>						
OE-*TaNAS1-3*	**0.970**	0.632	**0.977**	0.685	**0.696**	0.173	**1.020**	0.760	**1.137**	0.158	**1.212**	0.076
OE-*TaNAS3-1*	**0.995**	0.941	**0.967**	0.592	**1.021**	0.863						
OE-*TaNAS3-2*	**1.176**	0.143	<u>**1.182**</u>	<u>0.020</u>	<u>**1.368**</u>	<u>0.012</u>	**1.041**	0.610	**1.024**	0.642	**1.117**	0.509
OE-*TaNAS3-3*	**0.959**	0.407	**0.964**	0.412	**1.003**	0.969						
OE-*TaNAS4-1*	**1.171**	0.122	**1.065**	0.193	**1.068**	0.675	**1.029**	0.590	**0.978**	0.841	**0.965**	0.747
OE-*TaNAS4-2*	**1.025**	0.705	**1.045**	0.331	**0.781**	0.480						
OE-*TaNAS4-3*	**0.900**	0.218	**1.018**	0.717	**0.932**	0.557						
OE-*TaNAS6-1*	**0.958**	0.632	**1.032**	0.431	<u>**0.634**</u>	<u>0.020</u>						
OE-*TaNAS6-2*	**1.047**	0.529	**1.085**	0.408	**1.146**	0.302						
OE-*TaNAS6-3*	<u>**0.884**</u>	<u>0.007</u>	**0.927**	0.204	**0.828**	0.681	**1.039**	0.562	**0.965**	0.817	**1.068**	0.710
OE-*TaNAS6S-1*	**0.859**	0.173	**0.971**	0.612	**0.919**	0.501	**0.980**	0.649	**0.986**	0.924	**1.230**	0.176
OE-*TaNAS6S-2*	**0.888**	0.236	**1.022**	0.814	**0.981**	0.835						
OE-*TaNAS6S-3*	**1.227**	0.058	**1.156**	0.088	<u>**1.387**</u>	<u>0.021</u>						
OE-*TaNAS7-1*	**0.881**	0.200	**0.951**	0.519	**0.913**	0.314	**1.000**	1.000	**1.006**	0.967	**0.913**	0.373
OE-*TaNAS7-2*	**0.947**	0.590	**0.990**	0.904	**0.931**	0.568						
OE-*TaNAS7-3*	**1.109**	0.238	**1.153**	0.059	**1.088**	0.252	**1.042**	0.465	**0.991**	0.940	**1.269**	0.091

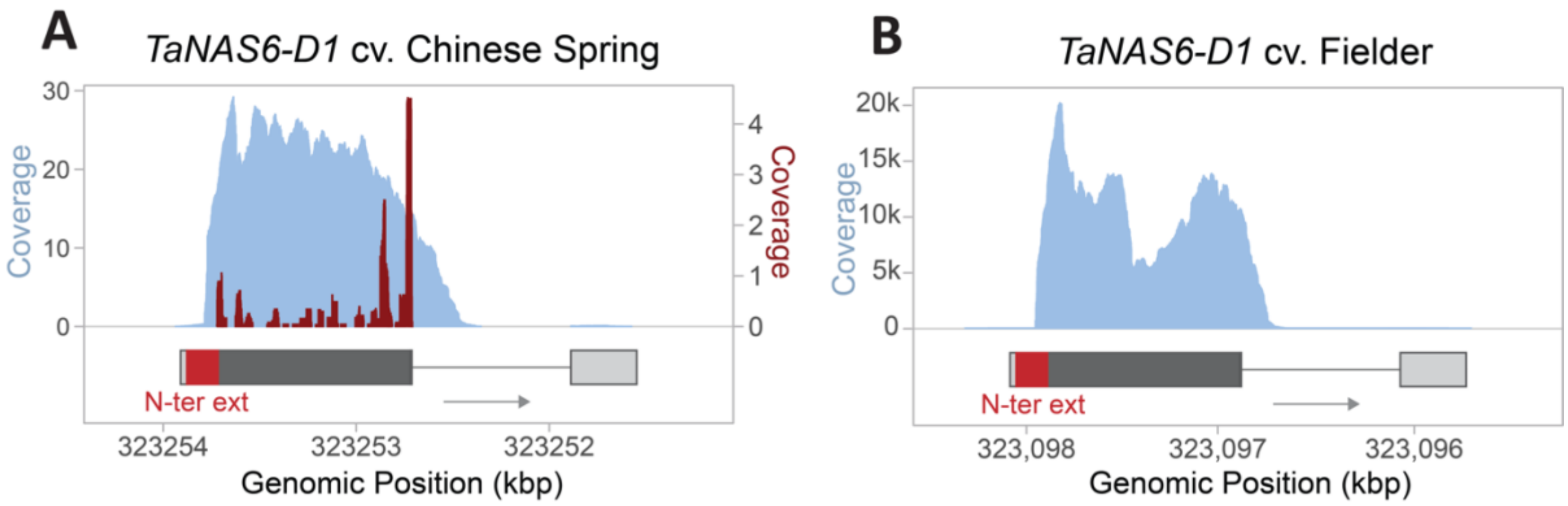

Supplementary Figure S1. Ribosomal profile and RNA-seq coverage of *TaNAS6-D1*. Histograms of overlapping Ribo-seq (red; right y-axis) and RNA-seq (blue; left y-axis) of *TaNAS6-D1* homoeologs in (a) developing grain five days post anthesis from cv. Chinese Spring and (b) roots of cv. Fielder 11 days after Fe deficiency. Coverage in (a) is normalised to reads per million mapped to nuclear coding sequences, with data sourced from (Guo et al., 2023), while (b) shows the raw read count mapped to nuclear coding sequences produced within this study. Arrows indicate gene orientation (5' to 3'). The *TaNAS6-D1* gene model is predicted by IWGSC Refseq2.0 with the 5' and 3' UTRs (light grey rectangles), coding sequences (dark grey rectangles), and introns (horizontal lines) indicated. The 162 bp N-terminus extension (red rectangle) predicted in the cv. Chinese Spring *TaNAS6-D1* gene by the IWGSC Refseq1.0 and 2.0 annotation is highlighted.

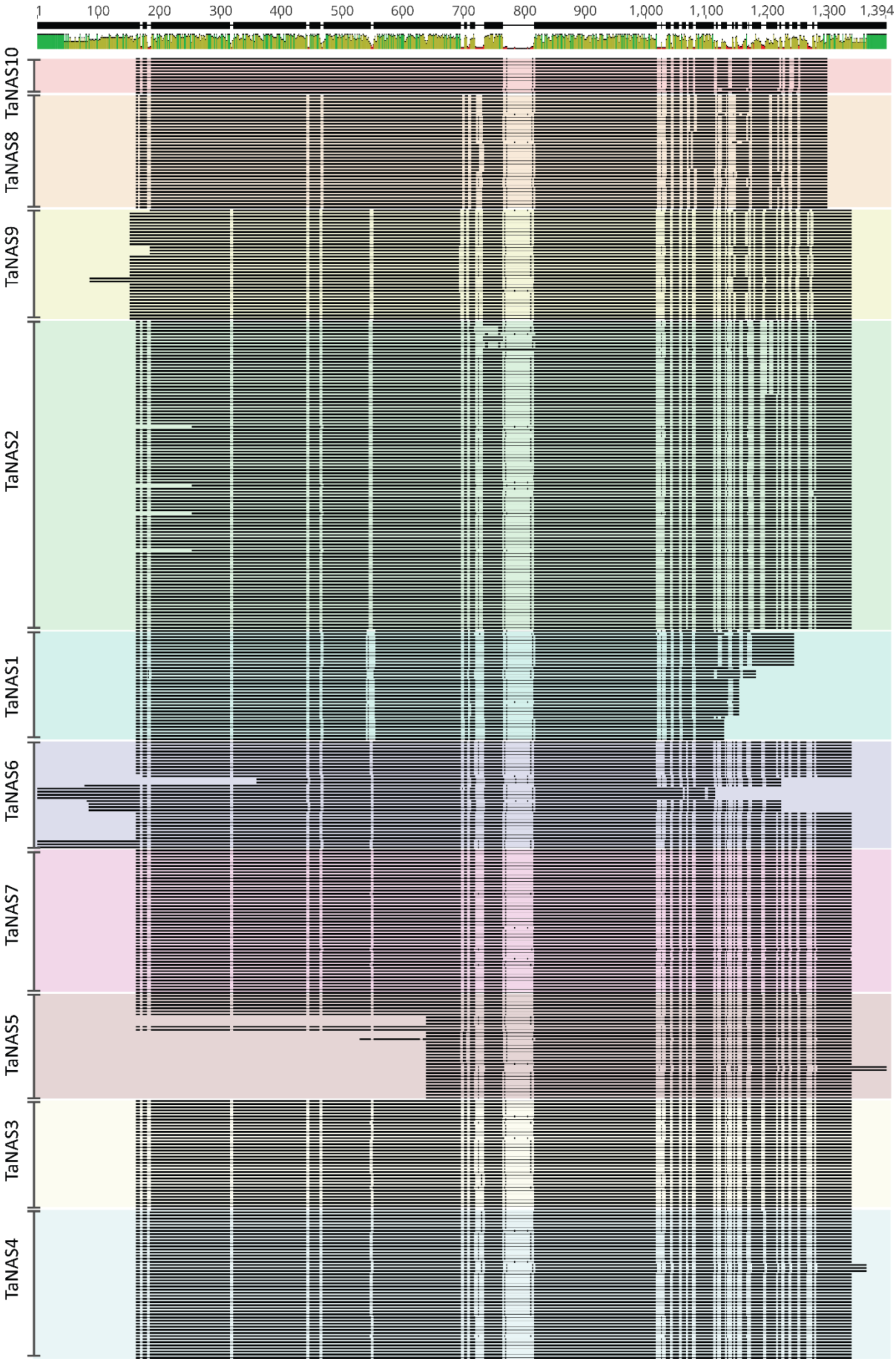

1
100
200
300
400
500
600
700
800
900
1,000
1,100
1,200
1,300
1,394
TaNAS10
TaNAS8
TaNAS9
TaNAS2
TaNAS1
TaNAS6
TaNAS7
TaNAS5
TaNAS3
TaNAS4

Supplementary Figure S2. Alignment of 425 *TaNAS* coding sequences from cvs. Chinese Spring, ArinaLrFor, Julius, Lancer, Norin61, SY Mattis, Mace, Jagger, Landmark, Stanley, Fielder, and *Triticum spelta* L. The alignment was performed using Geneious alignment tool. The top bar indicates consensus (black bars) and high (green), medium (yellow), and low (red) identity indicated as a histogram. The nucleotide positions indicated. Each coloured segment represents a *TaNAS* grouping as determined by the PhyML Geneious plugin with the K80 substitution model and 100 bootstrap replications.

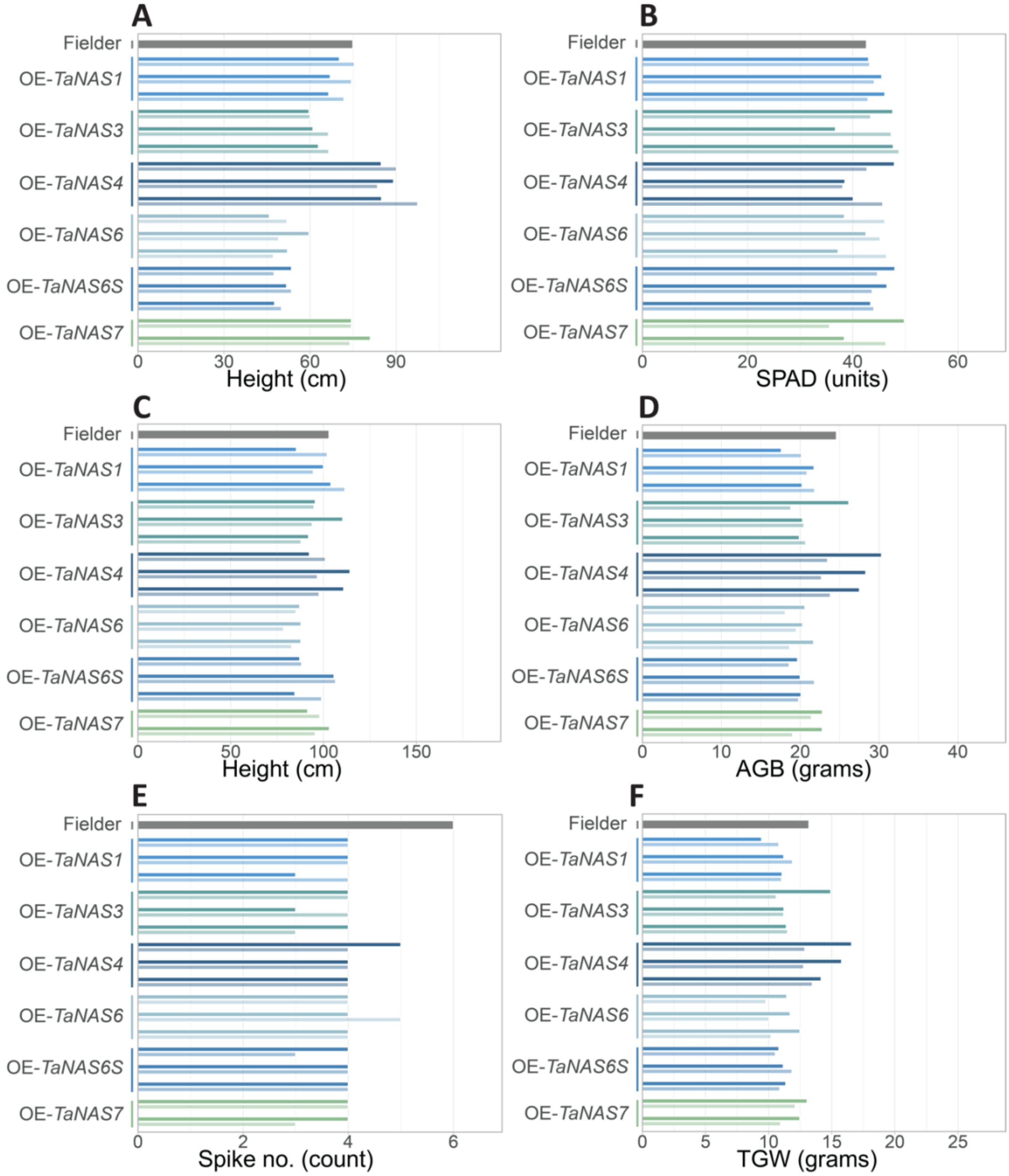

A
Fielder
OE-TaNAS1
OE-TaNAS3
OE-TaNAS4
OE-TaNAS6
OE-TaNAS6S
OE-TaNAS7
0
30
60
90
Height (cm)
B
SPAD (units)
0
20
40
60
C
Height (cm)
0
50
100
150
D
AGB (grams)
0
10
20
30
40
E
Spike no. (count)
0
2
4
6
F
TGW (grams)
0
5
10
15
20
25

Fig S3. cont.

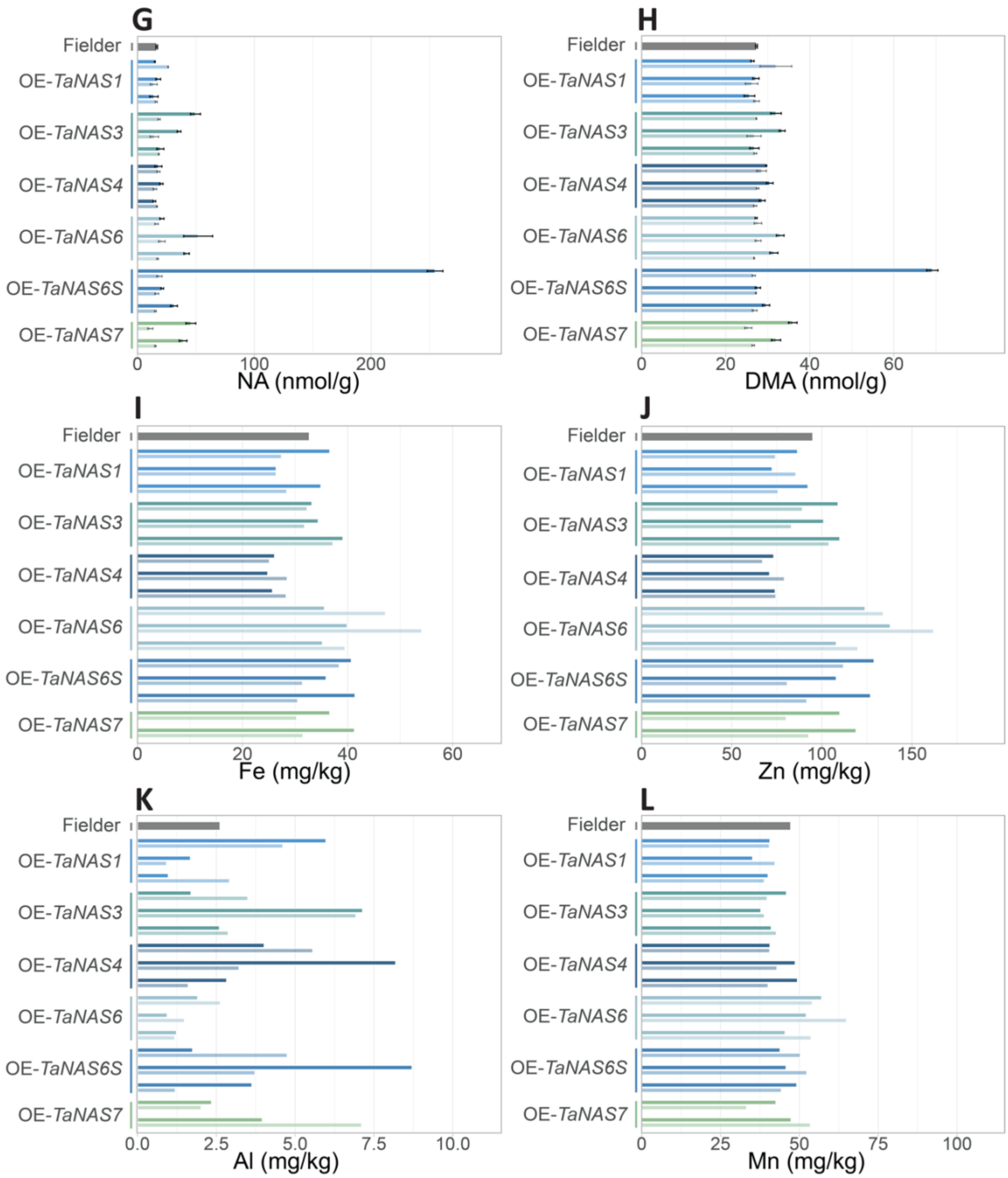

Supplementary Figure S3. Phenotypic and nutritional analysis of T_1 OE-*TaNAS* and NS plants in the glasshouse. Plant (a) height (cm) and (b) SPAD of three month-old plants; (c) height (cm), (d) above ground biomass (AGB, g), (e) spike number, and (f) total grain weight (TGW, g) of mature plants; and grain (g) nicotianamine (NA, nmol/g), (h) 2'deoxymugeneic acid (DMA, nmol/g), (i) Fe (mg/kg), (j) Zn (mg/kg), (k) aluminium (Al, mg/kg), and (l) manganese (Mn, mg/kg) of OE-*TaNAS1* (blue), OE-*TaNAS3* (teal), OE-*TaNAS4* (navy blue), OE-*TaNAS6* (light blue), OE-*TaNAS6S* (dark blue) and OE-*TaNAS7* (green) lines, respective null segregant (faded colour of each), and cv. Fielder (grey) in the glasshouse in 2021. Events 1, 2 and 3 for each OE-*TaNAS* line are shown at the top, middle and bottom, respectively (n=1). Error bars for NA and DMA concentrations represent the standard error of three technical replicates.

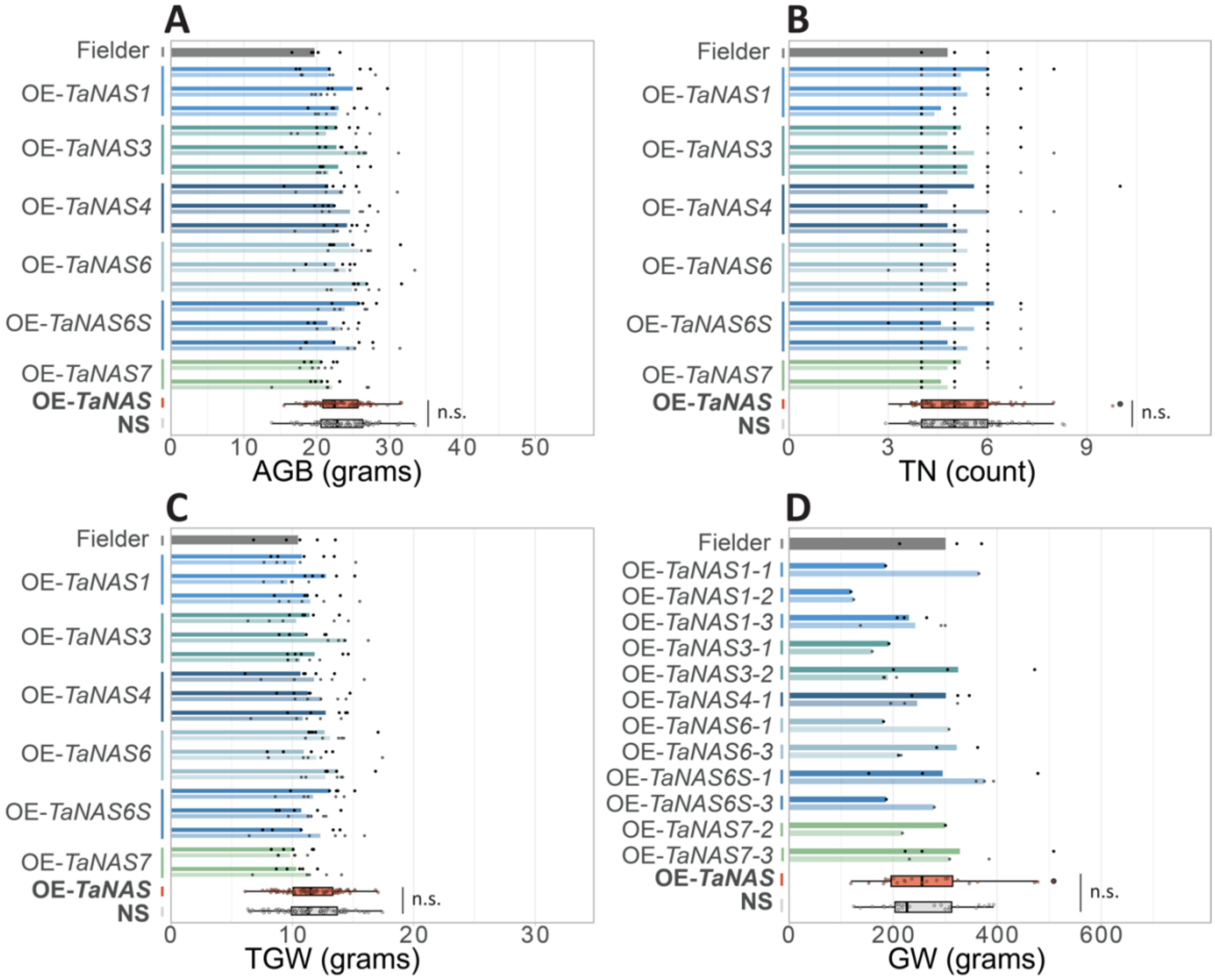

Supplementary Figure S4. Agronomic traits of T_3 OE-*TaNAS* and NS plants in the glasshouse and field. Plant (a) above ground biomass (AGB, g), (b) tiller number per plant (TN/plant), (c) total grain weight (TGW, g) from glasshouse (GH) grown plants, and (d) subsampled grain weight (GW, g) of field grown plants of OE-*TaNAS1* (blue), OE-*TaNAS3* (teal), OE-*TaNAS4* (navy blue), OE-*TaNAS6* (light blue), OE-*TaNAS6S* (dark blue) and OE-*TaNAS7* (green) lines, respective null segregant (NS; faded colour of each), and cv. Fielder (grey) in the glasshouse in 2022. Events 1, 2 and 3 for each OE-*TaNAS* line are shown at the top, middle and bottom, respectively (n=5). Statistical differences between OE-*TaNAS* events and their respective NS are shown as p values determined by a two-sample Student's t-test assuming unequal variance with a 95% confidence interval. The bars represent the average of one, three, or five biological replicates with the raw datapoints overlaid. The box plots represent all OE-*TaNAS* (orange) values compared with all NS (grey) values. Non-significant (n.s.) values between all OE-*TaNAS* and all NS values as determined by a two-sample Student's t-test assuming unequal variance are indicated.

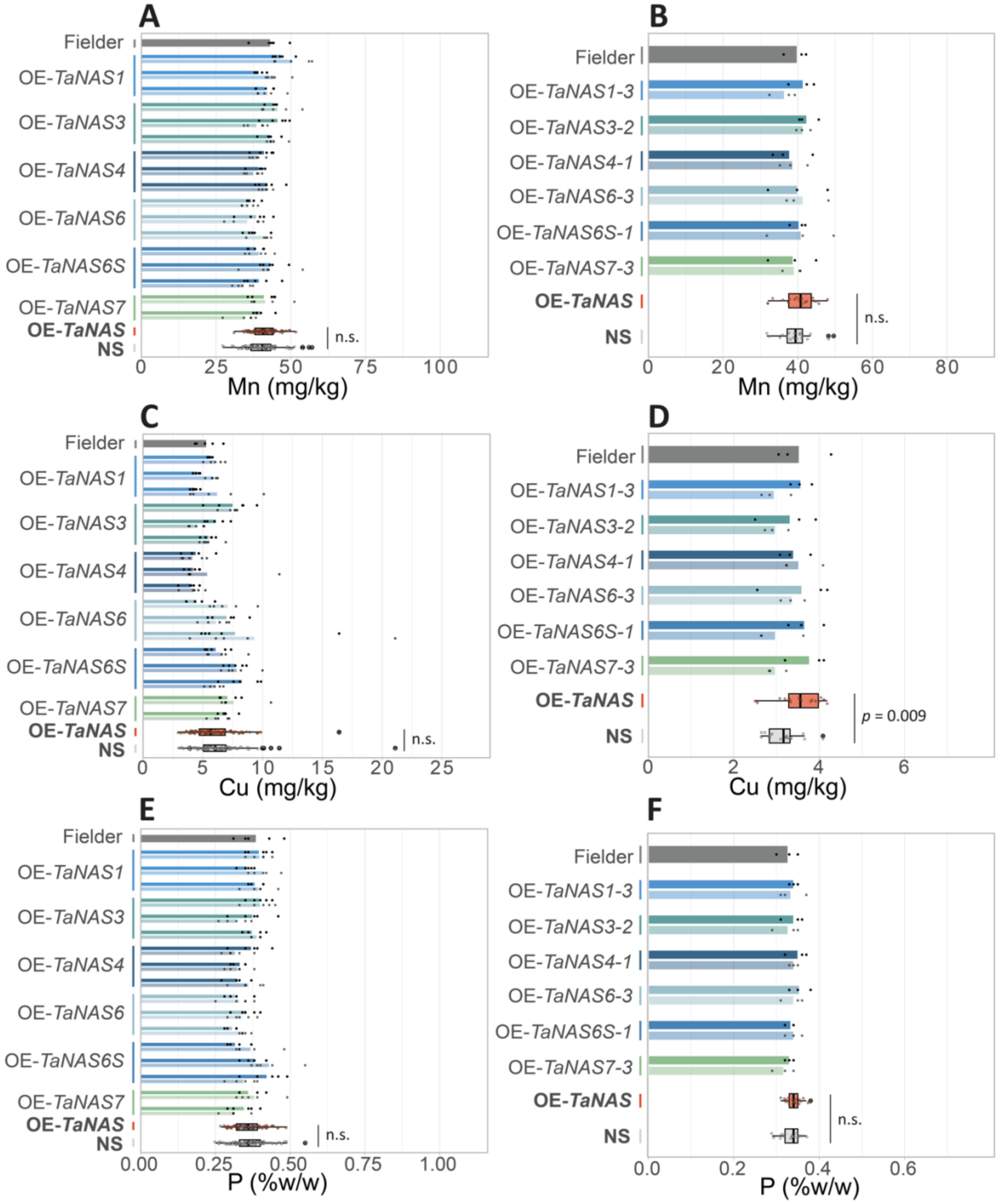

Supplementary Figure S5. Grain Mn, Cu, and P concentrations of T_3 OE-*TaNAS* and NS plants in glasshouse and field experiments. Grain (a, b) manganese (Mn, mg/kg), (c, d) copper (Cu, mg/kg), (e, f) phosphorus (P, %w/w) concentrations of OE-*TaNAS1* (blue), OE-*TaNAS3* (teal), OE-*TaNAS4* (navy blue), OE-*TaNAS6* (light blue), OE-*TaNAS6S* (dark blue) and OE-*TaNAS7* (green) lines, respective null segregants (NS; faded colour of each), and cv. Fielder (grey) in the glasshouse and field in 2022. For the glasshouse experiment (a, c, e) events 1, 2 and 3 for each OE-*TaNAS* line are shown at the top, middle and bottom, respectively (n=5). For the field experiment (b, d, f), events OE-*TaNAS1-3*, OE-*TaNAS3-2*, OE-*TaNAS4-1*, OE-*TaNAS6-3*, OE-*TaNAS6S-1*, and OE-*TaNAS7-3* are shown (n=3). Statistical differences between OE-*TaNAS* events and their respective NS are shown as *p* values determined by a two-sample Student's t-test assuming unequal variance with a 95%

confidence interval. The bars represent the average of three or five biological replicates with the raw datapoints overlaid. The box plots represent all OE-*TaNAS* (orange) values compared with all NS (grey) values. Non-significant (n.s.) values between all OE-*TaNAS* and all NS values as determined by a two-sample Student's t-test assuming unequal variance are indicated.
